# Measures of evidence-informed decision-making competence attributes: a psychometric systematic review

**DOI:** 10.1186/s12912-020-00436-8

**Published:** 2020-05-27

**Authors:** Emily Belita, Janet E. Squires, Jennifer Yost, Rebecca Ganann, Trish Burnett, Maureen Dobbins

**Affiliations:** 1 McMaster University, School of Nursing, McMaster Innovation Park (MIP), 175 Longwood Road South, Suite 210a, Hamilton, ON L8P 0A1 Canada; 2 University of Ottawa/Université d’Ottawa, School of Nursing/École des sciences infirmières, Room RGN 3038, Guindon Hall, 451 Smyth Road, Ottawa, ON Canada; 3 Villanova University, M. Louise Fitzpatrick College of Nursing, Driscoll Hall, Room 330, 800 Lancaster Avenue, Villanova, PA 19085 USA; 4 McMaster University, School of Nursing, 1280 Main St. W., HSC 3N25F, Hamilton, ON Canada

**Keywords:** Evidence-informed decision-making, Nursing, Evidence-based practice, Psychometrics, Competence assessment

## Abstract

**Background:**

The current state of evidence regarding measures that assess evidence-informed decision-making (EIDM) competence attributes (i.e., knowledge, skills, attitudes/beliefs, behaviours) among nurses is unknown. This systematic review provides a narrative synthesis of the psychometric properties and general characteristics of EIDM competence attribute measures in nursing.

**Methods:**

The search strategy included online databases, hand searches, grey literature, and content experts. To align with the Cochrane Handbook of Systematic Reviews, psychometric outcome data (i.e., acceptability, reliability, validity) were extracted in duplicate, while all remaining data (i.e., study and measure characteristics) were extracted by one team member and checked by a second member for accuracy. Acceptability data was defined as measure completion time and overall rate of missing data. The Standards for Educational and Psychological Testing was used as the guiding framework to define reliability, and validity evidence, identified as a unified concept comprised of four validity sources: content, response process, internal structure and relationships to other variables. A narrative synthesis of measure and study characteristics, and psychometric outcomes is presented across measures and settings.

**Results:**

A total of 5883 citations were screened with 103 studies and 35 unique measures included in the review. Measures were used or tested in acute care (*n* = 31 measures), public health (*n* = 4 measures), home health (n = 4 measures), and long-term care (*n* = 1 measure). Half of the measures assessed a single competence attribute (*n* = 19; 54.3%). Three measures (9%) assessed four competence attributes of knowledge, skills, attitudes/beliefs and behaviours. Regarding acceptability, overall missing data ranged from 1.6–25.6% across 11 measures and completion times ranged from 5 to 25 min (n = 4 measures). Internal consistency reliability was commonly reported (21 measures), with Cronbach’s alphas ranging from 0.45–0.98. Two measures reported four sources of validity evidence, and over half (n = 19; 54%) reported one source of validity evidence.

**Conclusions:**

This review highlights a gap in the testing and use of competence attribute measures related to evidence-informed decision making in community-based and long-term care settings. Further development of measures is needed conceptually and psychometrically, as most measures assess only a single competence attribute, and lack assessment and evidence of reliability and sources of established validity evidence.

**Registration:**

PROSPERO #CRD42018088754.

## Background

Nurses play an important role in ensuring optimal health outcomes by engaging in evidence-informed decision making (EIDM). EIDM, used synonymously with the term evidence-based practice (EBP) [1] involves “the conscientious, explicit, and judicious use of current best evidence in making decisions about the care of individual patients” [2] (p. 71). The use of the word ‘informed’ in EIDM denotes that research alone is insufficient for clinical decision making and cannot take precedence over other factors [3]. Evidence in this regard then, is defined as credible knowledge from different sources including research, professional/clinical experience, patient experiences/preferences, and local data and information [4, 5]. There are numerous examples of improved patient outcomes following implementation of best practice guidelines such as reductions in length of hospital stay [6] and adverse patient events related to falls and pressure ulcers in long-term care settings [7].

Despite knowledge of such benefits, competency gaps and low implementation rates in EIDM persist among nurses across diverse practice settings [8–10]. A barrier to EIDM implementation has been the lack of clarity and understanding about what nurses should be accountable for with respect to EIDM as well as how it can be best measured [11, 12]. As such, considerable effort has occurred in the development of EIDM competence measures as a strategy to support EIDM implementation in nursing practice [12].

EIDM competence attributes of knowledge, skills, attitudes/beliefs, and behaviours have been well defined in the literature. EIDM knowledge is an understanding of the primary concepts and principles of EIDM and hierarchy of evidence [13–17]. Skills in EIDM refer to the application of knowledge required to complete EIDM tasks (e.g., developing a comprehensive strategy to search for research evidence) [13–17]. Attitudes and beliefs related to EIDM include perceptions, beliefs, and values ascribed to EIDM (e.g., belief that EIDM improves patient outcomes) [13, 15]. EIDM behaviours are defined by the performance of EIDM steps in real-life clinical practice (e.g., identifying a clinical problem to be addressed) [13, 15, 17].

Multiple uses for measures assessing EIDM competence attributes in nursing practice and research exist. Such measures can be integrated into performance appraisals [18] to monitor progressive changes in overall EIDM competence or specific domains. At an organizational level, EIDM competence standards can support human resource management by establishing clear EIDM role expectations for prospective, newly hired, or employed nurses [18, 19]. With respect to nursing research, there has been great attention afforded to the development and testing of different interventions to increase EIDM knowledge, attitudes, skills, and behaviours among nurses [20–22]. The use of EIDM competence instruments that produce valid and reliable scores can help to ascertain effective interventions in developing EIDM competence areas.

Previous systematic reviews have focused on EIDM competence attribute measures used among allied health care professionals [13, 16, 23] as well as nurses and midwives [14]. However, several limitations exist among these reviews. A conceptual limitation is that many reviews included research utilization measures despite stating a focus on EIDM [13, 14, 23]. Research utilization, while considered a component of EIDM, is conceptually distinct from it. Research utilization includes the use of scientific research evidence in health care practice [24]. While, EIDM encompasses the application of multiple forms of evidence such as clinical experience, patient preferences, and local context or setting [5]. Conceptual clarity is of critical importance in a psychometric systematic review, as it can impact findings of reported validity evidence. Reviews by Glegg and Holsti [16] and Leung et al. [14] were also limited in focus, as they included measures that assessed only a few, but not all four of the attributes that comprise competence, potentially resulting in the exclusion of existing EIDM measures. Methodologically, across all reviews, psychometric assessment was limited as validity evidence was either not assessed [16] or assessed only by reviewing data that was formally reported as content, construct, or criterion validity [13, 14, 23], neglecting other critical data that could support validity evidence of a measure. As well, none of the reviews reported on or extracted data on specific practice settings. This is an essential component of psychometric assessment, as Streiner et al. [25] identify that reliability and validity are contingent not solely on scale properties, but on the sample with whom and specific situation in which measures are tested. Consideration of setting is important when determining the applicability of a measure for a specific population due to differences in role and environment. Despite these existing reviews, most importantly, none of them focused only on nurses. A systematic review unique to nursing is imperative given the diversity of needs, reception to, and expectations of EIDM across health care professional groups [16]. These differences may be reflected across measures to assess discipline specific EIDM competence.

The current review aimed to address limitations of existing reviews by: including measures that address a holistic conceptualization of EIDM which includes the use of multiple forms of evidence in nursing practice; focusing on the four EIDM competence attributes of knowledge, skills, attitudes and behaviours; utilizing a modern understanding of validity evidence in which sources based on test content, response process, internal structure, and relations to other variables were assessed according to the Standards for Educational and Psychological Testing [26]; extracting data on and presenting findings within the context of practice setting; and targeting the unique population of nurses.

The objectives of this systematic review were to: 1) identify existing measures of EIDM competence attributes of knowledge, skills, attitudes/beliefs, and/or behaviours used among nurses in any healthcare setting; and 2) determine the psychometric properties of test scores for these existing measures.

## Methods

The protocol for this systematic review was registered (PROSPERO #CRD42018088754), was published [27] a priori, and followed the Preferred Reporting Items for Systematic Reviews and Meta-Analyses (PRISMA) guideline.

### Search strategy

A comprehensive search strategy consisting of online databases, hand searches, grey literature, and content experts, was developed in consultation with a Health Sciences Librarian. Searches were limited from 1990 until December 2017, as the term evidence-based medicine was first introduced and defined in 1990 [28]. Search strategy sources are summarized in Table 1. A detailed search strategy is provided in Additional file 1.

**Table 1:** Search strategy

Electronic databases (inception until December, 6, 2017)	
• Cumulative Index to Nursing and Allied Health Literature (CINAHL)	
• EMBASE	
• Education Resources Information Centre (ERIC)	
• Health and Psychological Instruments (HaPI)	
• MathSciNet	
• Ovid Medline	
Other sources:	
• Hand searches of included studies	
• Hand searches of relevant journals including Implementation Science and Worldviews on Evidence Based Nursing	
• Grey Literature Report (http://greylit.org/)	
• Canadian Health Research Collection	
• Nursing association resource portals	
o Canadian Nurses Association (https://www.cna-aiic.ca/en)	
o Community Health Nurses of Canada (https://www.chnc.ca/)	
o American Nurses Association (https://www.nursingworld.org/)	
• Four content experts with high frequency citations related to EIDM assessment	
• Relevant conference proceedings:	
o Annual Conference on the Science of Dissemination and Implementation in Health (https://cancercontrol.cancer.gov/IS/training-education/index.html#conference)	
o National Community Health Nursing Conference – Community Health Nurses of Canada (https://www.chnc.ca/en/conferences)	
o Knowledge Translation Canada Annual Scientific Meeting	

### Inclusion and exclusion criteria

Studies were included if they met the following criteria: study sample consists of all nurses or a portion of nurses; conducted in any healthcare setting; reported findings from the use or psychometric testing of measures that assesses EIDM knowledge, skills, attitudes/values, and/or behaviours; quantitative or mixed-method design; and English language. Studies were excluded if the sample consisted of solely other healthcare professionals or nursing undergraduate students, or in which data specific to nurses was not reported separately. As well, studies testing or using measures assessing research utilization were excluded [5, 24].

### Study selection

Titles and abstracts of initial references and full-text records were screened independently by two team members (EB and TB) for inclusion/exclusion. All disagreements were able to be resolved by consensus between those whom extracted the data.

### Data extraction

Data extraction was piloted using a standard form completed independently by two team members (EB and TB) on five randomly selected references. Data extracted pertaining to study and measure characteristics included: study design, sample size, professional designation of sample, healthcare setting, study country, funding, name of measure, format, purpose of measure, item development process, number of items, theoretical framework used, conceptual definition of competence established, EIDM attributes measured, EIDM domains/steps covered, and marking key or scale for self-report measures. Data extraction on these characteristics was performed by one team member (EB) and checked for accuracy by a second team member (TB/TD).

Data extraction of primary outcomes included psychometric outcomes of acceptability, reliability, and validity evidence. Data extracted relating to acceptability consisted of completion time and missing data reported for each measure. Missing data were extracted from reports of incomplete surveys or calculated based on the number of complete surveys included in the analysis. Reliability data extracted for scores of measures related to internal consistency, inter-rater, and test-re-test reliability coefficients. Sources of validity evidence were extracted following guidelines from the Standards for Educational and Psychological Testing [26]. Data were extracted on four sources of validity evidence: test content; response process, internal structure, and relationships to other variables. Test content refers to the relationship between the content of the items and the construct under measure, which includes analyzing the adequacy and relevance of items [26]. Validity evidence of response process involves understanding the thought processes participants use when responding to items and their consistency with the construct of focus [26]. Internal structure is defined as the degree to which test items are related to one another and coincide with the construct for which test scores are being interpreted [26]. The last source of validity evidence, relations to other variables, is the relationship of test scores to other external variables, from which it can be determined the degree to which these relationships align with the construct under measure [26].

To determine if study findings supported validity evidence based on relationships to other variables, a review of the literature was conducted and guiding tables on variable relationships were established (see Additional file 2). Data on psychometric outcomes were extracted by two independent reviewers (EB and TB/TD). All disagreements were able to be resolved by consensus between those whom extracted the data. Measures were grouped according to the number of sources of validity evidence that were reported in the study(ies) associated with each measure. In the event that multiple studies were reported for a measure, group classification was determined based on the number of sources indicated by 50% or more of the associated studies [29].

Quality assessment was not conducted due to limitations across varying and inconsistent criteria for appraising studies involving psychometric measures [27]. Instead, aligning with previous reviews [17, 29], a thorough assessment of reliability and validity evidence for scores of measures was conducted to align with the Standards for Educational and Psychological Testing [26].

### Data synthesis

A narrative synthesis of results is presented. Study statistics as they relate to setting and population are summarized. Measures are also categorized according to the number of EIDM attributes addressed. Acceptability defined as completion time and overall missing data are summarized across measures and settings. Reliability data is summarized for each measure across settings. Similar to previous psychometric systematic reviews [17, 29], measures are categorized into distinct groups based on the number of validity evidence sources reported for each measure (e.g., Group 1 = 4 sources of validity evidence). This aligns with the Standards for Psychological and Educational Testing [26] which identifies that the strength of a validity argument for scores on a measure is cumulative and contingent on the number of validity evidence sources established. As psychometric properties are based on the context in which a measure is used or tested, healthcare settings are integrated into the presentation of results.

## Results

### Review statistics

**Fig. 1:**
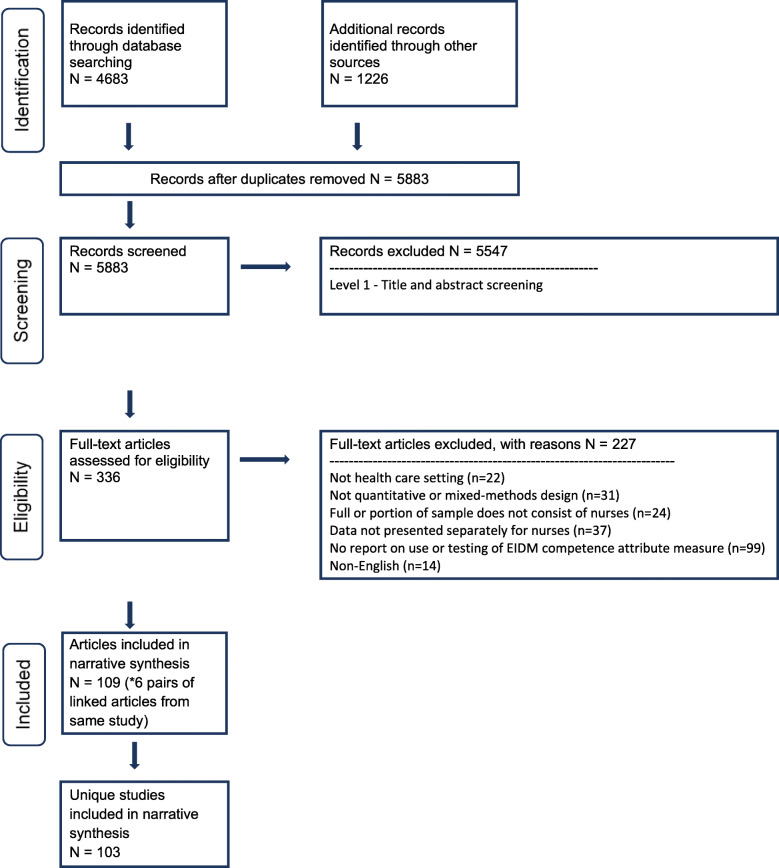
PRISMA details

In total, 5883 references were screened for eligibility at the title and abstract level. Of the 336 screened at full-text, 109 articles were included in the final review. Six pairs of articles (*n* = 12) were linked (i.e., associated with the same parent study) and the remainder of the articles were unique studies. Therefore, the review included 103 studies (see Additional file 3) and 35 unique measures (see Fig. 1 for PRISMA details).

### Study characteristics

Of the 103 studies, over half were conducted in the United States (*n* = 57; 55.3%). Twenty studies were conducted in Europe (57.1%), with 19 (54.3%) taking place in Asia. Two studies were conducted each in Africa, Australia, Canada, and one in New Zealand. Publication years spanned 2004–2017. One additional measure was identified after contacting content experts; its associated study was published in 2018.

### Settings

The 35 included measures were used or tested most often in acute care (*n* = 31 measures) followed by primary care (*n* = 9 measures). Measures were used less often in public health (*n* = 4 measures), home health (n = 4 measures), and long-term care (*n* = 1 measure). An overview of measures with identified settings is presented in Table 2.

**Table 2:** Description of EIDM competence attributes measures across setting, population (35 measures)

Name of measure (n = # of studies) [related citations]	Purpose of measure and description	Setting							Population				EIDM Competence Attributes			
		Primary care	Acute care	Public health	Home health	Long-term	Other	Not reported	RNs	APNs	RPNs/LPNs	Does not specify	Knowledge	Skills	Attitudes/Belief	Behaviours
**FOUR EIDM COMPETENCE ATTRIBUTES MEASURED (n = 3)**																
**Evidence-Based Practice Questionnaire (EBPQ)** (*n* = 36 studies) [30–66]	A 24-item self-report measure that assesses knowledge, practice, and attitudes toward evidence-based practice (EBP). Knowledge/skills (14 items) are assessed collectively using a 7-point scale (1 = poor to 7 = best). Practice is assessed with six items with a scale to determine the frequency with which that item has been completed over the past year on a 7-point scale ranging from never to frequently. Attitudes are assessed using four items also on a 7-point scale with higher scores indicating more positive attitudes towards EBP.	√	√			√	√	√	√		√	√	√	√	√	√
**School Nursing Evidence-Based Practice Questionnaire (SN-EBP)** (n = 1 study) [67]	A measure with the most applicable categories: EBP (21 items rated from 1 = strongly disagree to 5 = strongly agree); Computer use (7 items rated from 1 = avoid all together to 4 = skillfull); Information sources (10 items rated from 1 = never to 4 = all the time)			√					√				√	√	√	√
**Self-developed measure by Chiu et al. (2010)** (n = 1 study) [68]	A self-report measure to assess EBP beliefs, attitudes, knowledge, skills, behaviours and barriers. Respondents rate agreement on a 5-point Likert scale (from strongly agree to strongly disagree). EBP behaviours is defined by identifying the frequency of access to online databases.		√									√	√	√	√	√
**THREE EIDM COMPETENCE ATTRIBUTES MEASURED (n = 7)**																
**Johns Hopkins Nursing EBP Assessment Survey** (n = 1 study) [69]	An online self-report survey in which respondents were asked to rate their confidence in ability to achieve specific EBP competencies on a 6-point scale ranging from 1 = completed disagree to 6 = completely agree (I feel confident I can…).		√						√				√	√		√
**Persian translated EBP measure** (n = 1 study) [70]	A four-part self-report measure combining items from various existing measures to assess EBP knowledge, attitudes, and practice.		√						√				√	√	√	
**Self-developed measure by Yip et al.** (n = 1 study) [71]	Measure consisting of three sections with most applicable: beliefs and attitudes (5 items) rated on a Likert scale with highest score of 5 = strongly agree and knowledge and skills (9 items) rated on Likert scale with highest score of 5 = excellent.		√						√				√	√	√	
**Self-developed measure by Chew et al.** (n = 1 study) [72]	Self-report measure that assesses EBP attitude and knowledge (5 items) and resource utilization when searching for EBP (3 items).	√							√				√	√	√	
**Self-developed EBP measure by Melnyk et al. (2004)** (n = 1 study) [73]	Self-report measure with the most applicable domains: Seven items measuring knowledge, beliefs, extent of EBP on a scale from 0 (nothing, not at all) to 100 (expert, all); Nine dichotomous items about EBP implementation (e.g. Do you currently use Cochrane Database of Systematic Reviews)							√				√	√		√	√
**Modified Evidence-Based Nursing Education Questionnaire (EBEQ)** (n = 1 study) [74]	A 45-item self-report measure focused on assessing beliefs, knowledge, and self-perceived ability in EBP implementation divided into five domains: 1) knowledge 2) finding and reviewing evidence, 3) clinical practices, 4) change in clinical strategies/practices, and 5) finding and judging evidence. Response scale is a 5-point Likert scale ranging from strongly agree to strongly disagree. Higher scores are associated with positive beliefs, greater knowledge and self-perceived ability for EBP implementation.	√	√							√			√		√	√
**Quick EBP VIK (Values, Implementation, Knowledge) Survey** (n = 2 studies) [75, 76]	A 25-item self-report survey that assesses values, implementation and knowledge of EBP. Values (8 items) are assessed using a 5-point scale from 1 = strongly disagree to 5 = strongly agree. Implementation (8 items) is assessed by indicating the frequency with which an EBP activity has been performed on a 5-point scale in the last 12 months (1 = none; 2 = 1 or 2 times; 3 = 3–5 times; 4 = 6–10 times; 5 = more than 10 times). Knowledge (9 items) is assessed on a 5-point scale for each item ranging from 1 = not at all knowledgeable; 2 = minimally knowledgeable; 3 = knowledgeable; 4 = very knowledgeable; 5 = extremely knowledgeable/expert.		√						√	√		√	√		√	√
**TWO EIDM COMPETENCE ATTRIBUTES MEASURED (n = 6)**																
**Self-developed measure by Barako et al.** (n = 1 study) [77]	Self-report measure that assesses numerous domains but most applicable are attitudes toward EBP (7 items) and EBP application (1 item) with a dichotomous response of either ‘fully practice’ or ‘don’t fully practice’.		√									√			√	√
**EBP measure developed by Majid et al.** [78] (n = 2 studies) [35, 79]	A self-report measure that assesses attitude towards EBP, skills in performing EBP activities, training needs, and supporting factors and barriers in EBP implementation. Most applicable is attitudes (5-items) measured on a 5-point scale from strongly disagree to strongly agree. EBP skills are assessed (9 items) using a 5-point scale ranging from 1 = poor to 5 = excellent.		√						√			√		√	√	
**Modified Stevens EBP Readiness Inventory (ERI) (Finnish ERI)** (n = 1 study) [80]	A 25-item measure divided into two sections: 1) Consists of 20 EBP competencies which respondents rate their confidence in their ability to perform the competency (scored on a 6-point Likert scale ranging from 1 = very little confidence to 6 = a great deal of confidence in employing EBP); and 2) 15 multiple choice item to assess knowledge about major concepts in EBP. These are scored based on number of correct questions ranging from 0 to 15.		√						√				√	√		
**Self-developed measure by Gerrish et al.** (n = 1 study) [81]	A self-report measure. Many areas covered but most applicable: understanding of EBP (respondents provide open-text description of EBP understanding), 11-items for self-assessment of EBP knowledge and skills (rated on a 5-point ordinal scale (complete beginner to expert).	√	√							√			√	√		
**Knowledge and Skills in Evidence-Based Nursing (KS-EBN)** (n = 1 study) [82]	A 10-item short answer, multiple choice, and ranking measure to assess EBP nursing knowledge and skills. Each question is awarded a specific point score. Range of scores are from 0 to 12.		√									√	√	√		
**Adapted Fresno Test** (n = 1 study) [83]	A measure used to assess EBP knowledge and skills using three different pediatric nursing case scenarios. The questions relate to the case scenario and consist of both open-ended and close-ended questions. Questions are scored on a scale from 0 to 212 with higher scores indicating greater EBP knowledge and skill.		√						√				√	√		
**ONE EIDM COMPETENCE ATTRIBUTE MEASURED (n = 19)**																
**Evidence-based Practice Implementation Scale** (*n* = 35 studies) [50, 59, 63, 84–119]	An 18-item self-report measure that assesses the extent of EBP implementation. Response scale is on a 5-point frequency scale. Respondents identify the frequency (in past 8 weeks) with which they have performed that item. Scale ranges from 0 = 0 times to 4 = more than 8 times. Total score ranges from 0 to 72.	√	√	√	√		√	√	√	√	√	√				√
**Self-developed measure by Bostrom et al.** (n = 1 study) [120]	Six item measure that assesses the extent to which nurses practice EBP. Nurses respond to each item by answering the question: “To what extent do you perform the following tasks in your work as a nurse?” Each item is rated on a 4-point scale (1 = to a very low extent, 2 = to a low extent, 3 = to a high extent, 4 = to a very high extent).	√	√						√							√
**Self-developed measure by Kim et al.** (1 study) [48]	Self-report 7-item measure that assesses perceived ability to follow EBP steps. Responses are rated on a 5-point Likert scale based on Benner’s model (1 = novice, 2 = advanced beginner, 3 = competent, 4 = proficient, 5 = expert).		√						√							√
**Evidence-Based Practice Confidence Scale (EPIC)** (n = 1 study) [31, 32]	An 11-item self-report measure in which respondents rate the confidence in their ability to perform specific EBP activities/steps using an 11-point scale ranging from 0 to 100.		√						√							√
**EBP Competency Tool** *identified from content expert (n = 1 study) [10]	A self-report measure of 24 EBP competencies (items). Response scale consists of participants rating competency level on a 4-point Likert scale: 1 (not at all competent), 2 (need improvement), 3 (competent), and 4 (highly competent). Possible scores range from 0 to 96.		√				√		√	√						√
**Self-developed measure by Gerrish et al.** (n = 1 study) [121]	A self-report measure with four sections. The most applicable section is the self-assessment of nurses’ skills related to finding, reviewing, and using different evidence sources (6 items). These are ranked on a 5-point scale from 1 = complete beginner to 5 = expert.		√									√		√		
**Developing Evidence-based practice questionnaire** (n = 6 studies) [8, 122–126]	A self-report measure aimed to identify factors that influence the development of EBP. Forty-nine items are divided into five sections. Most applicable section is: self-assessment of skills in finding and reviewing evidence (eight items) which are scored on a 5-point scale from 1 = complete beginner to 5 = expert.	√	√	√	√				√	√	√	√		√		
**Information literacy tool** (n = 1 study) [59]	Nine questions to assess information searching ability.		√						√					√		
**Evidence-based Practice Beliefs Scale** (*n* = 42 studies) [50, 59, 63, 83–119, 127–133]	A 16-item self-report measure that assesses beliefs about the value of EBP and ability in implementing it. Response scale is a 5-point Likert scale to rate agreement level (1 = strongly disagree to 5 = strongly agree). Total scores can range between 16 and 80.	√	√	√	√		√	√	√	√	√	√			√	
**Modified Korean Evidence-Based Medicine questionnaire** (n = 1 study) [134]	A 23 item self-report measure that assesses participants’ perceptions (13 items), attitudes (9 items) and utilization intention (1 item) of evidence-based nursing (EBN). Participants respond on a 4-point Likert scale for perceptions and attitudes and a 3-point Likert scale for intention to use EBN to indicate their agreement with the statement (‘strongly disagree’ to ‘strongly agree’).		√						√						√	
**Evidence-Based Practice Attitudes Scale (EBPAS)** (n = 1 study) [31, 32]	An 18-item self-report scale to determine attitudes toward adopting EBP. Response for each item indicate agreement level and include: 0 = not at all; 1 = to a slight extent; 2 = to a moderate extent; 3 = to a great extent; 4 = to a very great extent.		√						√						√	
**Attitudes to Evidence-Based Practice Questionnaire** (n = 1 study) [33]	A self-report survey (originally 26-items), with 17 items used to assess attitudes/barriers toward EBP rated on a 5-point Likert scale.		√									√			√	
**Evidence-Based Nursing Attitude Questionnaire (EBNAQ)** (n = 2 studies) [135, 136]	A 15-item self-report measure that assesses attitudes towards evidence-based nursing (EBN) as it relates to the benefits of EBN, behaviours/intentions in participating in EBN, and importance level ascribed to EBN. Response scale rates the level of agreement with each item on a 5-point Likert scale ranging from 1 = strongly disagree to 5 = strongly agree.	√	√		√				√						√	
**Nurses’ Attitudes Toward EBP Scale (NATES)** (n = 1 study) [137]	An 11-item self-report measure used to assess EBP attitudes and beliefs. Response scale is a 5-point Likert scale to assess agreement (1 = strongly disagree to 5 = strongly agree). Score ranges from 5 to 55 with higher scores indicating more positive attitudes related to EBP.		√						√						√	
**Single item measure for EBP knowledge by Skela-Savic et al.** (n = 1 study) [109]	One self-report item in which respondents are asked to rate their EBP knowledge on a 5-point scale from 1 = insufficient to 5 = excellent.		√						√				√			
**Perceived EBP Knowledge Measure** (1 study) [137]	A 3-item measure that assesses a nurse’s perception of having enough knowledge, skills, and access to resources to engage in EBP. Each item is scored on an agreement scale (strongly disagree = 1 to strongly agree = 5). Total scores range from 3 to 15 with higher scores denoting increased perception of EBP knowledge.		√						√				√			
**Evidence-Based Practice Knowledge** **Assessment in Nursing (EKAN)** (n = 1 study) [45]	A 20-item multiple choice measure that assesses EBP knowledge. Total number correct is scored out of 20.		√						√				√			
**Knowledge Assessment Test (KAT)** (1 study) [66]	Objective measure assessing EBP knowledge.							√				√	√			
**Core Knowledge Questionnaire** (1 study) [62]	A 12-item multiple choice question test to measure EBP knowledge.		√						√				√			
**Total # of Measures**		**9**	**31**	**4**	**4**	**1**	**4**	**5**	**26**	**7**	**4**	**13**	**19**	**15**	**17**	**13**

Note: In some cases, measures cross multiple settings, populations, and attributes, therefore, total number of measures will not add to 35

RN: Registered Nurse

APN: Advanced Practice Nurse (e.g., Nurse Practitioner)

RPN: Registered Practice Nurse

LPN: Licensed Practical Nurse

### Population

Measures were primarily used or tested among registered nurses (*n* = 26 measures; 74.3%), followed by advanced practice nurses (*n* = 7 measures; 20%), and licensed/registered practical nurses (n = 4 measures; 11.4%). A licensure group for 13 of the measures (37.1%) was not specified. Associated population groups are presented for each measure in Table 2.

### EIDM competence attributes addressed

Measures addressed a variety of EIDM competence attributes (see Table 2). Only three measures (8.6%) assessed all four EIDM competence attributes of knowledge, skills, attitudes/beliefs, and behaviours. These included the Evidence-Based Practice Questionnaire (EBPQ) [30], the School Nursing Evidence-based Practice Questionnaire [67] and a self-developed measure by Chiu et al. [68]. Seven measures (20%) assessed three of the four EIDM competence attributes, with differing foci [69–75]. These measures all assessed knowledge, but varied on assessment of attitudes/beliefs, skills, and behaviours. Six measures (17%) addressed two EIDM competence attributes [77, 78, 80–83]. Over half of the total measures (*n* = 19; 54.3%) assessed only a single EIDM attribute. Among these single attribute measures, attitudes/beliefs were assessed the most (*n* = 6 measures) [31–33, 84, 134–137]. Overall, knowledge was the attribute addressed by most measures (n = 19), followed closely by attitudes/beliefs (*n* = 17 measures), skills (*n* = 15 measures), and behaviours (*n* = 13 measures; see Table 2).

### Psychometric outcomes

#### Acceptability

##### Missing data

Overall, missing data related to percentage of incomplete surveys were reported for 10 measures (28.6%). The range of missing data was 1.6% (EBP Beliefs Scale) - 25.6% (EBPQ) and differed across health care settings. Missing data across seven measures yielded percentages below excessive missing data limits of > 10% [138]. Reported missing data is summarized in Table 3.

**Table 3:** Acceptability findings: Missing data and completion time [related citations]

Measure	Setting					
	Acute care	Primary care	Public health	Home health	Long-term care	Not specified
**PROPORTION OF MISSING DATA (*n*** **= 10 measures)**						
EBP Beliefs Scale	10–15.9% [85, 98, 103]	Not reported	1.6% [88]	Not reported	N/A	12.8% [131]
EBP Implementation Scale	10–25.6% [85, 98, 103]	Not reported	6.3% [88]	Not reported	N/A	Not reported
Evidence-based Practice Questionnaire (EBPQ)	4.9–25% [31, 40, 45, 47]	1.8–23% [53, 55]	N/A	N/A	23% [53]	Not reported
Evidence-Based Nursing Attitude Questionnaire	7.8% [136]	Not reported	N/A	Not reported	N/A	Not reported
Evidence-Based Practice Attitudes Scale (EBPAS)	11.8% [31]	N/A	N/A	N/A	N/A	N/A
Evidence-Based Practice Confidence Scale (EPIC)	11.8% [31]	N/A	N/A	N/A	N/A	N/A
Quick EBP VIK (Values, Implementation, Knowledge) Survey	5.6% [76]	N/A	N/A	N/A	N/A	N/A
Knowledge and Skills in Evidence-Based Nursing (KS-EBN)	17.2% [82]	N/A	N/A	N/A	N/A	N/A
Evidence-Based Practice Knowledge Assessment in Nursing (EKAN)	4.9% [45]	N/A	N/A	N/A	N/A	N/A
School Nursing Evidence-Based Practice Questionnaire (SN-EBP)	N/A	N/A	5.2% [67]	N/A	N/A	N/A
**COMPLETION TIME (n = 4 measures)**						
EBP Beliefs Scale	~ 5 min [85]	Not reported	Not reported	Not reported	N/A	~ 7 min [84]
EBP Implementation Scale	~ 6 min [85]	Not reported	Not reported	Not reported	N/A	~ 8 min [84]
Evidence-based Practice Questionnaire (EBPQ)	20–25 min [34]	Not reported	N/A	N/A	Not reported	N/A
Knowledge and Skills in Evidence-Based Nursing (KS-EBN)	10–15 min [82]	N/A	N/A	N/A	N/A	N/A

##### Completion time

Data for completion time were extracted where times were explicitly stated or calculated using time to complete each item if a combined time was reported to complete multiple measures in a study. Completion time was reported for four measures, ranging from 5 (EBP Beliefs Scale) - 25 (EBPQ) minutes [34, 82, 84, 85]. A summary of reported completion time is provided in Table 3.

#### Reliability

Across measures and studies reporting reliability evidence, internal consistency was the most commonly assessed. Inter-rater and test-re-test reliability were also reported, although, for only one measure each.

##### Internal consistency

Reliability of scores, reported as Cronbach’s alpha (α), was reported for 21 measures (60%). Cronbach’s alpha values ranged widely across settings of: Acute care (0.45–0.99); primary care (0.57–0.98); public health (0.79–0.91); home health (0.63–0.87); and long-term care (0.79–0.96). Cronbach’s alphas are presented for individual measures and settings in Table 4.

**Table 4:** Reported Cronbach’s alphas for measures (*n* = 21) across settings [related citations]

Measure	Acute care	Primary care	Public health	Home health	Long-term care	Not specified
**Measures assessing*four*EIDM competence attributes**						
School Nursing Evidence-Based Practice Questionnaire	N/A	N/A	α = 0.85–0.88 1 study [67]	N/A	N/A	N/A
EBPQ	α = 0.63–0.99 28 studies [30, 32, 34–41, 43, 45–54, 56, 58–60, 62, 64, 65]	α = 0.694–0.98 5 studies [38, 50, 53, 55, 57]	N/A	N/A	α = 0.79–0.96 1 study [53]	α = 0.74–0.98 2 studies [30, 38]
**Measures assessing*three*EIDM competence attributes**						
Quick EBP Values, Implementation, Knowledge Survey (VIK)	α = 0.66–0.96 2 studies [75, 76]	N/A	N/A	N/A	N/A	N/A
Persian translated EBP measure	α = 0.89–0.93 1 study [70]	N/A	N/A	N/A	N/A	N/A
Modified Evidence-Based Nursing Education Questionnaire (EBEQ)	α = 0.57–0.91 1 study [74]	α = 0.57–0.91 1 study [74]	N/A	N/A	N/A	N/A
Self-developed measure by Yip et al.	α = 0.69–0.90 1 study [71]	N/A	N/A	N/A	N/A	N/A
**Measures assessing*two*EIDM competence attributes**						
EBP measure developed by Majid et al. [78]	α = 0.71–0.94 2 studies [35, 79]	N/A	N/A	N/A	N/A	N/A
Knowledge and Skills in Evidence-Based Nursing	α = 0.96 1 study [82]	N/A	N/A	N/A	N/A	N/A
Modified Stevens EBP Readiness Inventory (ERI) (Finnish ERI)	α = 0.98 1 study [80]	N/A	N/A	N/A	N/A	N/A
**Measures assessing*one*EIDM competence attribute**						
EBP Beliefs Scale	α = 0.776–0.95 27 studies [50, 59, 85–87, 92, 93, 95–97, 100, 102, 103, 105–107, 109, 110, 112, 113, 115–119, 127, 128, 130–132]	α = 0.88–0.92 2 studies [50, 106]	Not reported	Not reported	N/A	α = 0.90 1 study [84]
EBP Implementation Scale	α = 0.85–0.969 21 studies [50, 59, 85, 86, 92, 93, 95–97, 100, 102, 103, 105–107, 109, 110, 112, 113, 116–119]	α = 0.88–0.96 2 studies [50, 106]	Not reported	Not reported	N/A	α = 0.96 1 study [84]
DEBPQ	α = 0.77–0.913 3 studies [122, 123, 126]	α = 0.83–0.914 3 studies [122, 124, 125]	α = 0.788–0.913 3 studies [8, 122, 125]	α = 0.865 1 study [8]	N/A	N/A
Evidence-based Nursing Attitude Questionnaire	α = 0.45–0.82 1 study [136]	α = 0.63–0.86 1 study [135]	N/A	α = 0.63–0.86 1 study [135]	N/A	N/A
EBP Attitudes Scale	α = 0.771–0.794 1 study [32]	N/A	N/A	N/A	N/A	N/A
EBP Confidence Scale	α = 0.897–0.912 1 study [32]	N/A	N/A	N/A	N/A	N/A
EBP Competency Scale	α = 0.98 1 study [10]	N/A	N/A	N/A	N/A	N/A
Attitudes to Evidence-Based Practice Questionnaire	α = 0.973 1 study [33]	N/A	N/A	N/A	N/A	N/A
Modified Korean Evidence-Based Medicine Questionnaire	α = 0.85 1 study [134]	N/A	N/A	N/A	N/A	N/A
Information literacy tool	α = 0.93 1 study [59]	N/A	N/A	N/A	N/A	N/A
Perceived EBP Knowledge Measure	α = 0.80 1 study [137]	N/A	N/A	N/A	N/A	N/A
Self-developed measure by Bostrom et al.	α = 0.90 1 study [120]	α = 0.90 1 study [120]	N/A	N/A	N/A	N/A

Out of the 21 measures for which internal consistency was reported, seven measures had multiple study findings reported across unique practice settings. Reported Cronbach’s alphas were varied across and within settings for the same measure as evident by wide alpha ranges (see Table 4). Among these findings, two measures assessing EIDM attitudes with the lowest reported alphas were the Evidence-based Nursing Attitude Questionnaire (0.45) and the EBPQ (0.63 for attitude subscale) in acute care settings. The Modified Evidence-based Nursing Education Questionnaire also had a low alpha reported (0.57) in both acute and primary care settings. Regarding high range values, the EBPQ had the highest overall reported alpha (0.99) also in an acute care setting.

All 21 measures met a minimum of Cronbach’s alpha ≥0.80 [139] in at least one study instance (see Table 4).

##### Inter-rater and test-retest reliability

Test-retest reliability was assessed in only one measure, the Quick EBP Values, Implementation, Knowledge Survey [75]. Average item level test-retest coefficients ranged from below marginal to acceptable [140] at 0.51–0.70 [75].

Inter-rater reliability was reported for scores on the Knowledge and Skills in Evidence-Based Nursing measure [82]. Intraclass correlations were reported for three sections of this measure and exceeded a guideline of ≥0.80 [140].

#### Sources of validity evidence

##### Group 1: measures reporting four sources of validity evidence

Two of the 35 measures (5.7%) used/tested across three studies, were assigned to Group 1 [67, 135, 136] (see Table 5). Common across these two measures was the use of exploratory factor analysis to assess internal structure. Pertaining to validity based on relationships with other variables, this differed between the two measures. For the School Nursing Evidence Based Practice Questionnaire, the use of correlation and regression analyses supported validity evidence with significant associations between use of EBP and demographic variables (e.g., education; see Additional file 4). For the Evidence-Based Nursing Attitude Questionnaire, correlation and t-test analyses were used to establish relationships between EBP attitudes and variables related to EBP knowledge, EBP training, and education level (see Additional file 4). The measures also varied with respect to setting with the former being tested in a public health setting and the latter in acute care, primary care, and home healthcare settings.

**Table 5:** Group 1: Measures with four sources of validity evidence (*n* = 2)

Measure	Study	Setting/Licensure Group	Source of Validity Evidence			
			Content	Response process	Internal structure	Relationships to variables
**School nursing evidence-based practice questionnaire**	[67]	Public health/RNs	√	√	√	√
**Evidence-Based Nursing Attitude Questionnaire (EBNAQ)**	[135]	Home health/RNs	√	√	√	√
	[136]	Acute/RNs				√

##### Group 2: measures with three sources of validity evidence

Five measures (14%) used/tested across seven studies, were categorized in group 2 [35, 71, 75, 76, 79, 82, 137] (see Table 6). Common across all these measures was the report of validity evidence related to content and relationships to other variables. Similar to group 1, the strength of variable relationships differed, with varied use of correlational, t-test, ANOVA, and regression analyses to report significant relationships between EBP competence attributes (i.e., knowledge, implementation, skills, attitudes) and demographic, organizational variables or education interventions (see Additional file 4). Internal structure validity evidence via exploratory factor analysis was reported for three measures [71, 75, 76, 137], while response process validity evidence was reported for two measures [35, 82]. All measures were tested or used in acute care.

**Table 6:** Group 2: Measures with three sources of validity evidence (n = 5)

Measure	Study	Setting/Licensure Group	Source of Validity Evidence			
			Content	Response process	Internal structure	Relationships to variables
**Self-developed measure by Yip et al.**	[71]	Acute/RNs	√		√	√
**Quick Values, Implementation, Knowledge Survey**	[75]	Acute/APNs, “nurses in any role”	√		√	√
	[76]	Acute/RNs			√	√
**EBP measure developed by Majid et al.**	[79]	Acute/not specified	√			√
	[35]	Acute/RNs	√	√		√
**Knowledge and Skills in Evidence-Based Nursing**	[82]	Acute/not specified	√	√		√
**Perceived EBP Knowledge Measure**	[137]	Acute/RNs	√		√	√

##### Group 3: measures with two sources of validity evidence

Six measures (17%) were categorized in group 3 [10, 69, 70, 73, 80, 120] (see Table 7). Content validity evidence was commonly reported across all six measures using an expert group. Validity evidence based on relationships to other variables was reported for five of the six measures with correlational and ANOVA analyses used most often (*n* = 3 measures). Once again, regarding this source of validity evidence, significant relationships were demonstrated between EBP knowledge, attitudes, skills, and individual characteristics or organizational factors (see Additional file 4). Acute care was the most common healthcare setting (*n* = 5 measures).

**Table 7:** Group 3: Measure with two sources of validity evidence (*n* = 6)

Measure	Study	Setting/Licensure Group	Source of Validity Evidence			
			Content	Response process	Internal structure	Relationships to variables
**Modified Stevens EBP Readiness Inventory**	[80]	Acute/RNs	√			√
**Johns Hopkins Nursing EBP Assessment Survey**	[69]	Acute /RNs	√	√		
**Persian translated EBP measure**	[70]	Acute/RNs	√			√
**Self-developed EBP measure by Melnyk et al.**	[73]	Not specified	√			√
**Self-developed measure by Bostrom et al.**	[120]	Acute/RNs	√			√
**EBP Competency Tool**	[10]	Acute, not specified/RNs, APNs	√			√

##### Group 4: measures with one source of validity evidence

Over half of the measures were categorized in group 4 (*n* = 19; 54%; see Table 8). For all these measures, except one [122], validity evidence based on relationships to other variables was reported. With respect to strength of these variable relationships, t-test (*n* = 12 measures), correlational (*n* = 11 measures), and ANOVA (*n* = 8 measures) analyses were primarily conducted. Regression analyses were used less commonly (*n* = 6 measures). Similarly, as in previous groups, significant relationships between EIDM competence attributes and demographic, organizational factors, and interventions were established (see Additional file 4).

**Table 8:** Group 4: Measures with one source of validity evidence (*n* = 19)

Measure	Study	Setting/Licensure Group	Source of Validity Evidence			
			Content	Response process	Internal structure	Relationships to variables
**EBP Implementation Scale** **(35 studies)**	[92, 93]	Acute/RNs			√	√
	[84]	Not specified/Not specified	√		√	√
	[115]	Acute/Not specified				√
	[104]	Acute/RNs				√
	[105]	Acute/RNs	No supporting validity evidence			
	[101]	Home health/RNs				√
	[86]	Acute /RNs	No supporting validity evidence			
	[85]	Acute/RNs				√
	[102]	Acute/RNs				√
	[107]	Acute/RNs				√
	[106]	Acute, primary, not specified/Not specified	√			
	[117]	Acute/RNs	√	√	√	√
	[90]	Acute, primary, not specified/Not specified				√
	[96]	Acute/RNs				√
	[98]	Acute/RNs, LPNs, APNs				√
	[112]	Acute/RNs				√
	[91]	Acute/RNs				√
	[87]	Acute/RNs, APNs				√
	[113]	Acute/RNs				√
	[59]	Acute/RNs				√
	[99, 100]	Acute/RNs, APNs				√
	[109, 110]	Acute/RNs, not specified			√	√
	[118]	Acute/RNs				√
	[119]	Acute/RNs				√
	[97]	Acute/Not specified				√
	[89, 108]	Acute/RNs				√
	[88]	Public health/RNs, LPNs				√
	[116]	Acute/RNs				√
	[95]	Acute/RNs				√
	[94]	Acute/RNs				√
	[111]	Acute/RNs				√
	[50]	Acute/RNs, LPNs				√
	[63]	Acute/RNs				√
	[103]	Acute/RNs				√
	[114]	Not specified/Not specified				√
**EBP Beliefs Scale** **(42 studies)**	[92, 93]	Acute/RNs			√	√
	[84]	Not specified/Not specified	√		√	√
	[115]	Acute/Not specified				√
	[104]	Acute/RNs				√
	[105]	Acute/RNs	No supporting validity evidence			
	[101]	Home health/RNs				√
	[128]	Acute/Not specified				√
	[106]	Acute, primary, not specified/Not specified	√			√
	[130, 131]	Acute/RNs	√	√	√	√
	[117]	Acute/RNs	√	√	√	√
	[86]	Acute/RNs				√
	[85]	Acute/RNs				√
	[102]	Acute/RNs				√
	[107]	Acute/RNs				√
	[90]	Acute, not specified/Not specified	No supporting validity evidence			
	[96]	Acute/RNs				√
	[127]	Acute/RNs	No supporting validity evidence			
	[112]	Acute/RNs				√
	[98]	Not specified/RNs, APNs, LPNs				√
	[132]	Acute/RNs				√
	[83]	Acute/RNs				√
	[91]	Acute/RNs				√
	[87]	Acute/RNs, APNs				√
	[133]	Not specified/RNs, APNs				√
	[113]	Acute/RNs				√
	[59]	Acute/RNs				√
	[99, 100]	Acute/RNs, APNs				√
	[109, 110]	Acute/RNs, not specified			√	√
	[118]	Acute/RNs				√
	[119]	Acute/RNs				√
	[97]	Acute/Not specified				√
	[89, 108]	Acute/RNs				√
	[88]	Public health/RNs, LPNs				√
	[129]	Acute/RNs	No supporting validity evidence			
	[50]	Acute/RNs, LPNs				√
	[116]	Acute/RNs				√
	[95]	Acute/RNs				√
	[94]	Acute/RNs	No supporting validity evidence			
	[111]	Acute/RNs				√
	[63]	Acute/RNs				√
	[103]	Acute/RNs				√
	[114]	Not specified/not specified				√
**Evidence-Based Practice Questionnaire** **(36 studies)**	[30]	Acute, not specified/ not specified	√	√	√	√
	[49]	Acute/RNs				√
	[41]	Acute/Not specified				√
	[40]	Acute/RNs				√
	[55]	Primary/RNs				√
	[44]	Primary/Not specified				√
	[33]	Acute/Not specified				√
	[64]	Acute/RNs				√
	[62]	Acute/RNs				√
	[48]	Acute/RNs				√
	[57]	Primary/RNs			√	√
	[53]	Acute, primary, long-term care/RNs				√
	[58]	Acute/Not specified	√			√
	[39]	Acute/RNs				√
	[60]	Acute/RNs		√	√	√
	[42]	Acute/RNs				√
	[43]	Acute/RNs	No supporting validity evidence			
	[34]	Acute/RNs		√		√
	[66]	Not specified/Not specified	No supporting validity evidence			
	[38]	Acute, primary, not specified/Not specified	No supporting validity evidence			
	[35]	Acute/RNs	√			√
	[52]	Acute/Not specified			√	
	[47]	Acute/Not specified			√	√
	[54]	Acute/Not specified				√
	[56]	Acute/RNs			√	√
	[65]	Acute/RNs				√
	[31, 32]	Acute/RNs				√
	[37]	Acute/Not specified				√
	[59]	Acute/RNs				√
	[50]	Acute, primary/RNs, LPNs				√
	[45]	Acute/RNs				√
	[61]	Acute/RNs	No supporting validity evidence			
	[46]	Acute/RNs				√
	[51]	Acute/RNs	No supporting validity evidence			
	[36]	Acute/RNs				√
	[63]	Acute/RNs				√
**DEBPQ** **(6 studies)**	[122]	Acute, primary, public health/Not specified	√		√	√
	[123]	Acute/RNs	No supporting validity evidence			
	[124]	Primary/RNs, LPNs/RPNs				√
	[8]	Public health, home health/Not specified	No supporting validity evidence			
	[125]	Primary, public health, home health/RNs, APNs				√
	[126]	Acute/RNs				√
**Modified Evidence-Based Nursing Education Questionnaire (1 study)**	[74]	Acute, primary/APNs				√
**Self-developed measure by Barako et al. (1 study)**	[77]	Acute/Not specified				√
**Self-developed measure by Gerrish et al. (1 study)**	[81]	Acute, primary/APNs				√
**Adapted Fresno Test** **(1 study)**	[83]	Acute/RNs				√
**Self-developed measure by Kim et al.** **(1 study)**	[48]	Acute/RNs				√
**EBP confidence scale** **(1 study)**	[31, 32]	Acute/RNs				√
**Information literacy tool** **(1 study)**	[59]	Acute/RNs				√
**Modified Korean EBM questionnaire** **(1 study)**	[134]	Acute/RNs				√
**EBP Attitudes Scale** **(1 study)**	[31, 32]	Acute/RNs				√
**Attitudes to Evidence-Based Practice Questionnaire** **(1 study)**	[33]	Acute/Not specified				√
**Nurses’ Attitudes Toward EBP Scale** **(1 study)**	[137]	Acute/RNs				√
**Single item measure for EBP knowledge** **(1 study)**	[109]	Acute/RNs				√
**EBP Knowledge Assessment in Nursing** **(1 study)**	[45]	Acute/RNs				√
**Knowledge Assessment Test** **(1 study)**	[66]	Not specified/Not specified				√
**Core Knowledge Questionnaire** **(1 study)**	[62]	Acute/RNs				√

##### Group 5: measures with no sources of validity evidence

No sources of validity evidence were found for three measures [68, 72, 121].

See Additional file 4 for detailed information on validity evidence sources for each measure with supporting evidence.

#### Validity evidence and settings

Most of the measures (*n* = 29; 83%) reported validity evidence in the context of acute care settings. For nine measures, validity evidence was reported across multiple settings. For three of these measures (EBP Implementation Scale, EBP-Beliefs Scale, EBPQ), multiple sources of validity (> 1) were more often reported in acute care settings compared to other practice settings where only one source of validity evidence was commonly found. In contrast, one measure (Evidence-based Nursing Attitude Questionnaire) had four sources of validity evidence established in primary and home care settings but not in acute care. While, the same number of validity sources were established for five additional measures (Developing Evidence-based Practice Questionnaire, modified Evidence-based Nursing Education Questionnaire, two unnamed self-developed measures, EBP Competency Tool) across varied healthcare settings.

## Discussion

This review furthers our understanding about measures assessing EIDM competence attributes in nursing practice. Findings highlight limitations in the existing literature with respect to use or testing of measures across practice settings, the diversity in EIDM competence attributes addressed, and variability in the process and outcomes of psychometric assessment of existing measures.

### Settings

This review contributes new insight about settings in which EIDM measures have been used or tested that previous systematic reviews have not addressed. This review reveals a concentration on use or testing of EIDM measures in acute care (*n* = 31 measures; 89%) compared to other healthcare contexts (primary care, home health, public health, long-term care). This imbalance was also observed in an integrative review of 37 studies exploring the knowledge, skills, attitudes and capabilities of nurses in EIDM [9] where the majority of studies (*n* = 27) were conducted in hospitals, with fewer conducted in primary, community, and home healthcare, and none in long-term care. While there is a large body of evidence to support understanding of the psychometric rigor of EIDM measures in acute care, more attention and investment is required for this type of understanding in community-based and long-term care contexts. Given current trends and priorities in healthcare such as the reorientation toward home care [141], attention toward disease prevention and management, and health promotion [142], and a large aging population with growing projections of residence in long-term care facilities [143], it is of great importance to assess EIDM competence across all nursing practice settings to ensure efficient, safe, and patient-centred care.

### EIDM competence attributes addressed

This review also adds to the current literature on nursing EIDM competence measures using a broader conceptualization of competence. That is, the measures reviewed focus on four competence attributes of knowledge, skills, attitudes/beliefs, and behaviours. In comparison, Leung et al. [14] assess measures focused on three attributes; knowledge, attitudes and skills. In our current review, three measures [30, 67, 68] addressed all four EIDM attributes (e.g., knowledge, skills, attitudes/beliefs, behaviours). Measures that address all four attributes are of critical importance given the inextricable link between knowledge, skills, attitudes and behaviours to comprise professional competence [144–146]. Professional competence cannot sufficiently develop if each attribute was to support it independently [147]. Knowledge without skill, or the ability to use knowledge, renders knowledge useless [148]. Similarly, performing a skill without understanding the reasoning behind it contributes to unsafe and incompetent practice [148, 149]. And lastly, possessing knowledge and skill without the experience of their application in the real world is insufficient to qualify as competent [150].

However, despite these measures addressing all four competence attributes, based.

on their response scales used, they do not conceptually reflect an assessment of competence, defined as *quality* of ability or performance to an expected standard [150], but rather, focus on mere completion or frequency of completing tasks. Quality versus frequency of behaviours are distinct concepts and have been measured separately in nursing performance studies [19, 151]. The provision of a high standard of patient care includes nursing competence assessment, which is a critical component of quality improvement processes, workforce development and management [19, 152]. This conceptual limitation of existing EIDM measures highlights a need for a measure that aligns with the conceptual understanding of competence as an interrelation between knowledge, skills, attitudes/beliefs, behaviours [144] and quality of ability [150].

### Psychometric outcomes

#### Acceptability

Despite acceptability, measured as amount of missing data and completion times, being identified as a critical aspect of psychometric assessment [153], discussion of acceptability among included primary studies was lacking compared to an emphasis on reliability or validity. In this review, only 10 measures (28.6%) reported missing data. In addition, only four measures (11%) reported completion times. This limited discussion of acceptability is reinforced by findings from a systematic review of research utilization measures by Squires et al. [29] in which no studies reported acceptability data. As well, acceptability was not mentioned or discussed in systematic reviews of EIDM measures for nurses, midwives [14], medical practitioners [17] and allied health professionals [23]. Discussions about acceptability have typically been explored in the context of patient-reported outcome measures [153]. These discussions also hold relevance for measures with healthcare professionals as end users [154, 155]. Time and ease of completing a measure are important considerations for nurses or managers who work in fast-paced clinical settings, which can influence their decision to integrate these measures into their practice.

### Reliability

Findings from the current review determine gaps in reliability testing of measures in addition to variable findings across EIDM measures and healthcare contexts.

Internal consistency reported as Cronbach’s alpha was the most commonly assessed type of reliability in this review. This appears to be a trend similarly found among EIDM related psychometric reviews [14, 23]. Cronbach’s alpha is a commonly used statistic in psychometric research perhaps due to its ease of calculation as it can be computed with a one-time administration [156]. While Nunnally [157] identifies that the “coefficient alpha provides a good estimate of reliability in most cases” (p. 211), there are important considerations with its use. One consideration is that interpretation of Cronbach’s alpha requires an understanding that it must be re-evaluated in each new setting or population a measure is used in [158]. In the current review, many of the studies associated with frequently used measures (EBP-Implementation Scale, EBP Beliefs Scale) did not re-evaluate internal consistency when using the measure in a new or different setting from where it was originally tested. This was evident from unreported data in multiple studies associated with the same measure but taking place across various healthcare settings. Other reviews have reported similar findings, whereby measures have not been re-assessed in new contexts, and have reported either no data or only original internal consistency findings [13, 16]. The importance of re-assessing and interpreting this reliability statistic in new contexts is further underscored by current review findings in which Cronbach’s alphas varied widely across unique practice settings for the same measure.

Moreover, there were heterogenous findings among studies taking place in the same type of setting for the same measure. Within each setting, there were instances in which the same measure would result in varying Cronbach’s alphas with range values falling both below and above minimum guidelines of ≥0.80 [139]. For example, Mooney [86] reported a Cronbach’s alpha of 0.776 for the EBP Beliefs Scale when used in an acute care setting, while Underhill et al. [87] reported α = 0.95 with the same measure also used in acute care practice. Variability in internal consistency findings has been reported in other systematic reviews as well [16, 23], perhaps due to the use of measures in diverse populations, settings, and countries. This further indicates the effect of nuanced populations within similar practice settings on internal consistency findings.

In addition, lower alphas were typically reported for EIDM attitude scales, such as for the self-developed measure by Yip et al. [71] (α = 0.69), the EBNAQ [135, 136] (α = 0.45) and the EBPQ (α = 0.63) [30]. A possible explanation of these low alphas may be related to the low number of items on an EIDM attitude subscale compared to other EIDM competence attributes. As Streiner [25] indicates, the length of a scale highly impacts internal consistency, and as such, reliability could plausibly be improved through the addition of conceptually robust items. Further to this, in a literature review of the uses of the EBPQ [159], authors note that low alpha scores for the attitude subscale were consistently reported, due to repeated item deletions or modifications, calling for further refinement of EIDM attitudes items.

Overall, there was a lack of reliability assessment as 40% of measures did not report reliability. This occurred for both newly developed and established measures. The lack of reliability testing has also been identified in existing reviews assessing EIDM measures among allied healthcare professionals [13, 16, 23] as early as 2010. The ongoing lack of attention to reliability assessment highlights a need for more rigorous and standardized reliability testing not only in the original development of measures but also in its subsequent use in different healthcare environments.

### Validity

Findings pertaining to validity evidence when compared to existing literature show both alignment and contrast with respect to how validity evidence was assessed, and the number and type of validity sources established across measures.

As noted, psychometric assessment of the current review was based on the contemporary understanding that the strength of a validity argument is dependent on the accumulation of different validity evidence sources [26]. In this review, only one source of validity evidence was reported for over half of the measures (*n* = 19; 54%). Very few measures were reported with four (*n* = 2 measures) or three (*n* = 5 measures) validity evidence sources established. Employing a similar approach to validity evidence assessment, Squires et al. [29] reported similar findings in their review of research utilization measures: the majority of measures were categorized under level three of their hierarchy (i.e., one source of validity evidence); no measures were reported as having all four sources of validity evidence; and six measures were associated with three sources of validity evidence.

Since existing reviews did not present validity evidence in the context of practice settings, this presents challenges with comparison of results. However, this review presents some insight on contextualizing validity evidence. In the current review, much of the validity evidence was presented in the context of an acute care setting, and in particular, for three measures most widely used (EBP Implementation Scale, EBP Beliefs Scale, EBPQ), more sources of validity evidence were established by the original developers in acute care practice. Similar to reliability findings, this brings to light a critical gap in nursing research with respect to the use of measures after their original development, and lack of validity evidence assessment in different settings and populations. This demonstrates a call to action for nursing researchers that a consistent level of rigor must be applied to comprehensively re-assess sources of validity evidence for a measure when using it in a new practice setting. This strengthens a cumulative body of validity evidence to support continued use of a measure in varied nursing contexts.

Compared to the current review, previous EIDM psychometric systematic reviews [13, 14, 16] included traditional assessments of content, criterion, and construct validity and demonstrated variable findings. Buchanan et al. [13] reported no findings related to validity for 18 measures and failure to re-test validity by authors when original measures were used in a new study setting. Glegg and Holsti [16] only provided a description of validity data and did not perform an assessment through scoring or ranking of this evidence. While, Leung et al. [14] used their self-developed Psychometric Grading Framework [160] to assess validity of instruments in their review. These authors determined that most of the studies reported measures as having ‘weak’ or ‘very weak’ validity according to their matrix scoring, with only three studies reporting the tested measures as having adequate validity [14].

Included studies in this review also limited validity assessment to sources based on test content and relationships to other variables, focusing on construct validity. This appears to be a consistent theme reported across existing reviews as well [14, 23]. A new contribution from this review is an in-depth understanding about the strength of validity evidence based on relationships to other variables. Data extracted on the statistical analyses associated with this source of validity evidence showed relationships established primarily through correlational, t-test or ANOVA analyses. In less instances, regression analyses were used to demonstrate strong relationships, highlighting a need in psychometric evaluation of tools to validate more robust relationships between variables.

Findings from the current review and existing literature highlight limitations in assessing validity evidence and the psychometric rigor of existing EIDM measures. Variability in testing and results of validity evidence creates challenges and confusion for end users in research or nursing practice who look to this body of literature to determine appropriate and robust EIDM measures. Scholarly support for the use of a comprehensive and contemporary approach in psychometric development of tools can help to standardize assessments and produce findings representative of a unified understanding of validity evidence.

### Considerations for tool selection in nursing practice or research

This systematic review can serve as a helpful resource for nursing administrators, frontline staff, or researchers who are interested in using a measure to assess a specific EIDM competence attribute. In selecting measures for nursing practice or research, the specific population and setting in which measures have been previously used or tested, in addition to specific EIDM competence attributes they address, all serve as important considerations. As well, looking to the acceptability of measures, taking into account tool completion time given demands of busy clinical environments and if high rates of missing data > 10% are present [138], are also critical factors to consider for decision-making. Acceptable reliability of a measure should also be given weight in tool selection (α ≥ 0.80) [139], in addition to determining how comprehensively all four sources of validity evidence (content, internal structure, response process, relationships to other variables) have been established for a given measure [26].

### Limitations

A limitation of this review relates to the absence of quality assessments of included primary studies. Given that traditional quality assessment was not conducted, this may influence the confidence in study findings and thus results are to be interpreted with caution. However, among tools previously used to assess quality of psychometric studies, several limitations exist [27]. These include the development of quality assessment tools for use only with patient reported outcome measures [14], using a lowest score ranking method providing an imbalance in the overall quality score [161], and a lack of validity and reliability testing [27]. Most importantly, existing quality assessment tools employ a traditional approach of assessing construct, content, and criterion validity, rather than a contemporary perspective of viewing validity evidence as a unified concept [26], as used to guide the current review. Given this, to align with other reviews using a similar contemporary approach [17, 29] assessment was focused on the categorization of measures according to the number of sources of validity evidence established for scores in related studies. A second limitation pertains to the exclusion of non-English literature as there were 14 articles identified from full-text screening requiring translation for seven languages, which were excluded from the review. Given the large number of studies included in the final review, it is unlikely that the small number of non-English studies would have a critical impact on results. A third limitation is that with the use of a classification system for assessing validity evidence, the number of studies for a particular measure could influence the strength of the validity argument [29]. A measure which has one or a small number of studies may appear to have strong validity evidence [29] as compared to those measures with more cited studies. Implications of this are most relevant for more established measures, in that more sources of validity evidence may have in fact been established, but only in a small amount of studies, which may not be reflected in its final categorization. However, the advantage of using this synthesis process is that it highlights the types of validity evidence that require further testing for a particular measure [29].

## Conclusions

There is a diverse collection of measures that assess EIDM competence attributes of knowledge, skills, attitudes/beliefs, and/or behaviours in nurses. Among these measures is a concentration on the assessment of single EIDM competence attributes. Review findings determined that three measures addressed all four EIDM attributes, although with some conceptual limitations, highlighting a need for a tool that comprehensively assesses EIDM competence. More rigorous and consistent psychometric testing is also needed for EIDM measures overall, but particularly in community-based and long-term care settings in which the data is limited. A contemporary approach to psychometric assessment of EIDM measures in the future may also provide more robust and comprehensive evidence of their psychometric rigor.

## Supplementary information

**Additional file 1.** Electronic database search strategy Identifies key words used for each primary database searched.

**Additional file 2.** Theoretical and empirical literature to guide data analysis of sources of validity evidence Tables used to determine supporting validity evidence for data extracted.

**Additional file 3.** Included studies Description of included studies.

**Additional file 4.** Sources of validity evidence for each measure Identifies supporting data for each source of validity evidence established.

## Data Availability

The data included in this review was retrieved from published studies and also through supplementary documents provided by study authors upon request as necessary.

## Abbreviations

EIDM: Evidence-informed decision-making
EBP: Evidence-based practice
EBPQ: Evidence-based practice questionnaire
