# Supplementary material for: Measures of evidence-informed decision-making competence attributes: a psychometric systematic review
Source: BMC Nurs. 2020 May 27;19:44. doi: 10.1186/s12912-020-00436-8 (PMC7254762; doi:10.1186/s12912-020-00436-8)
Supplement: Supplementary file 1 — Additional file 1. Electronic database search strategy Identifies key words used for each primary database searched. [file 12912_2020_436_MOESM1_ESM.docx]

**Additional file 1: Electronic database search strategy**

**Ovid MEDLINE**

1. Nurse*.mp.
2. exp Nurses/
3. 1 or 2
4. evidence based practice.mp.
5. evidence informed decision making.mp.
6. evidence based nursing.mp.
7. Evidence-Based Practice/
8. Evidence-Based Nursing/
9. 4 or 5 or 6 or 7 or 8
10. measurement*.mp.
11. assessment*.mp.
12. psychometric*.mp.
13. reliability*.mp.
14. validity*.mp.
15. questionnaire*.mp.
16. survey*.mp.
17. scale*.mp.
18. tool*.mp.
19. Surveys and Questionnaires/
20. Psychometrics/
21. 10 or 11 or 12 or 13 or 14 or 15 or 16 or 17 or 18 or 19 or 20
22. Knowledge.mp.
23. “professional knowledge”.mp.
24. “nursing knowledge”.mp.
25. Understanding.mp.
26. Comprehension.mp.
27. Knowledge/
28. Comprehension/
29. 22 or 23 or 24 or 25 or 26 or 27 or 28
30. Skill*.mp.
31. “competency assessment”.mp.
32. 30 or 31
33. Behavio?r*.mp.
34. “Professional behaviour”*.mp.
35. “professional competence”.mp.
36. Competence.mp.
37. Clinical decision making.mp.
38. Professional Competence/
39. Clinical Competence/
40. Clinical Decision-Making/
41. 33 or 34 or 35 or 36 or 37 or 38 or 39 or 40
42. Attitude*.mp.
43. Belief*.mp.
44. “Professional value”.mp.
45. Nurse attitude.mp.
46. Attitude/
47. "Attitude of Health Personnel"/
48. 42 or 43 or 44 or 45 or 46 or 47 or 48
49. 3 and 9 and 21
50. 3 and 9 and 21 and 29 or 32 or 41 or 49

**EMBASE**

1. Nurse*.mp.
2. exp nurse/
3. 1 or 2
4. evidence based practice.mp.
5. evidence informed decision making.mp.
6. evidence based nursing.mp.
7. evidence based nursing/
8. evidence based practice/
9. 4 or 5 or 6 or 7 or 8
10. measurement*.mp.
11. assessment*.mp.
12. psychometric*.mp.
13. reliability*.mp.
14. validity*.mp.
15. questionnaire*.mp.
16. survey*
17. scale*
18. tool*
19. measurement/
20. questionnaire/
21. reliability/
22. rating scale/
23. outcome assessment/
24. questionnaire/
25. 10 or 11 or 12 or 13 or 14 or 15 or 16 or 17 or 18 or 19 or 20 or 21 or 22 or 23 or 24
26. Knowledge.mp.
27. Understanding.mp.
28. Comprehension.mp.
29. “professional knowledge”.mp.
30. “nursing knowledge”.mp.
31. professional knowledge/
32. knowledge base/
33. nursing knowledge/
34. 26 or 27 or 28 or 29 or 30 or 31 or 32 or 33
35. Skill*.mp.
36. “competency assessment”.mp.
37. skill/
38. 35 or 36 or 37
39. Behavio?r*.mp.
40. “Professional behaviour”*.mp.
41. “professional competence”.mp.
42. Competence.mp.
43. Clinical decision making.mp.
44. clinical decision making/
45. behavior/
46. professional competence/
47. 39 or 40 or 41 or 42 or 43 or 44 or 45 or 46
48. Attitude*.mp.
49. Belief*.mp.
50. Nurse attitude.mp.
51. “Professional value”*.mp.
52. nurse attitude/
53. attitude assessment/
54. attitude/
55. 48 or 49 or 50 or 51 or 52 or 53 or 54
56. 3 and 9 and 25
57. 3 and 9 and 25 and 34 or 38 or 47 or 55

**CINAHL**

1. Nurse*.mp.
2. 1 or 2
3. evidence based practice.mp.
4. evidence informed decision making.mp.
5. evidence based nursing.mp.
6. MH "Professional Practice, Evidence-Based"
7. MH "Nursing Practice, Evidence-Based"
8. 3 or 4 or 5 or 6 or 7
9. measurement*.mp.
10. assessment*.mp.
11. psychometric*.mp.
12. reliability*.mp.
13. validity*.mp.
14. questionnaire*.mp.
15. survey*
16. scale*
17. tool*
18. MH "Psychometrics"
19. MH "Measurement Issues and Assessments"
20. 9 or 10 or 11 or 12 or 13 or 14 or 15 or 16 or 17 or 18 or 19
21. Knowledge.mp.
22. Understanding.mp.
23. Comprehension.mp.
24. “nursing knowledge”.mp.
25. “professional knowledge”.mp.
26. MH "Knowledge"
27. MH "Nursing Knowledge"
28. MH "Professional Knowledge"
29. 21 or 22 or 23 or 24 or 25 or 26 or 27 or 28
30. Skill*.mp.
31. “competency assessment”.mp.
32. MH "Competency Assessment"
33. 30 or 31 or 32
34. Behavio?r*.mp.
35. “Professional behaviour”*.mp.
36. “professional competence”.mp.
37. Competence.mp.
38. Clinical decision making.mp.
39. MH "Professional Competence"
40. 34 or 35 or 36 or 37 or 38 or 39
41. Attitude*.mp.
42. Belief*.mp.
43. Nurse attitude.mp.
44. “Professional value”*.mp.
45. 41 or 42 or 43 or 44
46. 2 and 8 and 20
47. 2 and 8 and 20 and 20 or 29 or 33 or 40

**ERIC and MathSciNet**

Nurse* AND

“Evidence based practice” OR “Evidence informed decision making” OR “Evidence based nursing” AND

Measurement* OR assessment* OR tool* OR Psychometric* OR Reliability* OR Validity* OR Questionnaire* OR survey* OR scale* AND

Knowledge OR understanding OR comprehension OR professional knowledge OR nursing knowledge OR Skill* OR competency assessment OR Behavio?r* OR Professional behaviour* OR professional competence OR competence OR clinical decision making OR Attitude* OR Belief* OR Professional value* OR nurse attitude

**HaPI**

1. Nurse*.mp.
2. evidence based practice.mp.
3. evidence informed decision making.mp.
4. evidence based nursing.mp.
5. 2 or 3 or 4 or 5
6. measurement*.mp.
7. assessment*.mp.
8. psychometric*.mp.
9. reliability*.mp.
10. validity*.mp.
11. questionnaire*.mp.
12. survey*.mp.
13. scale*.mp.
14. tool*.mp.
15. 6 or 7 or 8 or 9 or 10 or 11 or 12 or 13 or 14
16. 1 and 5 and 15
