# Supplementary material for: Measures of evidence-informed decision-making competence attributes: a psychometric systematic review
Source: BMC Nurs. 2020 May 27;19:44. doi: 10.1186/s12912-020-00436-8 (PMC7254762; doi:10.1186/s12912-020-00436-8)
Supplement: Supplementary file 2 — Additional file 2. Theoretical and empirical literature to guide data analysis of sources of validity evidence Tables used to determine supporting validity evidence for data extracted. [file 12912_2020_436_MOESM2_ESM.docx]

**Additional file 2: Theoretical and empirical literature to guide data analysis of sources of validity evidence**

**Theoretical literature**

| **Theory, Model, Framework** | **Citation** | **Description** |
| --- | --- | --- |
| Stetler Model | Stetler, C. (2001). Updating the Stetler model of research utilization to facilitate evidence-based practice. *Nursing Outlook, 49*(6), 272-279.  Ciliska, D. et al. (2011). Models to guide implementation of evidence-based practice. In *Evidence-Based Practice in Nursing and Healthcare: A Guide to Best Practice* (Melnyk B. & Fineout-Overholt, E., eds). Wolters Kluwer, Philadelphia, PA, pp. 241-275. | Key assumptions:   - An organization may or may not be involved in a professional’s use of research:   - If the individual has the appropriate competencies and updates knowledge base, use of research findings can occur at the individual level. Organizations may also support research use through education, policies, procedures. - Utilization may be instrumental, conceptual, and/or symbolic:   - Research can be used in many ways (e.g., change personal way of thinking, influence an action of change, used to change others’ thinking and/or behaviour). - Decision-making can be influenced by other types of evidence/non-research information together with research evidence:   - This may include theoretical, experiential, local program data or consensus information. - Factors (internal and external) impact a person’s or group’s view and use of evidence:   - These factors may include professional’s characteristics, surrounding environment - Research and evaluation is not considered absolute information:   - Research may not be applicable to all patients in every situation. There must be some understanding about specific patient preferences/needs, and other variations in the application of research. - Lack of knowledge and skills related to EIDM/research utilization (RU) may hinder their use:   - Specific EIDM/RU knowledge and skills are required for their implementation, in addition to critical thinking skills   Steps (emphasis on individual):   1. Preparation: identify priority need and begin search for evidence 2. Validation: critique and summarize evidence 3. Comparative Evaluation and Decision-Making: decide about evidence to be used in response to need 4. Translation and Application: create evidence-based action plan and implement it 5. Evaluation: identify if EIDM goals were achieved |
| PARIHS (Promoting Action on Research Implementation in Health Services) Framework | Rycroft-Malone, J. (2004). The PARIHS framework – a framework for guiding the implementation of evidence-based practice. *Journal of Nursing Care Quality, 19*(4), 297-304. | Emphasis on organizational use:  Successful EIDM implementation is a function of the following inter-connected elements:   1. *Evidence:* credible knowledge from different sources (e.g. research, clinical experience, patient experience, local data/information) 2. *Context:* innovation adoption is influenced by organizational culture (e.g. decentralized decision-making, relationships between frontline staff and managers, facilitative management styles), leadership (e.g. transformational leadership), and evaluation practices (e.g. evaluation frameworks that rely on multiple sources of evidence to show effectiveness) 3. *Facilitation*: individuals who use their knowledge and skills to help other staff, teams and the organization to make the EIDM change |
| ARCC (Advancing Research and Clinical Practice Through Close Collaboration) Model | Melnyk, B., Fineout-Overholt, E., Gallagher-Ford, L., & Stillwell, S. (2011). Sustaining evidence-based practice through organizational policies and an innovative model. *American Journal of Nursing, 111*(9), 57-60. | Four key assumptions:   - EIDM barriers to and facilitators at the individual and health care systems level - EIDM barriers must be mitigated and facilitators put in place for EIDM implementation - EIDM beliefs, values, and confidence in ability to implement EIDM serve as facilitators to EIDM implementation and therefore should be strengthened - An EIDM mentoring culture helps to advance and sustain EIDM among   professionals and in health care systems  Steps (emphasis on organizational use):   1. Assess organizational culture and readiness for EIDM implementation 2. Identify organization strengths and barriers to EIDM 3. Identify EIDM mentors for frontline staff 4. Implementation of evidence in practice 5. Evaluate outcomes |

**Empirical literature**

| **Citation** | **Sample** | **Dependent Variable** | **Other Variables** | **Description of Relationship** |
| --- | --- | --- | --- | --- |
| **Individual factors** | | | |  |
| Lizarondo, L., Grimmer-Somers, K., & Kumar, S. (2011). A systematic review of the individual determinants of research evidence use in allied health. *Journal of Multidisciplinary Healthcare, 4,* 261-272. | - Allied healthcare professionals | - Perceptions, attitudes, knowledge, and self-report use of EIDM or research | - Level of education (Working toward or having a graduate degree, advanced certification) - Involvement in research EIDM-related activities (e.g. engaged in research activities at work, taken a research or EIDM course) | - Positive effect: associated with higher perceptions, attitudes, knowledge, use of EIDM or positive perceived importance of research - Positive effect: associated with increased self-report of EIDM, positive perceptions and attitudes toward research |
| Squires, J., Estabrooks, C., Gustavsson, P., & Wallin, L. (2011). Individual determinants of research utilization by nurses: a systematic review. *Implementation Science, 6*, 1-20. | - Nurses | - Research utilization (RU) | - Attitude toward research - Attendance at conference and/or in-services - Level of education (when a nurse possesses a graduate degree compared to bachelor’s degree or diploma) - Advanced practice and leadership role - Working on specialty wards (e.g. critical care) - Job satisfaction | - High moderate positive effect - Positive association; unable to compute magnitude effect - Positive effect - Positive effect (those in advance current roles had higher levels of RU) - Positive effect (those in speciality wards had higher RU levels than those in general wards) - Positive effect |
| Eizenberg, M. (2010). Implementation of evidence based nursing practice: nurses’ personal and professional factors? *Journal of Advanced Nursing, 67*(1): 33-42. | - Nurses | - Self-report of evidence based nursing practice behaviours | - Role (those in managerial role compared to non-managerial role) - Education (those with a degree compared to those without a degree) - Six predicting factors of evidence-based nursing practice behaviours:  1. Belief in skills of finding, reading, and applying various research sources 2. Sources of knowledge based on reading professional literature 3. Education (higher levels of education) 4. System support in reading and searching professional literature 5. Sources of knowledge based on experience/intuition 6. Sources of knowledge based on colleagues and system procedures | - Positive effect - Positive effect - Positive effect (#1 - #5) - Negative effect (the more colleagues and procedures were depended on for knowledge sources, the lower probability of self-report EIDM behaviours) |
| **Interventions** | | | | |
| Häggman-Laitila, A., Mattila, L., & Melender, H. (2016). Educational interventions on evidence-based nursing in clinical practice: A systematic review with qualitative analysis. *Nurse Education Today, 43*, 50-59. | - Nurses | - EIDM beliefs, attitudes, skills, implementation | - Interventions: lecture/educational classes/interactive sessions/computer-based learning modules, EIDM toolkit, EIDM mentor, environmental prompts | - Positive effect |
| Young, T., Rohwer, A., Volmink, J., & Clarke, M. (2014). What Are the Effects of Teaching Evidence-Based Health Care (EBHC)? Overview of Systematic Reviews. *PLoS ONE 9*(1), e86706 | - Various healthcare professionals | - EIDM skill related to question formulation - Critical appraisal knowledge, skill, reading habit, attitude - EIDM knowledge, skills, behaviours - EIDM knowledge and research reading behaviour | - Multifaceted interventions (e.g. lectures, tutorials, e-learning, journal clubs, etc.) - Critical appraisal teaching/seminars - EIDM workshops - Journal clubs | - Positive effect on EIDM skill (specifically clinical question formulation/problem identification) - Positive effect - Positive effect - Positive effect |
| **Organizational Factors** | | | | |
| Williams, B., Perillo, S., & Brown, T. (2014). What are the factors of organizational culture in health care settings that act as barriers to the implementation of evidence-based practice? A scoping review. *Nurse Education Today, 35*, e34-e41. | - Various healthcare professionals | - EIDM implementation | Factors influencing EIDM implementation:   - Workload – most frequently reported barrier due to emphasis on patient-based tasks. Influenced by belief that EIDM is an ‘add-on’ to existing workload and lack of protected time for ‘EIDM activities’. - Other staff/management not supportive of EIDM - Culture in which EIDM is not highly valued - Lack of resources – lack of easily accessible resources, lack of library/staff - Lack of authority to change practice – hierarchies prevents staff from feeling that their ideas are valued and they can contribute to EIDM change - Workplace culture resistant to change – reliance on rigid, outdated protocols; mentality of ‘this is how it’s always been done’ | - Barriers *Scoping review |
| Solomons, N., & Spross, J. (2011). Evidence-based practice barriers and facilitators from a continuous quality improvement perspective: an integrative review. *Journal of Nursing Management,* *19*, 109–120. | - Nurses | - EIDM implementation | Factors influencing implementation organized according to different dimensions:  *Strategic*   - Lack of time/demanding workload - Lack of infrastructure to support research activities - Lack of administrative support for EIDM changes - Nursing presence on hospital wide committees - Chief nursing officer leadership in EIDM   *Cultural*   - Resistance to changing practice from manager/co-worker - Lack of authority of change practice - Employment of Health Science librarian - Institution of EIDM champions (to cultivate staff ownership of EIDM)   *Technical*   - Lack of initial and ongoing training related to EIDM knowledge/skills - Lack of accessible resources (e.g. online databases) - Hands-on training to address EIDM knowledge/skill deficiencies   *Structural*   - Lack of awareness of research - Distilling and dissemination of research to employees of organization - Journal clubs | *Integrative review   - Barrier - Barrier - Barrier - Facilitator - Facilitator - Barrier - Barrier - Facilitator - Facilitator - Barrier - Barrier - Facilitator - Barrier - Facilitators - Facilitator |
