# Supplementary material for: Measures of evidence-informed decision-making competence attributes: a psychometric systematic review
Source: BMC Nurs. 2020 May 27;19:44. doi: 10.1186/s12912-020-00436-8 (PMC7254762; doi:10.1186/s12912-020-00436-8)
Supplement: Supplementary file 3 — Additional file 3. Included studies Description of included studies. [file 12912_2020_436_MOESM3_ESM.docx]

**Additional file 3: Included studies**

| **Authors** | **Citation title** | | | **Journal** | **Year** | **Study design** | **Country** | **Sample size** | **Funding description** | | |
| --- | --- | --- | --- | --- | --- | --- | --- | --- | --- | --- | --- |
| B. Skela-Savic, S. Hvalic-Touzery, K. Pesjak [109]  B. Skela-Savic, K. Pesjak, B. Lobe [110]  *linked articles | Professional values and competencies as explanatory factors for the use of evidence-based practice in nursing  Evidence-based practice among nurses in Slovenian Hospitals: a national survey | | | Journal of Advanced Nursing  International Nursing Review | 2017  2016 | Cross-sectional  Cross-sectional  Correlational | Slovenia | 780  534 | Yes. Describe | Ministry of Higher Education, Science and Technology of the Republic of Slovenia | |
| R. Lovelace, M. Noonen, J. F. Bena, A. S. Tang, M. Angie, R. Cwynar, R. Field, J. Rosenberger, D. Ross, D. Walker, N. M. Albert [50] | Value of, Attitudes Toward, and Implementation of Evidence-Based Practices Based on Use of Self-Study Learning Modules | | | Journal of Continuing Education in Nursing | 2017 | Other: please describe : single group post-test only | Ohio and Florida | 1,033 (14.8%) | Yes. Describe | Cleveland Clinic Health System, Nursing Institute | |
| H. Saunders, K. R. Stevens, K. Vehvilainen-Julkunen [80] | Nurses' readiness for evidence-based practice at Finnish university hospitals: a national survey | | | Journal of Advanced Nursing | 2016 | Cross-sectional | Finland | 943 | Yes. Describe | Finnish Nurses’ Education Foundation, the Finnish Nurses’ Association, the Saastamoinen Foundation and the Finnish Work Environment Fund, e University of Eastern Finland, Faculty of Health Sciences, Department of Nursing Sciences | |
| E. Shafiei, A. Baratimarnani, S. Goharinezhad, R. Kalhor, M. Azmal [58] | Nurses' perceptions of evidence-based practice: a quantitative study at a teaching hospital in Iran | | | Medical Journal of the Islamic Republic of Iran | 2014 | Cross-sectional | Iran | 195 | Yes. Describe | Research affairs of Bushehr University of Medical Sciences | |
| A. A. Ammouri, A. A. Raddaha, P. Dsouza, R. Geethakrishnan, J. A. Noronha, A. A. Obeidat, L. Shakman [39] | Evidence-Based Practice: Knowledge, attitudes, practice and perceived barriers among nurses in Oman | | | Sultan Qaboos University Medical Journal | 2014 | Cross-sectional  Correlational | Muscat Oman | 414 | No. Not reported | | |
| K. Stokke, N. R. Olsen, B. Espehaug, M. W. Nortvedt [112] | Evidence based practice beliefs and implementation among nurses: a cross-sectional study | | | BMC Nursing | 2014 | Cross-sectional | Norway | 185 | No. Not reported | | |
| A. Seyyedrasooli, V. Zamanzadeh, L. Valizadeh, F. Tadaion [70] | Individual Potentials Related to Evidence-Based Nursing among Nurses in Teaching Hospitals Affiliated to Tabriz University of Medical Sciences, Tabriz, Iran | | | Journal of Caring Sciences | 2012 | Correlational | Tabriz, Iran | 600 | No. Not reported | | |
| J. I. Shin, E. Lee [126] | The Influence of Social Capital on Nurse-Perceived Evidence-Based Practice Implementation in South Korea | | | Journal of Nursing Scholarship | 2017 | Cross-sectional  Correlational | South Korea | 432 | No. Not reported | | |
| H. Verloo, M. Desmedt, D. Morin [116] | Beliefs and implementation of evidence-based practice among nurses and allied healthcare providers in the Valais hospital, Switzerland | | | Journal of Evaluation in Clinical Practice | 2017 | Cross-sectional | Valais, Switzerland | 329 | No. Not reported | | |
| M. G. Harper, L. Gallagher-Ford, J. I. Warren, M. Troseth, L. T. Sinnott, B. K. Thomas [95] | Evidence-Based Practice and U.S. Healthcare Outcomes: Findings From a National Survey With Nursing Professional Development Practitioners | | | Journal for Nurses in Professional Development | 2017 | Cross-sectional  Correlational | United States (43 States and District of Columbia) | 253 | Yes. Describe | Elsevier Clinical Solutions | |
| A. Hagedorn Wonder, A. M. McNelis, D. Spurlock, P. M. Ironside, S. Lancaster, C. R. Davis, M. Gainey, N. Verwers [45] | Comparison of Nurses' Self-Reported and Objectively Measured Evidence-Based Practice Knowledge | | | Journal of Continuing Education in Nursing | 2017 | Cross-sectional  Correlational | United States | 151 | Yes. Describe | Ethel Clarke Fellowship at Indiana University | |
| J. Y. Sim, K. S. Jang, N. Y. Kim [59] | Effects of Education Programs on Evidence-Based Practice Implementation for Clinical Nurses | | | Journal of Continuing Education in Nursing | 2016 | Quasi-experimental (e.g. pre-post-test) | South Korea | 63 | No. Not reported | | |
| S. C. Kim, L. Ecoff, C. E. Brown, A. M. Gallo, J. F. Stichler, J. E. Davidson [99]  S. C. Kim, J. F. Stichler, L. Ecoff, C. E. Brown, A. M. Gallo, J. E. Davidson [100]  *articles linked | Benefits of a Regional Evidence-Based Practice Fellowship Program: A Test of the ARCC Model  Predictors of Evidence-Based Practice Implementation, Job Satisfaction, and Group Cohesion Among Regional Fellowship Program Participants | | | Worldviews on Evidence-Based Nursing | 2017  2016 | Quasi-experimental (e.g. pre-post-test)  Correlational | San Diego, California | 120  175 | No. Not reported | | |
| K. M. Bissett, M. Cvach, K. M. White [69] | Improving Competence and Confidence With Evidence-Based Practice Among Nurses: Outcomes of a Quality Improvement Project | | | Journal for Nurses in Professional Development | 2016 | Quasi-experimental (e.g. pre-post-test) | United States | 17 | No. Not reported | | |
| Y. J. Son, Y. Song, S. Y. Park, J. I. Kim [60] | A psychometric evaluation of the Korean version of the evidence-based practice questionnaire for nurses | | | Contemporary Nurse | 2014 | Cross-sectional | Korea | 801 | Yes. Describe | National Research Foundation of Korea (NRF) grant funded by the Korean government | |
| R. P. Pereira, A. C. Guerra, M. J. Cardoso, A. T. dos Santos, C. de Figueiredo Mdo, A. C. Carneiro [52] | Validation of the Portuguese version of the Evidence-Based Practice Questionnaire | | | Revista Latino-Americana de Enfermagem | 2015 | Cross-sectional | Portugal | 358 | No. Not reported | | |
| S. Hellier, T. Cline [74] | Factors that affect nurse practitioners' implementation of evidence-based practice | | | Journal of the American Association of Nurse Practitioners | 2016 | Cross-sectional  Correlational | United States | 480 | No. Not reported | | |
| L. Connor, F. Paul, M. McCabe, S. Ziniel [75] | Measuring Nurses' Value, Implementation, and Knowledge of Evidence-Based Practice: Further Psychometric Testing of the Quick-EBP-VIK Survey | | | Worldviews on Evidence-Based Nursing | 2017 | Other: please describe : identified as 'descriptive study' | United States | 382 | No. Not reported | | |
| M. A. Friesen, J. M. Brady, R. Milligan, P. Christensen [94] | Findings From a Pilot Study: Bringing Evidence-Based Practice to the Bedside | | | Worldviews on Evidence-Based Nursing | 2017 | Other: please describe : mixed methods design | United States | 57 | Yes. Describe | Inova Seed Grant | |
| J. W. Park, J. A. Ahn, M. M. Park [134] | Factors influencing evidence-based nursing utilization intention in Korean practice nurses | | | International Journal of Nursing Practice | 2015 | Cross-sectional | Korea | 420 | Yes. Describe | Ajou University College of Nursing, Suwon, Republic of Korea | |
| D. C. Stavor, J. Zedreck-Gonzalez, R. L. Hoffmann [61] | Improving the Use of Evidence-Based Practice and Research Utilization Through the Identification of Barriers to Implementation in a Critical Access Hospital | | | Journal of Nursing Administration | 2017 | Other: please describe: descriptive, quality improvement study | US | 51 | No. Not reported | | |
| J. Farokhzadian, R. Khajouei, L. Ahmadian [79] | Evaluating factors associated with implementing evidence-based practice in nursing | | | Journal of Evaluation in Clinical Practice | 2015 | Cross-sectional | Iran | 182 | No. Not reported | | |
| J. I. Hwang, H. A. Park [47] | Relationships between evidence-based practice, quality improvement and clinical error experience of nurses in Korean hospitals | | | Journal of Nursing Management | 2015 | Cross-sectional | Korea | 443 | Yes. Describe | National Research Foundation of Korea – Grant funded by the Korean Government (NRF-2009-327-H00039). | |
| J. I. Warren, M. McLaughlin, J. Bardsley, J. Eich, C. A. Esche, L. Kropkowski, S. Risch [118] | The Strengths and Challenges of Implementing EBP in Healthcare Systems | | | Worldviews on Evidence-Based Nursing | 2016 | Cross-sectional | United States | 1608 | No. Not reported | | |
| J. I. Warren, K. L. Montgomery, E. Friedmann [119] | Three-Year Pre-Post Analysis of EBP Integration in a Magnet-Designated Community Hospital | | | Worldviews on Evidence-Based Nursing | 2016 | Other: please describe : Retrospective | United States | 275 | No. Not reported | | |
| J. R. Duffy, S. Culp, K. Sand-Jecklin, L. Stroupe, N. Lucke-Wold [32]  J. R. Duffy, S. Culp, C. Yarberry, L. Stroupe, K. Sand-Jecklin, A. Sparks Coburn [31]  *articles linked | Nurses' Research Capacity, Use of Evidence, and Research Productivity in Acute Care: Year 1 Findings From a Partnership Study  Nurses' research capacity and use of evidence in acute care: baseline findings from a partnership study | | | Journal of Nursing Administration | 2016  2015 | Quasi-experimental (e.g. pre-post-test)  Correlational | United States | 67  75 | No. Not reported  Yes. Describe | | West Virginia University Nursing Research Investment Fund |
| C. Phillips [54] | Relationships between duration of practice, educational level, and perception of barriers to implement evidence-based practice among critical care nurses | | | International Journal of Evidence-Based Healthcare | 2015 | Correlational | United States | 60 | No. Not reported | | |
| A. J. Ramos-Morcillo, S. Fernandez-Salazar, M. Ruzafa-Martinez, R. Del-Pino-Casado [56] | Effectiveness of a Brief, Basic Evidence-Based Practice Course for Clinical Nurses | | | Worldviews on Evidence-Based Nursing | 2015 | Quasi-experimental (e.g. pre-post-test) | Spain | 109 | Yes. Describe | Nursing Council of Jaen´ (Reference Number: 01-2014 CEJ). | |
| L. M. Baird, T. Miller [125] | Factors influencing evidence-based practice for community nurses | | | British Journal of Community Nursing | 2015 | Not reported | Canada | 68 | No. Not reported | | |
| L. H. Eaton, A. R. Meins, P. H. Mitchell, J. Voss, A. Z. Doorenbos [91] | Evidence-based practice beliefs and behaviors of nurses providing cancer pain management: a mixed-methods approach | | | Oncology Nursing Forum | 2015 | Cross-sectional  Other: mixed methods | United States | 40 | No. Not reported | | |
| K. M. Williamson, M. Almaskari, Z. Lester, D. Maguire [65] | Utilization of evidence-based practice knowledge, attitude, and skill of clinical nurses in the planning of professional development programming | | | Journal for Nurses in Professional Development | 2015 | Other: descriptive mixed methods | United States | 151 | No. Not reported | | |
| M. Underhill, K. Roper, M. L. Siefert, J. Boucher, D. Berry [87] | Evidence-based practice beliefs and implementation before and after an initiative to promote evidence-based nursing in an ambulatory oncology setting | | | Worldviews on Evidence-Based Nursing | 2015 | Quasi-experimental (e.g. pre-post-test) | US | 112 (T1) and 113 (T2) | No. Not reported | | |
| M. O. Gu, Y. Ha, J. Kim [82] | Development and validation of an instrument to assess knowledge and skills of evidence-based nursing | | | Journal of Clinical Nursing | 2015 | Other: psychometric study | South Korea | 48 nurses from the EBP group and 43 from the non-EBP group participated in the study. | Yes. Describe | the Fund of Research Promotion Program, Gyeongsang National University, 2011 | |
| M. A. Perez-Campos, I. Sanchez-Garcia, P. L. Pancorbo-Hidalgo [53] | Knowledge, Attitude and Use of Evidence-Based Practice among nurses active on the Internet | | | Investigacion y Educacion en Enfermeria | 2014 | Correlational  Other: observational | | 314 | No. Not reported | | |
| L. Kaplan, E. Zeller, D. Damitio, S. Culbert, K. B. Bayley [98] | Improving the culture of evidence-based practice at a Magnet hospital | | | Journal for Nurses in Professional Development | 2014 | Quasi-experimental (e.g. pre-post-test) | | 207 | No. Not reported | | |
| A. Sese-Abad, J. De Pedro-Gomez, M. Bennasar-Veny, P. Sastre, J. C. Fernandez-Dominguez, J. M. Morales-Asencio [57] | A multisample model validation of the evidence-based practice questionnaire | | | Research in Nursing & Health | 2014 | Cross-sectional  Other: psychometric | Spain | 1,673 | Yes. Describe | ” financed by the Health Research Fund (PI 09/90512. Health Ministry) | |
| H. S. Thorsteinsson, H. Sveinsdottir [132] | Readiness for and predictors of evidence-based practice of acute-care nurses: a cross-sectional postal survey | | | Scandinavian Journal of Caring Sciences | 2014 | Cross-sectional | Iceland | 343 | Yes. Describe | partially funded with grants from the University Hospital Research fund and the Icelandic Nurses’ Association Research | |
| B. M. Toole, J. F. Stichler, L. Ecoff, L. Kath [62] | Promoting nurses' knowledge in evidence-based practice: do educational methods matter? | | | Journal for Nurses in Professional Development | 2013 | Experimental (e.g. RCT) | US | 596 | No. Not reported | | |
| M. J. Dropkin [90] | Review of "The State of Evidence-based Practice in US Nurses" | | | ORL - Head & Neck Nursing | 2013 | Other: descriptive survey | US | 1015 | No. Not reported | | |
| A. M. Bostrom, A. Rudman, A. Ehrenberg, J. P. Gustavsson, L. Wallin [120] | Factors associated with evidence-based practice among registered nurses in Sweden: a national cross-sectional study | | | BMC Health Services Research | 2013 | Cross-sectional | Sweden | 1256 | Yes. Describe | AFA insurance | |
| C. White-Williams, P. Patrician, P. Fazeli, M. A. Degges, S. Graham, M. Andison, A. Shedlarski, L. Harris, K. A. McCaleb [64] | Use, knowledge, and attitudes toward evidence-based practice among nursing staff | | | Journal of Continuing Education in Nursing | 2013 | Correlational | US | 593 | No. Not reported | | |
| M. J. Linton, M. A. Prasun [33] | Evidence-based practice: collaboration between education and nursing management | | | Journal of Nursing Management | 2013 | Other: descriptive survey | US | 286 | No. Not reported  Funding was not provided for this research project. | | |
| H. S. Thorsteinsson [131]  H. S. Thorsteinsson [130]  *articles linked | Icelandic nurses' beliefs, skills, and resources associated with evidence-based practice and related factors: a national survey  Translation and validation of two evidence-based nursing practice instruments | | | Worldviews on Evidence-Based Nursing  International Nursing Review | 2013  2012 | Other: descriptive survey  Other: psychometric study | Iceland | 540 | Yes. Describe | The Landspitali University Hospital Research Fund and the Icelandic Nurses Association Research Fund | |
| S. Hauck, R. P. Winsett, J. Kuric [96] | Leadership facilitation strategies to establish evidence-based practice in an acute care hospital | | | Journal of Advanced Nursing | 2013 | Other: prospective descriptive comparative | US | 475 | No. Not reported | | |
| D. Hagler, M. Z. Mays, S. B. Stillwell, B. Kastenbaum, R. Brooks, E. Fineout-Overholt, K. M. Williamson, J. Jirsak [128] | Preparing clinical preceptors to support nursing students in evidence-based practice | | | Journal of Continuing Education in Nursing | 2012 | Not reported | US | 160 | Yes. Describe | Funding for the project was provided through the Division of Nursing, Bureau of Health Professions, Health Resources and Services Administration, Department of Health and Human Services grant #D11HP09753, Leveraging Educational Technology for EvidenceBased Practice | |
| B. M. Melnyk, E. Fineout-Overholt, L. Gallagher-Ford, L. Kaplan [106] | The state of evidence-based practice in US nurses: critical implications for nurse leaders and educators | | | Journal of Nursing Administration | 2012 | Other: descriptive survey | US | 876 | No. Not reported | | |
| S. Gonzalez-Torrente, J. Pericas-Beltran, M. Bennasar-Veny, R. Adrover-Barcelo, J. M. Morales-Asencio, J. De Pedro-Gomez [23] | Perception of evidence-based practice and the professional environment of primary health care nurses in the Spanish context: a cross-sectional study | | | BMC Health Services Research | 2012 | Cross-sectional | Spain | 377 | Yes. Describe | the Health Research Fund (PI 09/90512. Health Ministry) | |
| S. C. Wang, L. L. Lee, W. H. Wang, H. C. Sung, H. K. Chang, M. Y. Hsu, S. C. Chang, C. H. Tai [117] | Psychometric testing of the Chinese evidence-based practice scales | | | Journal of Advanced Nursing | 2012 | Other: psychometric instrument design | Taiwan | 676 | Yes. Describe | This project was financially supported by the Buddhist Tzu Chi General Hospital (Project number: TCRD 98-48). | |
| M. Ruzafa-Martinez, L. Lopez-Iborra, M. Madrigal-Torres [135] | Attitude towards Evidence-Based Nursing Questionnaire: development and psychometric testing in Spanish community nurses | | | Journal of Evaluation in Clinical Practice | 2011 | Other: psychometric study | Spain | 395 | Yes. Describe | The study was supported by the EMCA Programme from the Government of Murcia Health Affairs. Financial programme for the development of research related to the Quality of Health Services 2007 (registry number: EMCA 07/01). | |
| K. Gerrish, L. Guillaume, M. Kirshbaum, A. McDonnell, A. Tod, M. Nolan [81] | Factors influencing the contribution of advanced practice nurses to promoting evidence-based practice among front-line nurses: findings from a cross-sectional survey | | | Journal of Advanced Nursing | 2011 | Cross-sectional | England | 855 | Yes. Describe | This research was funded by the Policy Research Programme of the Department of Health, England. Grant number 0160072. | |
| R. F. Levin, E. Fineout-Overholt, B. M. Melnyk, M. Barnes, M. J. Vetter [101] | Fostering evidence-based practice to improve nurse and cost outcomes in a community health setting: a pilot test of the advancing research and clinical practice through close collaboration model | | | Nursing Administration Quarterly | 2011 | Experimental (e.g. RCT) | US | 46 | Yes. Describe | Hugoton Foundation. | |
| P. Prior, J. Wilkinson, S. Neville [55] | Practice nurse use of evidence in clinical practice: a descriptive survey | | | Nursing Praxis in New Zealand | 2010 | Other: descriptive survey | Auckland, New Zealand | 55 | No. Not reported | | |
| C. E. Brown, L. Ecoff, S. C. Kim, M. A. Wickline, B. Rose, K. Klimpel, D. Glaser [40] | Multi-institutional study of barriers to research utilisation and evidence-based practice among hospital nurses | | | Journal of Clinical Nursing | 2010 | Cross-sectional | US | 1301 | Yes. Describe | Sigma Theta Tau local chapter, Gamma Gamma | |
| Y. W. Chiu, Y. H. Weng, H. L. Lo, Y. H. Shih, C. C. Hsu, K. N. Kuo [68] | Impact of a nationwide outreach program on the diffusion of evidence-based practice in Taiwan | | | International Journal for Quality in Health Care | 2010 | Cross-sectional | Taiwan | 2069 | Yes. Describe | National Health Research Institutes, Taiwan | |
| B. M. Melnyk, T. Bullock, J. McGrath, D. Jacobson, S. Kelly, L. Baba [105] | Translating the evidence-based NICU COPE program for parents of premature infants into clinical practice: impact on nurses' evidence-based practice and lessons learned | | | Journal of Perinatal & Neonatal Nursing | 2010 | Other: single cohort before and after | Southwest United States | 81 | Yes. Describe | Phoenix Children’s Hospital Competitive Grant Award | |
| J. Mills, J. Field, R. Cant [124] | The place of knowledge and evidence in the context of Australian general practice nursing | | | Worldviews on Evidence-Based Nursing | 2009 | Cross-sectional | Australia | 590 | Yes. Describe | An Australian Government National Health and Medical Research Council Primary Health Care Fellowship | |
| C. E. Brown, M. A. Wickline, L. Ecoff, D. Glaser [41] | Nursing practice, knowledge, attitudes and perceived barriers to evidence-based practice at an academic medical center | | | Journal of Advanced Nursing | 2009 | Cross-sectional | United States | 458 | Yes. Describe | Sigma Theta Tau International, Gamma Gamma chapter and from University of California, San Diego | |
| B. M. Melnyk, E. Fineout-Overholt, M. Z. Mays [84] | The evidence-based practice beliefs and implementation scales: psychometric properties of two new instruments | | | Worldviews on Evidence-Based Nursing | 2008 | Cross-sectional | United States | 394 | No. Not reported | | |
| L. Thiel, Y. Ghosh [137] | Determining registered nurses' readiness for evidence-based practice | | | Worldviews on Evidence-Based Nursing | 2008 | Cross-sectional | United States | 121 | No. Not reported | | |
| G. Varnell, B. Haas, G. Duke, K. Hudson [115] | Effect of an educational intervention on attitudes toward and implementation of evidence-based practice | | | Worldviews on Evidence-Based Nursing | 2008 | Quasi-experimental (e.g. pre-post-test) | United States | 49 | No. Not reported | | |
| M. L. Koehn, K. Lehman [49] | Nurses' perceptions of evidence-based nursing practice | | | Journal of Advanced Nursing | 2008 | Cross-sectional | United States | 422 | No. Not reported | | |
| K. Gerrish, P. Ashworth, A. Lacey, J. Bailey [123] | Developing evidence-based practice: experiences of senior and junior clinical nurses | | | Journal of Advanced Nursing | 2008 | Cross-sectional | England | 598 | Yes. Describe | identified that funding was obtained but no description provided | |
| K. Gerrish, P. Ashworth, A. Lacey, J. Bailey, J. Cooke, S. Kendall, E. McNeilly [122] | Factors influencing the development of evidence-based practice: a research tool | | | Journal of Advanced Nursing | 2007 | Cross-sectional | England | 1287 | Yes. Describe | identifies funding obtained but does not specify granting organization | |
| D. Upton, P. Upton [30] | Development of an evidence-based practice questionnaire for nurses | | | Journal of Advanced Nursing | 2006 | Cross-sectional | Wales | 751 | Yes. Describe | identifies funding obtained but does not specify granting organization | |
| B. M. Melnyk, E. Fineout-Overholt, N. Fischbeck Feinstein, H. Li, L. Small, L. Wilcox, R. Kraus [73] | Nurses' perceived knowledge, beliefs, skills, and needs regarding evidence-based practice: implications for accelerating the paradigm shift | | | Worldviews on Evidence-Based Nursing | 2004 | Cross-sectional | United States | 160 | Yes. Describe | Agency for healthcare research and quality | |
| K. Gerrish, J. Clayton [121] | Promoting evidence-based practice: an organizational approach | | | Journal of Nursing Management | 2004 | Cross-sectional | England | 330 | No. Not reported | | |
| M. O. A. Hasheesh, M. E. AbuRuz [46] | Knowledge, attitude and practice of nurses towards evidence-based practice at Al-Medina, KSA | | | Jordan Medical Journal | 2017 | Cross-sectional  Correlational | Saudi Arabia | 303 | No. Not reported | | |
| J. B. Carlone, O. Igbirieh [42] | Measuring attitudes and knowledge of evidence-based practice in the qatar nursing workforce: A quantitative cross-sectional analysis of barriers to empowerment | | | Avicenna | 2014 | Cross-sectional | Qatar | 400 | No. Not reported | | |
| Lora Moore [51] | Effectiveness of an Online Educational Module in Improving Evidence-Based Practice Skills of Practicing Registered Nurses | | | Worldviews on Evidence-Based Nursing | 2017 | Quasi-experimental (e.g. pre-post-test) | | 77 post-tests were used in analysis | No. Not reported | | |
| Kim Son Chae, Jaynelle F. Stichler, Laurie Ecoff, Ana-Mari Gallo, Judy E. Davidson [111] | Six-Month Follow-up of a Regional Evidence-based Practice Fellowship Program | | | Journal of Nursing Administration | 2017 | Quasi-experimental (e.g. pre-post-test) | United States | 66 | No. Not reported | | |
| Mohannad Eid AbuRuz, Haneen Abu Hayeah, Ghadeer Al-Dweik, Hekmat Yousef Al-Akash [36] | Knowledge, Attitudes, and Practice about Evidence-Based Practice: A Jordanian Study | | | Health Science Journal | 2017 | Cross-sectional | Amman Jordan | 500 | Yes. Describe | Applied Science Private University, Amman, Jordan | |
| Kang Younhee, Yang In-Suk [97] | Evidence-based nursing practice and its correlates among Korean nurses | | | Applied Nursing Research | 2016 | Cross-sectional,  Correlational | Korea | 392 | No. Not reported | | |
| Leonie Rose Bovino, Anne Aquila, Richard Feinn [89]  L. Rose Bovino, A. M. Aquila, S. Bartos, T. McCurry, C. E. Cunningham, T. Lane, N. Rogucki, J. DosSantos, D. Moody, K. Mealia-Ospina, J. Pust-Marcone, J. Quiles [108]  *linked articles | Evidence-Based Nursing Practice in a Contemporary Acute Care Hospital Setting  A Cross-sectional Study on Evidence-Based Nursing Practice in the Contemporary Hospital Setting: Implications for Nurses in Professional Development | | | Nursing Research  Journal for Nurses in Professional Development | 2016  2017 | Cross-sectional | United States | 402 | No. Not reported | | |
| Donna Agnew [37] | A Survey of Nurses' Knowledge, Attitude and Skills with Evidence-Based Practice in the Practice Setting | | | Nursing Research | 2016 | Other: please describe : descriptive comparative self-report survey | United States | 259 | No. Not reported | | |
| Debra Hain  Mary Haras [133] | Continuing Nursing Education. Changing Nephrology Nurses' Beliefs about the Value of Evidence-Based Practice and Their Ability to Implement in Clinical Practice | | | Nephrology Nursing Journal | 2015 | Other: please describe : descriptive | United States | 52 (12 included in data analysis in April 2014 session and 40 included in data analysis in October 2014) | No. Not reported | | |
| Nicole Allen, Barbara G. Lubejko, Julie Thompson, Barbara S. Turner [38] | Evaluation of a Web Course to Increase Evidence-Based Practice Knowledge Among Nurses | | | Clinical Journal of Oncology Nursing | 2015 | Other: pre-post retrospective | United States | 225 | No. Not reported | | |
| Aliyu Adamu, Joanne Rachel Naidoo [35] | EXPLORING THE PERCEPTIONS OF REGISTERED NURSES TOWARDS EVIDENCE-BASED PRACTICE INA SELECTED GENERAL HOSPITAL IN NIGERIA | | | Africa Journal of Nursing & Midwifery | 2015 | Other: quantitative descriptive | Nigeria | 133 | No. Not reported | | |
| Jed Duff, Margaret Butler, Menna Davies, Robyn Williams, Jannelle Carlile [43] | Perioperative nurses' knowledge, practice, attitude, and perceived barriers to evidence use: A multisite, cross-sectional survey | | | ACORN: The Journal of Perioperative Nursing in Australia | 2014 | Cross-sectional | New South Wales. | 493 | Yes. Describe | NSW Operating Theatre Association | |
| Susanne Tacaraya Fehr [34] | Examining the Relationship Between Nursing Informatics Competency and Evidence-Based Practice Competency Among Acute Care Nurses | | | | 2014 | Cross-sectional | United States, Northern Virginia | 197 | No. Not reported | | |
| Natasha Laibhen-Parkes [83] | Web-Based evidence based practice educational intervention to improve EBP competence among BSN-prepared pediatric bedside nurses: A mixed methods pilot study | | | | 2014 | Other: mixed methods | Southeastern United States | 29 | Yes. Describe | Jonas Nurse Leader’s Scholarship, Georgia Baptist College of Nursing, Nurse Faculty Load Program | |
| Kate Gerrish, Jo Cooke [8] | Factors influencing evidence-based practice among community nurses | | | Journal of Community Nursing | 2013 | Not reported | South Yorkshire | 337 | No. Not reported | | |
| Son Chae Kim, Caroline E. Brown, Laurie Ecoff, Judy E. Davidson, Ana-Maria Gallo, Kathy Klimpel, Mary A. Wickline [48] | Regional Evidence-Based Practice Fellowship Program: Impact on Evidence-Based Practice Implementation and Barriers | | | Clinical Nursing Research | 2013 | Quasi-experimental (e.g. pre-post-test) | San Diego, USA | 142 | Yes. Describe | Consortium for Nursing Excellence, San Diego, CA, USA and Alumni Faculty Grant 12-1804 from Point Loma Nazarene University, San Diego, CA, USA. | |
| Dawna L. Cato [127] | The relationship between a nurse residency program and evidence-based practice knowledge of the incumbent nurse across a multihospital system: a quantitative correlational design | | | | 2013 | Correlational | United State | 44 | No. Not reported | | |
| Talaso D. Barako, Margaret Chege, Sabina Wakasiaka, Lilian Omondi [77] | Factors influencing application of evidence-based practice among nurses | | | African Journal of Midwifery & Women's Health | 2012 | Cross-sectional | Nairobi, Kenya | 156 | No. Not reported | | |
| Steve Mooney [86] | The Effect of Education on Evidence-Based Practice and Nurses' Beliefs/Attitudes Toward and Intent to use Evidence-Based Practice | | | | 2012 | Quasi-experimental (e.g. pre-post-test) | United States | 5 | No. Not reported | | |
| Lynn Gallagher-Ford [85] | The influence of nurse leaders and nurse educators on registered nurses' evidence-based practice | | | | 2012 | Correlational | United States | 269 | No. Not reported | | |
| Susan Hall Lynch [102] | Nurses' Beliefs About and Use of Evidence-Based Practice | | | | 2012 | Cross-sectional | United States | 326 | No. Not reported | | |
| Yvette M. Pryse [107] | Using evidence based practice: the relationship between work environment, nursing leadership, and nurses at the bedside | | | | 2012 | Other: descriptive | US | 422 | No. Not reported | | |
| K. G. Mariano, L. M. Caley, L. Eschberger, A. Woloszyn, P. Volker, M. S. Leonard, Y. Tung [104] | Building evidence-based practice with staff nurses through mentoring | | | Journal of Neonatal Nursing | 2009 | Quasi-experimental (e.g. pre-post-test) | United States | 20 | Yes. Describe | Sigma Theta Tau Gamma Kappa Chapter for financial support. | |
| N. A. Estrada [93]  N. Estrada [92]  *articles linked | Learning organizations and evidence-based practice by RNs | | | | 2007 | Other: descriptive non-experimental | United States | 592 | No. Not reported | | |
|  | Exploring perceptions of a learning organization by RNs and relationship to EBP beliefs and implementation in the acute care setting | | Worldviews on Evidence-Based Nursing | | 2009 | Cross-sectional |  | 594 |  |  |  |
| Susan Lynn Adams [67] | Understanding the variables that influence translation of evidence-based practice into school nursing | | | | 2007 | Cross-sectional | United States | 386 | No. Not reported | | |
| M. L. Chew, K. H. Sim, Y. F. Sim, C. C. Yan [72] | Attitudes, skills and knowledge of primary healthcare nurses on the use of evidence-based nursing (EBN) and barriers influencing the use of EBN in the primary healthcare setting | | | Annals of the Academy of Medicine Singapore | 2015 | Cross-sectional | Singapore | 219 | No. Not reported | | |
| H. T. Xie, Z. Y. Zhou, C. Q. Xu, S. Ong, A. Govindasamy [66] | Nurses' attitudes towards research and evidence-based practice | | | Annals of the Academy of Medicine Singapore | 2015 | Other: descriptive survey | SIngapore | 68 | No. Not reported | | |
| W. K. Yip, S. Z. Mordiffi, M. S. Majid, E. K. N. Ang [71] | Nurses' perspective towards evidence-based practice: A descriptive study | | | Annals of the Academy of Medicine Singapore | 2010 | Cross-sectional | Singapore | 1144 | Yes. Describe | Funding provided by the Ministry of Health Nursing Research Committee. | |
| Lai Ping Atalanta Wan [63] | Educational Intervention Effects on Nurses' Perceived Ability to Implement Evidence-Based Practice | | | | 2017 | Quasi-experimental (e.g. pre-post-test) | US | 39 | No. Not reported | | |
| Mohammed Almaskari [136] | Omani Staff Nurses' and Nurse Leaders' Attitudes Toward and Perceptions of Barriers and Facilitators to the Implementation of Evidence-Based Practice | | | | 2017 | Other: please describe : exploratory descriptive comparative research design | Oman | 260 | Yes. Describe | Ministry of Higher Education, the Ministry of Health, The Directorate of Education and Training, and Ibra Nursing Institute | |
| Linda Connor [76] | Pediatric Nurses' Knowledge, Values, and Implementation of Evidence-Based Practice and Use of Two Patient Safety Goals | | | | 2017 | Other: please describe : descriptive quantitative research design using survey methodology | US | 190 | No. Not reported | | |
| Irene Macyk [103] | Staff Nurse Engagement, Decisional Involvement, Staff Nurse Participation in Shared Governance Councils and the Relationship to Evidence Based Practice Belief and Implementation | | | | 2017 | Correlational | US | 156 | No. Not reported | | |
| Michelle Baxley [88] | School nurse's implementation of evidence-based practice: A mixed method study | | | | 2016 | Other: mixed method | US | 59 | No. Not reported | | |
| Sherri L. Smith-Keys [129] | Education and Mentoring of Staff Nurses in Evidence Based Practice | | | | 2016 | Other: Descriptive study | US | 7 | No. Not reported | | |
| Carolyn Sweetapple [113] | Change Adoption Willingness: Development of a measure of willingness to adopt evidence-based practice in registered nurses | | | | 2015 | Other: survey development | US | 356 | No. Not reported | | |
| Temple, B.  Sawatzky-Dickson, D.  Pereira, A.  Martin, D.  McMillan, D.  Cepanec, D.  Goodwin, B.  Harwood, R [114] | Improving Nurses' Beliefs and Use of Evidence in their Practice, Nursing Education and Health Care Organizations | | | | 2014 | Cross-sectional | Canada | 257 | No. Not reported | | |
| Melnyk, Bernadette Mazurek  Gallagher‐Ford, Lynn  Zellefrow, Cindy  Tucker, Sharon  Thomas, Bindu  Sinnott, Loraine T.  Tan, Alai [10]  ***Identified from contact with content expert Dr. Melnyk** | The First U.S. Study on Nurses’ Evidence‐Based Practice Competencies Indicates Major Deficits That Threaten Healthcare Quality, Safety, and Patient Outcomes | Worldviews on Evidence-Based Nursing | | | 2018 | Cross-sectional | US | 2,344 | No. Not reported | | |
