# Supplementary material for: Measures of evidence-informed decision-making competence attributes: a psychometric systematic review
Source: BMC Nurs. 2020 May 27;19:44. doi: 10.1186/s12912-020-00436-8 (PMC7254762; doi:10.1186/s12912-020-00436-8)
Supplement: Supplementary file 4 — Additional file 4. Sources of validity evidence for each measure Identifies supporting data for each source of validity evidence established. [file 12912_2020_436_MOESM4_ESM.docx]

**Additional file 4: Sources of validity evidence for measures (n=35)**

| **LEGEND** | |
| --- | --- |
| **Internal Structure** | |
| **+** | Exploratory Factor Analysis – Principal Components Analysis |
| ***** | Confirmatory Factor Analysis |
| **Relationships to Other Variables** | |
| **+** | Correlation |
| **#** | T-Test/Mann-Whitney U test/Wilcoxon Sign test |
| **♠** | Chi-square |
| **♦** | ANOVA/MANOVA/ANCOVA/MANCOVA/Kruskal-Wallis |
| ♣ | Linear mixed model |
| ***** | Regression |

| **Measure** | **Study** | **Country, Licensure Group** | **Setting** | | | | | | **Source of Validity Evidence** | | | |
| --- | --- | --- | --- | --- | --- | --- | --- | --- | --- | --- | --- | --- |
|  |  |  | **Acute** | **Primary** | **Public**  **Health** | **Home**  **Health** | **Long-term** | **Other/NA** |  |  |  |  |
|  |  |  |  |  |  |  |  |  | **Content** | **Response Process** | **Internal Structure** | **Relationships to Other Variables** |
| **Group 1: Four sources of validity evidence (n=2 measures)** | | | | | | | | | | | | |
| **School nursing evidence-based practice questionnaire**  **(1 study)** | Susan Lynn Adams  (2007) [67] | United States  RNs |  |  |  |  |  |  | Content validation assessed by three experts in EBP, translation research and/or school nursing. | Pilot tested with five school nurses. Feedback used to modify, increase clarity and readability, and add or eliminate questions. | +  Principal components factor analysis:   - Section 1 (Evidence-Based Practice)   Four factors explained 57% of the variance.   - Section 2 (Current practice)   Five factors accounting for 60.9 % of the variance.   - Section 3 (Computer access & skill):   Five factors 57% of the variance.   - Section 4 (information sources):   Three factors explaining 60% of the variance.   - Section 5 (barriers to implementation):   Five factors accounting for 60% of the variance. | +*  Correlation  Current EBP practice with:  Significant at p<.05   - Years RN (.131)   Significant at p<.01   - Diploma/Associate degree (-.263) - Advanced degree (.155) - Professional membership (.256) - Use of web-based resources (.178)   Regression analyses  Dependent variable: current use of EBP  Independent variables: nurse and school characteristics   - 22% variance explained by all variables entered into regression analyses   Significant coefficients:   - Professional membership (β=.114, p=.029) - Use of traditional sources (conferences, other school nurses for practice information) (β=-.102, p=.049) |
| **Evidence-Based Nursing Attitude Questionnaire (EBNAQ)**  **(2 studies)** | M. Ruzafa-Martinez, L. Lopez-Iborra, M. Madrigal-Torres (2011) [135] | Spain  RNs |  |  |  |  |  |  | Six experts evaluated the level of relevance of each item for its corresponding dimension of attitude. The items were classified according to whether the categories representing each dimension were relevant, quite relevant or irrelevant. | Ten nurses were interviewed using this final version. After modifying the items according to the nurses’ suggestions in the instrument pretesting, the quantitative pilot stage was carried out in two rounds. | +  Principal component factor analysis yielded three factors, each with an eigenvalue >1. The total amount of variance explained was 54.70% and each of the items of the subscales loaded onto separate factors.   - Factor 1: ‘Beliefs and expectations towards EBN’ (35.09% variance; eigenvalue 5.26) - Factor 2: ‘Intention of conduct towards EBN’ (11.62% variance; eigenvalue 1.74) - Factor 3: ‘Feelings towards EBN’ (8% variance; eigenvalue 1.20) | +#  Correlation   - Construct validity established: Significant positive correlation between questionnaire scores and an independent measure of attitude towards research. Correlation coefficients were found to be around 0.255 (p < 0.001).   t-test  Significant differences in EBP attitudes based on:   - EBP knowledge (t = 2.261; d.f. 189; p = 0.025)   - Yes (M=59.25; SD=8.94)   - No (M=56.19; SD=7.45) - Experience (t = 2.284; d.f. 188; p= 0.024)   - ≤15 years (M=57.99; SD=7.41)   >15 years (M=54.81; SD=9.73) |
|  | Mohammed Almaskari  (2017) [136] | Oman  RNs |  |  |  |  |  |  | Original findings reported | Not reported | Original findings reported | #♦  t-test  Significant differences in EBP attitudes based on:   - Learned EBP in school t(193)=-2.90; p=0.004   - No (M=3.77; SD=0.37)   - Yes (M=4.11; SD=0.46) - Continuing EBP education t(252)=-2.47, p=0.014   - No (M=3.92; SD=0.43)   - Yes (M=4.06; SD=0.49)   ANOVA   - Education F(2,254)=12.93, p<.001   - Diploma (M=3.88, SD=0.47)   - Specialized diploma (M=4.16; SD=0.44)   - Bachelor’s (M=4.20; 0.41) |
| **Group 2: Three sources of validity evidence (n=5)** | | | | | | | | | | | | |
| **Self-developed measure by Yip et al. (2011)**  **(1 study)** | W. K. Yip, S. Z. Mordiffi, M. S. Majid, E. K. N. Ang (2010) [71] | Singapore  RNs |  |  |  |  |  |  | An expert panel of six nurse leaders and academics reviewed the contents of the survey questionnaire with minor modifications made to the questionnaire. | Not reported | +  Factor analysis with Varimax rotation showed that there were two major factors for all the domains except for the domain of beliefs and attitude, which had only one major factor (factor loadings 0.71-0.73). | *  Two independent variables that predicted positive attitude towards EBP.   - Nurse managers, senior nurse managers, senior nurse educators, senior nurse clinicians (OR=2.67, 95% CI: 1.50-4.76, p = 0.001) more likely to display positive attitude towards EBP than staff nurses - Participants who had attended EBP training course (OR=1.45, 95% CI: 1.01-2.08, p=0.045) were more likely to display positive attitude towards EBP, than staff nurses those who did not attend EBP training. |
| **Quick EBP VIK (Values, Implementation, Knowledge) Survey**  **(2 studies)** | L. Connor, F. Paul, M. McCabe, S. Ziniel  (2017) [75] | United States  APNs, “nurses in any role” |  |  |  |  |  |  | An expert panel of six found 24 of the 26 initial items to be clear and relevant with an Item-Level Content Validity Index (I-CVI) of >0.80 | Not reported | +  Exploratory factor analysis identified three theoretical measurement dimensions (factor loadings):   - Value (.28-.84) - Knowledge (.76-.90) - Implementation (.50-.78) | #  Statistically significant differences between the groups who attended an EBP workshop and those who did not. Lower scores for those who did not attend EBP workshop versus those who did attend EBP workshop [M(SE)]:  *Knowledge*   - Steps of EBP 2.54 (0.05) versus 3.31 (0.07) p<0.001 - How to form PICO question 1.92 (0.06) versus 3.20 (0.09) p<0.001 - Ranking system for hierarchy of evidence 2.00 (0.06) versus 3.02 (0.08) p<0.001 - Performing literature search 2.70 (0.07) versus 3.39 (0.09) p<0.001 - Critically appraising systematic review 1.91 (0.06) versus 2.90 (0.10) p<0.001 - Critically appraising qualitative research study 2.10 (0.06) versus 2.86 (0.09) p<0.001 - Critically appraising quantitative research study 2.05 (0.06) versus 2.90 (0.09) p<0.001 - Frequency of literature searches 1.98 (0.07) versus 2.89 (0.13) p<0.001 - Frequency of critical appraisals of literature search evidence 1.59 (0.06) versus 2.42 (0.11) p<0.001   *Implementation*   - Frequency of performing EBP steps 1.62 (0.06) versus 2.12 (0.08) p<0.001 - Frequency of developing PICO question 1.20 (0.03) versus 1.76 (0.06) p<0.001 - Frequency of having shared EBP process knowledge 1.29 (0.05) versus 1.67 (0.03) p<0.001 - Frequency of having used EBP results to propose change 1.28 (0.03) versus 1.49 (0.06) p<0.002 - Frequency of EBP results having resulted in a change 1.29 (0.04) versus 1.49 (0.06) p<0.005 |
|  | Linda Connor  (2017) [76] | United States  RNs |  |  |  |  |  |  | Original findings reported. | Not reported | +  Factor analysis conducted indicating three factors with eigenvalues exceeding 1 (variance explained):   - Value (45.59%) - Knowledge (11.52%) - Implementation (9.29%) | +♦  Correlation  *Magnet hospitals:*   - positive correlation between level of education and knowledge domain, r (128) = .566, p < .001 - positive correlation between level of education and the implementation domain, r (128) = .518, p < .001. - positive correlation between years of nursing experience and value, r (128) = .214, p = .014 - positive correlation between value and knowledge r (128) = .278, p = .001 - positive correlation between value and implementation r (128) = .284, p = .001 - positive correlation between knowledge and implementation r (128) = .725, p = .001.   *Non-magnet hospitals*   - positive correlation between highest nursing degree and knowledge r (58) = .397, p = .002 - positive correlation between highest nursing degree and implementation r (58) = .353, p = .006 - positive correlation between the knowledge and implementation domains r (58) = .697, p < .001.   ANOVA   - Statistically significant differences between the Magnet® and non-Magnet® participants only for the value domain, F (1, 188) = 6.48, p = .012, partial eta square = .033 |
| **EBP measure developed by Majid et al. (2011; 2 studies)** | J. Farokhzadian, R. Khajouei, L. Ahmadian (2015) [79] | Iran  Licensure group not specified |  |  |  |  |  |  | Three medical informatics specialists and eight faculty members of nursing confirmed content validity. | Not reported | Not reported | +#♦  Correlation   - Significant association between EBP skills and nurses attitudes (r = 0.20, P < 0.01) - Nurses who had positive attitude towards EBP and felt more competent to implement EBP perceived more supporting factors for implementing EBP. Significant association between subscale of supporting factors for EBP (p<0.05) and:   - attitude (r=0.18)   - skills (r=0.20)   t-test   - Significant differences in EBP skills based on attending EBP training (t = 3.87, P < 0.001)   ANOVA  Attitudes towards EBP   - Significant differences in EBP attitudes based on age groups (F = 2.80, P < 0.05) - Significant differences in EBP attitudes based on years of nursing experience (F = 4.24, P < 0.001)   EBP skills   - Significant differences in EBP skills based on years of nursing experience (F = 4.95, P < 0.01) |
|  | Aliyu Adamu, Joanne Rachel Naidoo  (2015) [35] | Nigeria  RNs |  |  |  |  |  |  | Nursing expert evaluated content of questionnaire | Piloted with 20 hospital registered nurses to ensure understandability and comprehensibility. | Not reported | +   - Significant positive association between:   - EBP attitude and age (r=0.137; p=.05)   - EBP knowledge and EBP attitude (r=0.137; p=.01) |
| **Knowledge and Skills in Evidence-Based Nursing (KS-EBN; 1 study)** | M. O. Gu, Y. Ha, J. Kim  (2015) [82] | South Korea  Licensure group not specified |  |  |  |  |  |  | Content validity of assessed by five experts: three nursing scholars and two nurse managers with EBP expertise. Content validity underwent two rounds of expert review and revision. Content validity index (CVI) of each item was calculated, and a CVI of more than  0.8 was interpreted as indicating validity. Final 10 items and scoring rubric had CVI levels of more than .80. | Pilot survey conducted with five nurses (three nurses with experience in EBP projects and two without such experience). They were asked to correct difficult-to-understand or ambiguous questions. | Not reported | #  Construct validity determined using known-groups method   - Significant differences in EBP knowledge and skills scores between EBP and non-EBP group for individual items:   Step #1 item:   - - Asking a clinical question (PICO) – EBP group (M=1.58; SD=0.49) versus non-EBP group (M=0.48; SD=0.69); t=8.63; p<0.001   Step #2 items:   - - Source for the evidence: databases - EBP group (M=0.75; SD=0.25) versus non-EBP group (M=0.23; SD=0.25); t=9.55; p<0.001   - Search terms (keywords) - EBP group (M=0.82; SD=0.36) versus non-EBP group (M=0.24; SD=0.41); t=7.05; p<0.001   - Additional search strategies (Boolean, limits) - EBP group (M=0.68; SD=0.27) versus non-EBP group (M=0.30; SD=0.25); t=7.01; p<0.001   - Limiting searches (study design) - EBP group (M=0.87; SD=0.33) versus non-EBP group (M=0.41; SD=0.49); t=5.06; p<0.001   Step #3 items:   - - Applicability of study findings -EBP group (M=0.86; SD=0.24) versus non-EBP group (M=0.73; SD=0.33); t=2.12; p=0.036   - Validity of RCT - EBP group (M=1.61; SD=0.36) versus non-EBP group (M=1.05; SD=0.65); t=4.93; p<0.001   - Effect size - EBP group (M=0.39; SD=0.49) versus non-EBP group (M=0.18; SD=0.39); t=2.25; p=0.027   - Levels of evidence - EBP group (M=0.66; SD=0.47) versus non-EBP group (M=0.16; SD=0.37); t=5.64; p<0.001 - Significant difference (t=9.93; p<0.001) in subtotal step #2 EBP knowledge and skills scores between EBP group (M= 3.13; SD=0.81) and non-EBP group (M=1.20; SD=1.03) - Significant difference (t=5.72; p<0.001) in subtotal step #3 EBP knowledge and skills scores between EBP group (M= 4.44; SD=1.16) and non-EBP group (M=2.91; SD=1.37) - Significant difference (t=9.51; p<0.001) in total EBP knowledge and skills total scores between EBP group (M= 9.16; SD=1.95) and non-EBP group (M=4.58; SD=2.56) |
| **Perceived EBP Knowledge Measure**  **(1 study)** | L. Thiel, Y. Ghosh  (2008) [137] | United States  RNs |  |  |  |  |  |  | Expert review by three reviewers | Not reported | +  Factor analysis allowed identification of the three items as a single factor with an eigenvalue of 2.1 | +  Significant positive associations between perceived EBP knowledge scores and:   - Unit culture (rho=0.450, p<0.01) - Organizational culture (rho=0.504, p<0.01) - EBP attitudes (rho=0.379, p<0.01) - Education (rho=0.254, p<0.01) - Years in nursing (rho=0.223, p<0.05) |
| **Group 3: Two sources of validity evidence (n=6 measures)** | | | | | | | | | | | | |
| **Modified Stevens EBP Readiness Inventory (ERI) (Finnish ERI)**  **(1 study)** | H. Saunders, K. R. Stevens, K. Vehvilainen-Julkunen  (2016) [80] | Finland  RNs |  |  |  |  |  |  | Content validity of confirmed by a Finnish nurse expert panel consisting of eight nurse scientists, clinicians, educators and leaders. The Content Validity Index (SCVI) for the self-efficacy section of the scale was assessed favorably by the expert panel at 0.90. | Not reported | Not reported | +♦  Correlation   - Significant association between overall self-efficacy score and number of correct responses on EBP knowledge test (r = 0.221)   ANOVA   - Statistically significant difference (p < 0.05) total self-efficacy (i.e. confidence) in employing EBP F (3, 804) = 169.6, P < 0.001 based on four levels of EBP knowledge (no EBP knowledge, beginner, intermediate, advanced). - Post hoc comparisons:   - RNs rating EBP knowledge at an intermediate level were significantly more confident in employing EBP than either those who indicated having no knowledge of EBP (M_diff_= 39.1, P < 0.001) or those who rated themselves at a beginning level (M_diff_= 20.5, P < 0.001). - RNs rating EBP knowledge at a beginning level, were significantly more confident in employing EBP than those who indicated having no knowledge of EBP (M_diff_= 18.6, P < 0.001). |
| **Johns Hopkins Nursing EBP Assessment Survey**  **(1 study)** | K. M. Bissett, M. Cvach, K. M. White (2016) [69] | United States  RNs |  |  |  |  |  |  | Five nursing experts assessed accuracy of competencies | Five nursing experts assessed competencies for face validity, ease of use | Not reported | No supporting validity evidence |
| **Persian translated EBP measure by Seyyedrasooli et al. (2012)**  **(1 study)** | A. Seyyedrasooli, V. Zamanzadeh, L. Valizadeh, F. Tadaion  (2012) [70] | Iran  RNs |  |  |  |  |  |  | Content validity was assessed by 14 faculty members of the Department of Nursing and Midwifery, Tabriz University of Medical Sciences. Any modifications suggested by the experts were applied accordingly. | Not reported | Not reported | #♦  Knowledge   - Education: Statistical difference between Bachelor’s degree (M=13.9; SD=8.1) and Master’s degree (M=22.6; SD=8.8) t(598)=5.43, p=0.001   Attitude   - Education: Statistical difference between Bachelor’s degree (M=32.9; SD=8.1) and Master’s degree (M=33.5; SD=7.3) t(598)=-2.5, p=0.04   Skills   - Higher skills scores in speciality posts compared to other posts (units) F(6)=2.1, p=0.04 |
| **Self-developed EBP measure by Melnyk et al. (2004)**  **(1 study)** | B. M. Melnyk, E. Fineout-Overholt, N. Fischbeck Feinstein, H. Li, L. Small, L. Wilcox, R. Kraus  (2004) [73] | United States  Licensure group not specified |  |  |  |  |  |  | Reviewed for content validity by an interdisciplinary group of EBP experts and with 10 practising nurses. | Not reported | Not reported | +   - Nurses’ beliefs about the importance of EBP in improving patient outcomes and extent to which practices are evidence-based positively correlated (r=.32, p<.001) - Nurses’ beliefs about how much EBP improves clinical care and the extent to which practices are evidence-based positively correlated (r=.40, p<.001) - Nurses with greater EBP knowledge reported greater extent of EBP care (r=.42; p<.0001) - Having greater EBP knowledge positively correlated with EBP initiative involvement (r=.34, p<.001) - Length of time practicing as advanced practice nurse positively correlated with EBP knowledge (r=.37, p<.001) - Higher use of Cochrane Database of Systematic Reviews (r=.43, p<.003) and [www.guideline.gov](http://www.guideline.gov) website (r=.41, p<.001) positively correlated with greater EBP practices - Having mentor positively correlated with high levels of EBP knowledge (r=.28, p<.003) - Having mentor who could role model EBP (r=.21, p<.05) positively correlated with more extensive EBP practices |
| **Self-developed measure by Bostrom et al. (2013)**  **(1 study)** | A. M. Bostrom, A. Rudman, A. Ehrenberg, J. P. Gustavsson, L. Wallin  (2013) [120] | Sweden  RNs |  |  |  |  |  |  | Content validity assessed by group of RNs with expertise in EBP. Content Validity Indices ranged between 0.8 and 1.0. Professional instrument developers from the technical and language laboratory at Statistics Sweden also reviewe items. | Not reported | Not reported | *  Logistic regression conducted for each of the six EBP items/activities as dependent variable with individual and organizational factors as independent variables. Overall models were statistically significant.   - Formulate questions: x^2^=124.7, p<0.001 - Search databases: x^2^=63.9, p<0.001 - Search other sources: x^2^=103.1, p<0.001 - Compile information: x^2^=81.3, p<0.001 - Implement evidence: x^2^=145.6, p<0.001 - Evaluate practice: x^2^=148.9, p<0.001   Significant factors:   - EBP capability beliefs was the only significant factor for all six activities (OR ranged from 2.6-7.3). That is, high capability beliefs associated with high extent of EBP activities. - Supportive leadership and high collective efficacy were associated with a high extent of three EBP activities: searching other knowledge sources (OR = 1.5 and OR = 1.4), implementing evidence (OR = 2.0 and OR = 1.7), and evaluating practice (OR = 1.6 and OR = 1.7). |
| **EBP Competency Tool (Melnyk et al., 2018)** *provided from expert consultation after online search conducted  **(1 study)** | Bernadette Mazurek Melnyk,  Lynn Gallagher-Ford, Cindy Zellefrow,  Sharon Tucker,  Loraine T. Sinnott, Alai Tan (2018) [10] | United States  RNs  APNs |  |  |  |  |  |  | Seven national EBP leaders developed an initial set of competencies for practicing registered nurses and APNs through a consensus building process. Delphi survey was conducted with 80 EBP mentors to determine consensus and clarity around the competencies. (Melnyk et al., 2014) | Not reported | Not reported | +♦*  Correlation  Significant correlations (p<.001) between EBP competency and:   - EBP beliefs (r=.66) - Culture (r=.29) - EBP Knowledge (r=.43) - Mentoring (r=.69)   ANOVA  Total EBP competency scores associated with:   - Age (p<.001)   - <25 years (M=55.6; SD=12.4)   - 25-34 (M=54.8; SD=14.2)   - 35-44 (M=54.0; SD=15.4)   - 44-54 (M=53.3; SD=16.3)   - 55+ (M=51.7; SD=18.0) - Education (p<.001)   - Diploma/associates (M=48.6; SD=15.4)   - Bachelors (M=51.8; SD=14.8)   - Masters or higher (M=63.3; SD=15.5)   Regression  Significant predictors of EBP competency (p<.001):   - Master’s or doctoral degree - Higher EBP knowledge - Higher EBP beliefs - Higher EBP mentoring |
| **Group 4: One source of validity evidence** **(n=19 measures)** | | | | | | | | | | | | |
| **EBP-Implementation Scale (EBPI;**  **(35 studies)** | N. A. Estrada  (2007) [93]  N. Estrada  (2009) [92] | United States  RNs |  |  |  |  |  |  | Not reported | Not reported | +   - Principal component analysis resulted in loading on one factor, eigenvalue =9.16, explaining 51% variance. | *  Regression analyses  Dependent variable: EBP implementation  Independent variable: EBP beliefs subscales  R^2^=0.23, p≤.05  Significant standardized coefficients (p≤.05):  Knowledge beliefs (0.32)  Resource beliefs (0.13)  Difficulty/resources beliefs (0.10) |
|  | B. M. Melnyk, E. Fineout-Overholt, M. Z. Mays  (2008) [84] | United States  Licensure group not specified |  |  |  |  |  |  | Items developed from a review of literature on essential components and steps of EBP. Face and content validity of early drafts assessed in convenience samples of practicing staff nurses (N = 15) and EBP subject-matter experts (N = 8) who reviewed the two questionnaires for content and clarity. | Not reported | +  Principal components analysis indicated single factor:   - Factor #1: Eigenvalue=10.53; 59% variance - Factor #2: Eigenvalue=1.5; 8% variance - Factor loadings of each item in single factor >0.60 | +#♦  Correlations   - EBP implementation and EBP beliefs   - Significantly higher (p=.05) for those who had prior EBP exposure (r=0.51) than those with no EBP exposure (r=0.35)   t-test   - Prior exposure to EBP (p<.001) *test statistic not reported   - No EBP exposure (M=8.60; SD=10.74)   - Prior EBP exposure (M=18.27; SD=16.60)   ANOVA F(4, 331) = 7.46, p < .001   - Education   - Lowest scores associate degree (M=8.37; SD=12.96)   - Highest scores doctoral degree (M=25.50; SD=21.08) - Nursing role F(3, 226) = 6.97, p < .001   - Lowest scores staff nurses (M=10.36; SD=13.54)   - Highest scores Educator/faculty (M = 20.85, SD = 18.71) |
|  | G. Varnell, B. Haas, G. Duke, K. Hudson  (2008) [115] | United States  Licensure group not specified |  |  |  |  |  |  | Not reported | Not reported | Original findings reported | +#  Correlation  EBP-I pre-test scores:   - Education (rho=0.36; p<.05) - Advanced role (rho=0.48; p<.01)   EBP-I post-test scores:  Preceptor role (rho=0.29; o<.05)  t-test  EBP educational intervention (p<.01)   - EBP-Implementation score pre-test (M=15.29; SD=13.65) - EBP-Implementation score post-test (M=22.86; SD=11.35)   EBP-I pre-test scores (p<.01):   - Unfamiliar with EBP (M=10.08; SD=9.25) - Previous exposure to EBP (M=20.95; SD=15.69) |
|  | K. G. Mariano, L. M. Caley, L. Eschberger, A. Woloszyn, P. Volker, M. S. Leonard, Y. Tung  (2009) [104] | United States  RNs |  |  |  |  |  |  | Not reported | Not reported | Not reported | +  Correlation:   - EBP beliefs (r=0.39; p=0.092) |
|  | B. M. Melnyk, T. Bullock, J. McGrath, D. Jacobson, S. Kelly, L. Baba  (2010) [105] | United States  RNs |  |  |  |  |  |  | Original findings reported | Not reported | Not reported | No supporting validity evidence |
|  | R. F. Levin, E. Fineout-Overholt, B. M. Melnyk, M. Barnes, M. J. Vetter  (2011) [101] | United States  RNs |  |  |  |  |  |  | Not reported | Not reported | Original findings reported | ♦  ANOVA with repeated measures  ARCC model program intervention   - main effect for experimental group (M=29.52) compared to control group (M=10.44) on EBP implementation (F1,15 = 10.39, p = .006) at times 3 and 4 - significant quadratic effect of time (F1,15 = 12.40, p = .003) such that there was a significant increase in EBP implementation scores from time 1 (M = 12.89) to time 3 (M = 28.14) in the experimental ARCC group. |
|  | Steve Mooney  (2012) [86] | United States  RNs |  |  |  |  |  |  | Not reported | Not reported | Not reported | No supporting validity evidence |
|  | Lynn Gallagher-Ford  (2012) [85] | United States  RNs |  |  |  |  |  |  | Original findings reported | Original findings reported | Original findings reported | +#♦*  Correlations   - EBP beliefs   - Staff nurses (r=.42; p=.001)   - Educators (r=.54; p=.001)   - Leaders (r=.49; p<.001) - Organizational readiness   - Staff nurses (r=.42; p=.001)   - Educators (r=.34; p=.03)   - Leaders (r=.21; p=.07)   t-test   - Certification (p=.001)   - Certified (M=29)   - Non-certified (M=17) - Current school enrolment (p=.02)   - Enrolled (M=25.9)   - Not enrolled(M=20)   ANOVA   - Role F(2,266)=60.32; p<.001   Post-hoc analyses:   - Staff nurses and educators (M_diff_=-23.29; p<.001) - Staff nurses and leaders (M_diff_=-21.55; p<.001) - Education (F = 14.79, p < .001)   - Associate degree (M=16.9)   - Diploma (M=21.8)   - Master’s (M=40.3)   - Doctorate (M=49.0)   Regression analyses  Dependent variable: EBP implementation  Independent variables:   - EBP beliefs F(1,179)=43.38, p<.001, R^2^=.19 - EBP beliefs and organizational readiness F(1,178)=10.69, p=.001, R^2^=.24 |
|  | Susan Hall Lynch  (2012) [102] | United States  RNs |  |  |  |  |  |  | Original findings reported | Not reported | Original findings reported | +♦  Correlation   - EBP beliefs (r=.334; p=.0001)   ANOVA  Higher EBP implementation scores associated with:   - Certification (F=13.265, p=000) - Higher education (F=15.100, p=.000) - Higher level on clinical ladder (F=5.529, p=.000) |
|  | Yvette M. Pryse  (2012) [107] | United States  RNs |  |  |  |  |  |  | Original findings reported | Not reported | Not reported | *  Regression analyses  Dependent variable: EBP implementation  Independent variables: education, tenure, Magnet status, beliefs, work environment, leadership  Overall multivariate model: (X^2^ = 841.021, df = 8, p < .000)  Multivariate analysis   - EBP beliefs only significant variable (X^2^ = 45.261, df = 1, p < .000)   Univariate analysis (significant variables)   - Leadership (X^2^ = 336.839, df = 1, β = .045, p < .000) - Work environment (X ^2^ = 382.991, df = 1, β = .074, p < .000) - Beliefs (X^2^ = 712.881, df = 1, β = .067, p < .000) |
|  | B. M. Melnyk, E. Fineout-Overholt, L. Gallagher-Ford, L. Kaplan  (2012) [106] | United States  Licensure group not specified |  |  |  |  |  |  | Content validity of the survey was supported by 3 EBP experts | Not reported | Not reported | No supporting validity evidence. |
|  | S. C. Wang, L. L. Lee, W. H. Wang, H. C. Sung, H. K. Chang, M. Y. Hsu, S. C. Chang, C. H. Tai  (2012) [117] | Taiwan  RNs |  |  |  |  |  |  | Back translation to ensure conceptual equivalence of each item. If unsure about content, physician was consulted and discussions took place until consensus reached. | Piloted with an EBP medical centre group (n=9), nursing (n=12) and social work students (n=5) to ensure linguistic appropriateness and utility. | *  The NFI (Normed Fit Index) was above 0.70, and the RMSEA (Root Mean Square Error of Approximation) was less than 0.15 | +#*  ANOVA:   - Education (F=5.87; p=.003)   - Associate (M=0.48)   - Bachelor (M=0.63)   - Master or higher (M=1.12)   Mann-Whitney U-test   - Role   - Clinical nurses (M=0.56)   - Nurse researchers (M=1.97)   Regression analyses   - EBP beliefs, years of conducting research, barriers to research utilization, and ability in literature search predicted EBP implementation (F = 6.84, p = 0.01), accounted for 31.1% of the variance in EBP implementation. |
|  | M. J. Dropkin  (2013) [90] | United States  Licensure group not specified |  |  |  |  |  |  | Not reported | Not reported | Not reported | +  Correlation   - Education (r=.13, p<.01) |
|  | S. Hauck, R. P. Winsett, J. Kuric  (2013) [96] | United States  RNs |  |  |  |  |  |  | Not reported | Not reported | Not reported | ♦   - Role: F (2, 882) = 42.5. p < 0.001) with medium effect size (partial eta squared=.088). - Post hoc comparison using Tukey HSD test:   - Direct Care RNs score (M=0.59; SD=0.61) was significantly different from Indirect Care RNs (M=1.1; SD=0.89) and Director/Leaders (M=1.07; SD=0.77) |
|  | L. Kaplan, E. Zeller, D. Damitio, S. Culbert, K. B. Bayley  (2014) [98] | United States  RNs  LPNs/RPNs  APNs |  |  |  |  |  |  | Not reported | Not reported | Not reported | ♦+  ANOVA   - Education: (F = 8.02 , p < .001)   Correlation   - EBP beliefs: (r = .316, p=.001) - Culture and readiness (r = .198, p = .016) |
|  | K. Stokke, N. R. Olsen, B. Espehaug, M. W. Nortvedt  (2014) [112] | Norway  RNs |  |  |  |  |  |  | Not reported | Not reported | Not reported | +  Correlation   - EBP beliefs (r = 0.59, p = 0.001) - EBP beliefs subscales:   - Attitudes related to knowledge r=0.38 (p < 0.001)   - Attitudes related to resources r=0.29 (p < 0.001)   - Attitudes related to the value of EBP r=0.29 (p < 0.001)   - Attitudes related to difficulty and time r=0.25 (p = 0.001) |
|  | L. H. Eaton, A. R. Meins, P. H. Mitchell, J. Voss, A. Z. Doorenbos  (2015) [91] | United States  RNs |  |  |  |  |  |  | Not reported | Not reported | Not reported | +   - EBP beliefs were associated with nurses’ perceived level of EBP implementation (r = 0.36, p = 0.02) |
|  | M. Underhill, K. Roper, M. L. Siefert, J. Boucher, D. Berry  (2015) [87] | United States  RNs  APNs |  |  |  |  |  |  | Not reported | Not reported | Not reported | +#  Correlation   - Education (r = .32; p = .01)   Mann-Whitney U Test   - Those with formal education have higher EBP implementation scores U=399.0, p=.03 - Those with nurse leader roles have higher EBP implementation scores U=304.5, p=.01 |
|  | Carolyn Sweetapple  (2015) [113] | United States  RNs |  |  |  |  |  |  | Original findings reported | Not reported | Original findings reported | +♦  Correlation   - EBP beliefs (r=.391, p<.001)   ANOVA   - Education: F(3,375)=4.565, p<.01   - Associates degrees/diploma: M=28.64   - Bachelor’s: M=30.95   - Master’s: M=34.86   - Doctorate: M=42.45   *Bonferroni adjustment   - - Doctorate>associates degree/diploma nurses on EPB implementation scores (p<.05)   - Doctorate>bachelor’s on EPB implementation scores (p=.055) - Role F(5,345)=6.396, p<.001   - Directors: M=42.63   - Managers: M=36.69   - Clinical specialists: M=34.20   - Educators: M=31.20   - Staff nurses: M=29.52   - Assistant nurse managers: M=28.28   *Bonferroni adjustment   - - Directors>staff nurses on EBP implementation scores (p<.001) - Experience: F(3,385)=12.481, p<.001   - No experience (M=27.69)   - Some continuing education (M=29.93)   - Formal coursework in EBP (M=35.29)   - Teaching EBP (M=54.00)   *Bonferroni adjustment   - - Nurses who teach EBP>nurses with no experience on implementation scores (p<.001)   - Nurses who teach EBP>nurses with some continuing education on implementation scores (p<.001)   - Nurses who teach EBP>nurses with some formal coursework on implementation scores (p=.001)   - Nurses with some formal coursework>nurses with no experience on implementation scores (p<.01)   - Nurses with some formal coursework>nurses with no some continuing education on implementation scores (p<.01) |
|  | J. Y. Sim, K. S. Jang, N. Y. Kim  (2016) [59] | South Korea  RNs |  |  |  |  |  |  | Not reported | Not reported | Not reported | #♦  t-test  EBP educational intervention   - Experimental group participation in EBP education program had better EBP implementation compare with control group   (t = 3.54, p = .001)  ANOVA repeated measures   - Effect of educational program on experimental group for EBP implementation maintained despite slight decrease (F = 4.68, p = .006)   - Experimental group post-test 1 (after 1 week) (M=2.35; SD=0.74)   - Experimental group post-test 2 (after 4 weeks) (M=2.25; SD=0.85)   - Experimental group post-test 3 (after 8 weeks) (M=2.04; SD=0.76) |
|  | S. C., J. F. Stichler, L. Ecoff, C. E. Brown, A. M. Gallo, J. E. Davidson  (2016) [100]  S. C. Kim, L. Ecoff, C. E. Brown, A. M. Gallo, J. F. Stichler, J. E. Davidson  (2017) [99] | United States  RNs, APNs |  |  |  |  |  |  | Not reported | Not reported | Not reported | +#*  Correlation   - EBP beliefs (r=0.47; p<.001) - Job satisfaction (r=0.17; p=.029) - Mentors (r=0.43; p<.001) - Baccalaureate (r=-0.37; p<.001) - Master’s (r=0.38; p<.001) - Years of RN experience (r=.16; p<.05) - Clinical nurse (r=-0.28); p<.001) - CNS/nurse educator/NP (r=0.32; p<.001)   t-test   - Higher scores of EBP implementation for mentors (M=24.2; SD=16.8) versus fellows (M=11.0; SD=10.6) p<.001 - Statistically significant difference between pre-test (M=15.0; SD=12.7) and post-test (M=24.8; SD=13.7) scores p<.001   Regression analyses  Step 1: Independent variables: constant demographic variables  R^2^=0.225, p<.001  Step 2: independent variables: demographics variables, being a mentor (β=0.27, p<.05), and EBP beliefs (β=0.33, p<.001)  R^2^ Δ=0.075, p<.001 |
|  | B. Skela-Savic, K. Pesjak, B. Lobe  (2016) [110]  B. Skela-Savic, S. Hvalic-Touzery, K. Pesjak  (2017) [109] | Slovenia  RNs, Other |  |  |  |  |  |  | Not reported | Not reported | +   - Two factors explained 68.43% of the total variance with KMO = 0.961 and Barlett P < 0.001. - First factor termed ‘Advanced forms of EBP implementation’ (64.20%, α = 0.963) - Second factor termed ‘Initial forms of EBP implementation’ (4.24%, α = 0.92). Factor loadings for factor #1 - 0.016-0.996; factor loadings for #2 (-0.070-0.927) | +*  Correlations  Skela-Savic et al., 2017  *Advanced forms subscale*  Significant at p<.05   - Age in years (r=0.094)   Significant at p<.01   - Knowledge of research (r=0.326) - Knowledge of EBP (r=0.331) - Job satisfaction (r=0.115)   *Initial forms subscale*  Significant at p<.01   - Age in years (r=0.132) - Length of employment (r=0.114) - Knowledge of research (r=0.291) - Knowledge of EBP (r=0.292) - Job satisfaction (r=0.121)   Skela-Savic et al. 2016   - Positive beliefs on EBP (EBP beliefs subscale) correlated positively with EBP implementation (r=0.486; p=0.000) - EBP implementation correlated negatively with aversion to EBP (EBP beliefs subscale) (r=-0.361; p=0.000) - Knowledge of research (r=0.347, p<.01) - Knowledge o EBP (r=0.328, p<.01) - Job satisfaction (r=0.182, p<.01)   Regression analyses  Skela-Savic et al., 2017  Dependent variable: Advanced forms of EBP implementation R^2^=0.233; p<0.05  Independent variables: Demographic and other respondent characteristics  Significant coefficients:   - Perceived knowledge of EBP (β=0.148; p=0.028   Dependent variable: Initial forms of EBP implementation R^2^=0.200; p<0.05  Independent variables: Demographic and other respondent characteristics  Significant coefficients:   - Knowledge of research (β=0.161; p<.001   Skela-Savic et al. 2016  Dependent variable: EBP implementation R^2^=0.205  Independent variables: Demographic and other respondent characteristics  Significant coefficients:  Perceived knowledge of research (β=0.206; p=0.013)  Perceived knowledge of EBP (β=0.166; p=0.039)  Perceived job satisfaction (β=0.154; p=0.008) |
|  | J. I. Warren, M. McLaughlin, J. Bardsley, J. Eich, C. A. Esche, L. Kropkowski, S. Risch  (2016) [118] | United States  RNs |  |  |  |  |  |  | Not reported | Not reported | Not reported | ♦  ANOVA   - Statistically significant difference between Magnet (M=14.88; SD=13.77) and non-Magnet RNs (M=15.36; SD=14.99) F(1, 1,606) = 145.99, p < .001 - Statistically significant different between ages F(3, 756) = 3.88, p = .009 - Post hoc analyses   - 22-29 years old: (M=12.86; SD=11.14) p<.05   - 30-44 years old: (M=16.01; SD=15.21)   - 45-55 years old: (M=15.17; SD=14.82)   - 55 and older(M=15.63; SD=15.61) - Significant difference based on years employed as RNs F(4, 505) = 5.12, p < .001. - Significant difference based on education   - Associated degree: (M=11.93; SD=12.25)   - Diploma: (M=13.66; SD=15.74)   - Baccalaureate: (M=14.66; SD=13.76)   - Master’s: (M=22.18; SD=17.36), p<.001   - Doctorate: (M=22.22; SD=18), p<.001 - Certified nurses’ (M=19.27; SD=16.42; p<.001) EBP implementation were significantly more favourable than nurses with no certification (M=12.78; SD=12.84) - Nursing role F(2, 392) = 29.95, p < .001   - Nurses in leadership roles had more EBP implementation compared to clinical nurses F(2, 392) = 29.95, p < .001   - Clinical RNs (M=13.06; SD=13.16) mean scores were statistically significantly lower when compared to nurse leaders (M=20.26; SD=15.94) and nurses in support roles (M=19.13; SD=16.5) |
|  | J. I. Warren, K. L. Montgomery, E. Friedmann  (2016) [119] | United States  RNs |  |  |  |  |  |  | Not reported | Not reported | Not reported | ♦  ANOVA   - EBP implementation differed significantly according to roles (F[1,590] = 48.711, p < .001), but did not differ significantly (F[1,632] = 1.562, p = .212) between the 2 years. Nurse leader EBP implementation scores (2008: M = 21.35, SEM = 1.72; 2012: M = 19.08; SEM = 1.43) were higher than those of clinical RNs (2008: M = 10.38, SEM = .92; 2012: M = 12.12, SEM = .87). The difference between the roles did not depend on year (F[1,590] = 2.439, p = .119). |
|  | Kang Younhee, Yang In-Suk  (2016) [97] | Korea  Licensure group not specified |  |  |  |  |  |  | Not reported | Not reported | Original findings reported | +#♦*  t-test  Higher EBP implementation scores for those with:   - Research participation (t=2.409, p=.016) - Positive intention about future research participation (t=2.568, p=.011) - Regularly read research articles (t=4.611, p<.001)   ANOVA  Higher EBP implementation scores for those with:   - High degree of understanding evidence-based nursing practice (F=7.736, p=.001)   Correlation   - EBP beliefs and EBP implementation (r=.287, p<.001) - Barriers to research utilization – communication (r=-.100, p=.049)   Regression analyses   - Overall model significantly explained 17.1% of variance in EBNP implementation (F = 5.560, p< .001)   Significant coefficients   - Regularly read research articles (β=.110, p=.033) - Degree of understanding EBP (β=.113, p=.042) - EBP beliefs (β=.159, p=.004) |
|  | Leonie Rose Bovino, Anne Aquila, Richard Feinn  (2016) [89]  L. Rose Bovino, A. M. Aquila, S. Bartos, T. McCurry, C. E. Cunningham, T. Lane, N. Rogucki, J. DosSantos, D. Moody, K. Mealia-Ospina, J. Pust-Marcone, J. Quiles  (2017) [108] | United States  RNs |  |  |  |  |  |  | Not reported | Not reported | Not reported | +#  Correlation   - EBP beliefs (r=.35, p<.001) - Age (r=-.113, p=.045)   t-test   - Nurses with a baccalaureate or postgraduate degree had higher mean implementation scores (38.2 vs. 31.3; p <.001) than nurses with only an associate degree or a diploma - Bedside nurses had lower mean implementation (32.4 vs. 41.3; p< .001) scores than non-bedside nurses - Nurses who had national certifications had significantly higher implementation scores than those who did not (37.2 vs. 33.1; p = .03). |
|  | Michelle Baxley  (2016) [88] | US  RNs, LPNs/RPNs |  |  |  |  |  |  | Not reported | Not reported | Reported findings for original measure. | +♦*  Correlation   - EBP beliefs (r=.577, p=.0005)   ANOVA   - Significant difference between groups for prior EBP training F (1, 57) = 11.18, p=.001)   - Yes (M=11.50, SD=11.21)   - No (M=3.47, SD=3.09) - Significant difference between groups for education level F (1, 57) = 5.08, p=.028)   - LPN, Diploma, & Associate’s Degree Nurses (M=6.24; SD=6.93)   - Bachelor’s & Master’s Degree Nurses (M=11.95; SD=12.60)   Regression analyses  Dependent variable: EPB implementation  Independent variables: EBP beliefs, EBP training, education level   - Significant regression equation, using the three predictors was computed (F (3, 55) = 9.38, p < .0005), with an R^2^ of .339. - Significant coefficient: EBP beliefs (β=.651, p=.0005) |
|  | H. Verloo, M. Desmedt, D. Morin  (2017) [116] | Switzerland  RNs |  |  |  |  |  |  | Not reported | Not reported | Not reported | *  Regression analyses  13% of the variance in the EBP‐I score was explained by the EBP‐B score (R^2^ = 0.130; P < 0.001) |
|  | M. G. Harper, L. Gallagher-Ford, J. I. Warren, M. Troseth, L. T. Sinnott, B. K. Thomas  (2017) [95] | United States  RNs |  |  |  |  |  |  | Original findings reported | Not reported | Not reported | ♦   - Statistically significant difference between organizations with EBP council (M=43.2; SD=16.8) and without EBP (M=38.5; SD=14.6) council status on EBP implementation scores (Cohen’s d=0.3, p=.02) |
|  | M. A. Friesen, J. M. Brady, R. Milligan, P. Christensen  (2017) [94] | United States  RNs |  |  |  |  |  |  | Not reported | Not reported | Not reported | #   - Change in implementation was significant between preintervention (M=32.9; SD=12.5) and postintervention (M=36.9; SD=17.39) following EBP education mentoring program   (t = 1.75, df = 56, p < .05) |
|  | Kim Son Chae, Jaynelle F. Stichler, Laurie Ecoff, Ana-Mari Gallo, Judy E. Davidson  (2017) [111] | United States  RNs |  |  |  |  |  |  | Not reported | Not reported | Not reported | #   - Six months after fellowship program completion, statistically significant improvements in EBP implementation (M_diff_=3.4, p=.013) |
|  | R. Lovelace, M. Noonen, J. F. Bena, A. S. Tang, M. Angie, R. Cwynar, R. Field, J. Rosenberger, D. Ross, D. Walker, N. M. Albert  (2017) [50] | United States  RNs  LPNs/RPNs |  |  |  |  |  |  | Original findings reported | Not reported | Not reported | +♦  Correlation   - Number of educational modules viewed positively associated with higher EBP implementation (rho=0.24, p<.001) - Nurses with less time since receiving their highest college degree positive associated with EBP implementation (rho=0.23, p<.001)   ANOVA (significant at p<.001)  Factors associated with higher EBP implementation:   - Higher education - Certification - More than one certification - Project leader - Past EBP exposure - Registered nurse-led quality project - Registered nurse-led research - Principal investigator |
|  | Lai Ping Atalanta Wan  (2017) [63] | United States  RNs |  |  |  |  |  |  | Not reported | Not reported | Original findings reported | #   - Following EBP education intervention, nurses in experimental group had significantly higher EBP implementation scores (p=.025) |
|  | Irene Macyk  (2017) [103] | United States  RNs |  |  |  |  |  |  | Not reported | Not reported | Not reported | +*#  Correlation   - EBP belief (r=0.487; p<0.01) - Staff nurse engagement (r=0.394; p<0.01) - Actual decisional involvement (r=0.312, p<0.01)   t-test   - Nurses who were members of a shared governance council had higher EBP implementation scores (M=1.07; SD=0.83) than those who were not members (M=0.55; SD=0.66) (t(l27) = 3.96, p<0.001)   Cohen’s d=0.69  Regression analyses  Dependent variables: EBP implementation  Independent variables: EBP beliefs, staff nurse engagement, shared governance council membership   - overall model was statistically significant (F(3,128) = 21.9, p < 0.001) and the R^2^ was 0.345 and adjusted R^2^ was 0.329   Significant coefficients:   - EBP beliefs (β=0.398, p<0.001) - staff nurse engagement (β=0.241, p<0.01) - shared governance council membership (β=0.168, p<0.05) |
|  | Temple et al. (2014) [114] | Canada  Licensure group not specified |  |  |  |  |  |  | Not reported | Not reported | Not reported | Prior experience with research and having taken a short course on EBP were found to have the strongest relationship in use of evidence in practice in the practicing nurses. *no data provided in conference abstract |
| **EBP-Beliefs Scale** **(EBPB)**  (n=42 studies) | N. A. Estrada  (2007) [93]  N. Estrada  (2009) [92] | United States  RNs |  |  |  |  |  |  | Not reported | Not reported | +  Exploratory factor analysis with varimax rotation performed and identified adequate loading for four factors:  (1) knowledge beliefs  (2) value beliefs  (3) resource beliefs  (4) time and difficulty beliefs. | *  Regression analyses  Significant coefficients - independent variables of learning organization:  **Knowledge:** F=4.71, p≤.01, R^2^=0.06   - None   **Values:** F=9.26, p≤.01, R^2^=0.11   - Connect organization to its environment (β=0.22, p≤.01)   **Resources:** F=12.04, p≤.01, R^2^=0.14   - None   **Time/difficulty:** (not significant)   - Connect organization to its environment (β=0.22, p≤.01)   Regression Analyses for different organizational types:  Independent variables: learning organization items  Dependent variables: Subscales of EBP-Beliefs scale   - Magnet designated   - Knowledge beliefs     - R^2^=.17, p=.01   - Values beliefs     - R^2^=.19, p=.01   - Resources beliefs     - R^2^=.12, p=.01   - Difficulty/time beliefs     - R^2^=.12, p=.01 - Non-magnet designated   - Values beliefs     - R^2^=.09, p=.01   - Resources beliefs     - R^2^=.13, p=.01 - VA hospital   - Knowledge beliefs     - R^2^=.16, p=.01   - Values beliefs     - R^2^=.12, p=.01   - Resources beliefs     - R^2^=.29, p=.01 |
|  | B. M. Melnyk, E. Fineout-Overholt, M. Z. Mays (2008) [84] | United States  Licensure group not specified |  |  |  |  |  |  | Assessed in convenience samples of staff nurses (n=15) and EBP experts (n=8). | Not reported | +  Exploratory Principal Components Analysis:  First factor had an eigenvalue of 6.44 and accounted for 40% of the variance in the scale. Three other factors had eigenvalues > 1.0 (1.8, 1.3, and 1.1, respectively). They accounted for 11%, 8%, and 7% of the variance in the scale, respectively. Single-factor solution was most appropriate interpretation. | +  ANOVA:   - Education: Significant increase in EBP beliefs with level of education, F(4, 344) = 7.03, p < .001   - Associate degree lowest score (M=49.70, SD=19.95)   - Doctoral degree highest score (M=64.06, SD=9.14) - Nursing roles: Significant increase in EBP beliefs from staff nurses to educator/faculty F(3, 233) = 9.34, p < .001   - Staff nurse lowest score (M=48.72, SD=21.63)   - Educator/faculty highest score (M=61.50, SD=8.51) - Age: Significant increase in EBP beliefs with age: F(4, 337) = 5.60, p < .001   - 21 to 30 years lowest score (M=48.35, SD=23.87)   - 61 to 70 years highest score (M=59.75, SD=4.74) |
|  | G. Varnell, B. Haas, G. Duke, K. Hudson (2008) [115] | United States  Licensure group not specified |  |  |  |  |  |  | Not reported | Not reported | Not reported | +#  Correlations:   - Awareness of EBP (learned in nursing school, continuing education, don’t know about EBP) associated with higher pre-test scores of EBP beliefs (r_s_=0.32, p<0.05)   Paired t-test:   - After EBP champion intervention significant differences found between EBP belief pre-test (M=57.10, SD=5.73) and post-test (M=63.73, SD=4.5) scores (p<0.01) |
|  | K. G. Mariano, L. M. Caley, L. Eschberger, A. Woloszyn, P. Volker, M. S. Leonard, Y. Tung [104] | United States  RNs |  |  |  |  |  |  | Not reported | Not reported | Not reported | *  Regression analyses:  Significant coefficients for independent variables of demographics   - Age (β=-0.28, p=0.028) - Years of experience (β=0.37, p=0.006) |
|  | B. M. Melnyk, T. Bullock, J. McGrath, D. Jacobson, S. Kelly, L. Baba  (2010) [105] | United States  RNs |  |  |  |  |  |  | Original findings reported | Not reported | Original findings reported | No supporting validity evidence |
|  | R. F. Levin, E. Fineout-Overholt, B. M. Melnyk, M. Barnes, M. J. Vetter  (2011) [101] | United States  RNs |  |  |  |  |  |  | Not reported | Not reported | Original findings reported | ♦  Intervention: Advancing Research and Clinical practice (ARCC) model intervention   - Significant improvement in ARCC nurses’ EBP beliefs at times 3 and 4, compared with control group. Main effect for group (F1,15 = 33.105, p < .001), a quadratic main effect of time (F1,15 = 7.335, p = .016), and a significant interaction between group and time (F3,45 = 16.342, p = .001). |
|  | D. Hagler, M. Z. Mays, S. B. Stillwell, B. Kastenbaum, R. Brooks, E. Fineout-Overholt, K. M. Williamson, J. Jirsak [128] | United States  Licensure group not specified |  |  |  |  |  |  | Not reported | Not reported | Not reported | #  Intervention EBP workshop   - Following intervention, significant improvement from pre-test (M=59.0, SD=8.4) and post-test (M=66.4, SD=6.8) EBP belief scores, t(146)=12.61, p<0.001 |
|  | B. M. Melnyk, E. Fineout-Overholt, L. Gallagher-Ford, L. Kaplan  (2012) [106] | United States  Licensure group not specified |  |  |  |  |  |  | Content validity supported by 3 EBP experts. | Not reported | Not reported | +  EBP beliefs item: I am clear about the steps in EBP:   - Significantly more Master’s degree nurses than non-Master’s degree nurses indicated they were clear about the steps in EBP (p<0.001) - Levels of education were positively correlated with being clear about the steps in EBP (r = 0.26; p<0.01) |
|  | H. S. Thorsteinsson  (2012) [130]  H. S. Thorsteinsson  (2013) [131] | Iceland  RNs |  |  |  |  |  |  | Translated and reviewed by certified translator and bilingual healthcare professional and Master’s prepared nurses familiar with EBP. Minor modification on wording and order. | Pilot tested with 12 nurses from university and community hospitals for clarity, acceptability, relevance. | +  Principle component analysis  Four factors identified:   - Factor #1: Eigenvalue 5.33 (33.3% variance) - Factor #2 (Eigenvalue 1.8) - Factor#3 (Eigenvalue 1.4) - Factor #4 (Eigenvalue 1.1)   High factor loadings on first factor indicate unidimensionality. | #♦  Strength of EBP beliefs significantly increased:   - With more familiarity of EBP term; F(5, 468) = 44.62, p < 0.001.   - Participants very familiar with EBP term scored highest (M = 64.96, SD = 6.71), whereas those not at all familiar with EBP scored lowest (M = 54.29, SD = 7.22). - With increased participation (frequency) in EBP-related activities   - Identified researchable problems F(3, 459) = 25.48, p≤0.001   - Evaluated research reports F(3, 455) = 42.56, p≤0.001   - Participated in research F(3, 465) = 11.60, p≤0.001   - Used research in practice F(3, 455) = 44.28, p≤0.001 - Based on work setting:   - University hospital nurses scores were higher (M=59.29, SD = 7.29) compared with nurses who worked elsewhere (M=56.85; SD = 6.97; p < 0.001) - Nursing role   - Cinical RNs scores were 57.59 (SD = 6.91) compared with (M=59.51; SD = 7.59) for administrators or educators (p = 0.012) |
|  | S. C. Wang, L. L. Lee, W. H. Wang, H. C. Sung, H. K. Chang, M. Y. Hsu, S. C. Chang, C. H. Tai  (2012) [117] | Taiwan  RNs |  |  |  |  |  |  | Back translation to ensure conceptual equivalence of each item. If unsure about content, physician was consulted and discussions took place until consensus reached. | Piloted with an EBP medical centre group (n=9), nursing (n=12) and social work students (n=5) to ensure linguistic appropriateness and utility. | *  The NFI (Normed Fit Index) was above 0.70, and the RMSEA (Root Mean Square Error of Approximation) was less than 0.15. | ♦#  EBP beliefs scores increased with:   - Education (F=4.58, p<0.011)   - Associate (M=3.19)   - Bachelor (M=3.28)   - Master or higher (M=3.61) - Greater EBP implementation (p<0.001)   - Implement EBP >1 in previous 8 weeks (M=3.33)   - Implement EBP<1 in previous 8 weeks (M=3.06) - Nursing role (p≤0.001)   - Experienced nurse researchers (M=3.92)   - Clinical nurses (M=3.24) |
|  | Steve Mooney  (2012) [86] | United States  RNs |  |  |  |  |  |  | Not reported | Not reported | Not reported | #  Significant difference between pre-test and post-test EBP belief scores after EBP educational intervention (p=0.000)   - Pre-test (M=53.8; SD=5.53) - Post-test (56.4; SD=4.62) |
|  | Lynn Gallagher-Ford  (2012) [85] | United States  RNs |  |  |  |  |  |  | Original findings reported | Original findings reported | Original findings reported | +♦#  ANOVA  Significant EBP belief differences:   - Nurse groups (F = 6.72, p = .001):   - Staff nurses (M=58.3)   - Educators (M=64.4)   - Leaders (M=61.0)   *Post-hoc analysis   - - Significant difference between staff nurses and educators (p=.003) - Education (F = 7.48, p < .001)   - Doctorate degrees (M=68.0)   - Master’s degree (M=66.9)   - Bachelor’s degree (M=59.4)   - Associate degree (M=57.7)   - Diploma degree (M=57.0)   Correlations   - EBP Implementation   - Staff nurses (r=0.42, p=.001)   - Educators (r=0.54, p=.001)   - Leaders (r=0.49, p<.001)   T-test   - Certification (p=.009)   - - Certified nurses (M=62)     - Non-certified (M=58) |
|  | Susan Hall Lynch  (2012) [102] | United States  RNs |  |  |  |  |  |  | Original findings reported | Not reported | Original findings reported | +♦  Correlations   - Age (r=-.120, p=.044) - EBP implementation (r=.334, p=.0001)   ANOVA   - Higher education levels (F=2.829, p=.025) |
|  | Yvette M. Pryse  (2012) [107] | United States  RNs |  |  |  |  |  |  | Original findings reported | Original findings reported | Not reported | Established  +#♦  ANOVA   - Significant difference found among education levels (ASN, BSN, or MSN/NP/DNS/PhD) F(2,418) = 3.042, p = .049   T-test   - Significant difference found among Magnet (M=60.47; SD=7.48) and non-Magnet nurses (M=57.98; SD=8.51); t (419) = 3.183, p =.002   Correlation   - Perceptions of nursing leadership support (r=.430, p<.000) - Perceptions of work environment (r=.486, p<.000) |
|  | M. J. Dropkin  (2013) [90] | United States  Licensure group not specified |  |  |  |  |  |  | Not reported | Not reported | Not reported | No supporting validity evidence |
|  | S. Hauck, R. P. Winsett, J. Kuric  (2013) [96] | United States  RNs |  |  |  |  |  |  | Not reported | Not reported | Not reported | ♦  Intervention: facilitating strategies for evidence-based practice enculturation   - Significant difference between baseline (M=3.67; SD=0.58) and final (M=3.94; SD=0.58) EBP belief scores F(1,899)=52.2, p<0.001 - Significant main effect of time, F (1, 881) = 21.4, p < 0.001 and job role, F (2, 881) = 51.0, p < 0.001 with moderate effect size (partial eta squared = 0.104 on EBP beliefs |
|  | Dawna L. Cato (2013) [127] | United States  RNs |  |  |  |  |  |  | Not reported | Not reported | Original findings reported | No supporting validity evidence |
|  | K. Stokke, N. R. Olsen, B. Espehaug, M. W. Nortvedt (2014) [112] | Norway  RNs |  |  |  |  |  |  | Not reported | Not reported | Not reported | +*  Correlation   - Overall EBP beliefs score and EBP implementation (r = 0.59, p = 0.001) - EBP implementation and EBP beliefs subscales (significant at p≤0.001):   - Knowledge attitudes (r=0.38)   - Resource attitudes (r=0.29)   - Value of EBP (r=0.29)   - Difficulty and time (r=0.25)   Regression analyses  Significant coefficients   - RN Bachelor’s degree (β=-0.31, p=0.04) - Learned about EBP (β=0.15, p=0.04) - Participation in EBP working group (β=0.15, p=0.05) |
|  | L. Kaplan, E. Zeller, D. Damitio, S. Culbert, K. B. Bayley  (2014) [98] | United States  RNs, APNs, LPNs/RPNs |  |  |  |  |  |  | Not reported | Not reported | Original findings reported | +  Correlation   - Organizational culture and readiness (r = .623, p<.001 - EBP implementation (r = .316, p<.001) |
|  | H. S. Thorsteinsson, H. Sveinsdottir  (2014) [132] | Iceland  RNs |  |  |  |  |  |  | Not reported | Not reported | Not reported | *  Regression analyses F (8,283) = 23.919, p < 0.001, R^2^=0.384  Significant coefficients   - EBP skills (β=0.280, p<0.001) - EBP discussions at work (β=0.291, p<0.001) - Familiarity with EBP (β=0.145, p=0.037) |
|  | Natasha Laibhen-Parkes  (2014) [83] | United States  RNs |  |  |  |  |  |  | Not reported | Not reported | Not reported | ♦  Web-based EBP education module   - Significant difference in post-intervention EBP belief scores between control (M=54.6; SD=5.1) and education intervention group (M=60.1; SD=5.1) F=6.44; p=0.005 |
|  | L. H. Eaton, A. R. Meins, P. H. Mitchell, J. Voss, A. Z. Doorenbos [91] | United States  RNs |  |  |  |  |  |  | Not reported | Not reported | Not reported | +  Correlation   - Innovativeness (r=0.48, p=0.002) - EBP implementation (r=0.36, p=0.02) |
|  | M. Underhill, K. Roper, M. L. Siefert, J. Boucher, D. Berry  (2015) [87] | United States  RNs, APNs |  |  |  |  |  |  | Not reported | Not reported | Not reported | +#  Correlation   - Level of nursing education (r = .25; p = .03)   Mann-Whitney U Test   - Formal EBP education – yes/no (U=525.0; p<.01) - Nursing role -direct care/nurse leader (U=554.5, p=.03) |
|  | Debra Hain, Mary Haras  (2015) [133] | United States  RNs, APNs |  |  |  |  |  |  | Not reported | Not reported | Original findings reported | #  EBP educational pre-conference intervention  t-test  Significant difference between pre- and post- EBP belief scores for:   - Combined session #1 and #2: Pre- (M=3.52, SD=0.44) and Post- (M=4.02, SD=0.43); t(51)=8.60, p=0.000 - RN: Pre- (M=3.50, SD=0.45) and Post- (M=3.99, SD=0.42); t(46)=7.92, p=0.000 - APN: Pre- (M=3.71, SD=0.27) and Post- (M=4.29, SD=0.45); t(4)=3.31, p=0.030 - ADN/Diploma: Pre- (M=3.39, SD=0.46) and Post- (M=3.89, SD=0.39); t(12)=7.76, p=0.000 - BSN: Pre- (M=3.48, SD=0.46) and Post- (M=4.02, SD=0.43); t(22)=6.03, p=0.000 - MSN: Pre- (M=3.65, SD=0.33) and Post- (M=4.03, SD=0.42); t(13)=2.69, p=0.018 |
|  | Carolyn Sweetapple (2015) [113] | United States  RNs |  |  |  |  |  |  | Not reported | Not reported | Not reported | +♦  ANOVA   - Significant differences based on education levels F(3, 373)=3.276, p<.05   - Associate degree/diploma: (M=59.2)   - Bachelor’s: (M=61.3)   - Master’s: (M=63.0)   - Doctorate: (M=64.9)   *Bonferroni adjustment: Master’s prepared nurses > beliefs vs. associate degree/diploma nurse (p<.05)   - Significant differences based on EBP experience F(3,382)=11.428, p<.001   - No experience (M=57.4)   - Some continuing education (M=60.8)   - Formal coursework in EBP (M=63.4)   - Teaching EBP (M=69.8)   *Bonferroni adjustment: Significant differences:   - Nurses who teach EBP > beliefs than nurses with no experience (p<.001) and nurses with some continuing education (p<.01) - Nurses with formal coursework > beliefs than those with no experience (p<.001) and those with some continuing education (p<.05)   Correlation   - EBP Implementation (r=.391, p<.001) |
|  | J. Y. Sim, K. S. Jang, N. Y. Kim  (2016) [59] | South Korea  RNs |  |  |  |  |  |  | Not reported | Not reported | Not reported | #  EBP education intervention   - Significant difference in post-test belief scores between experimental (M=3.76; SD=0.41) and control group (M=3.32, SD=0.53), t=3.61, p=.001 |
|  | S. C. Kim, J. F. Stichler, L. Ecoff, C. E. Brown, A. M. Gallo, J. E. Davidson (2016) [100]  S. C. Kim, L. Ecoff, C. E. Brown, A. M. Gallo, J. F. Stichler, J. E. Davidson  (2017) [99] | United States  RNs, APNs |  |  |  |  |  |  | Not reported | Not reported | Not reported | +#  Regional EBP fellowship program intervention  t-tests   - Significant difference between pretest (M=61.7; SD=7.12) and posttest (M=67.3; SD=6.01) scores (p<.001) - Role (p<.001)   - Mentor (M=66.6; SD=6.91)   - Fellows (M=59.3; SD=6.38)   Correlation   - Job satisfaction (r=.27; p=.003) - Mentor role (r=0.48; p<.001) - Diploma/associate (r=-0.19; p<.05) - Baccalaureate (r=-0.43; p<.001) - Master/doctorate (r=0.51; p<.001) - Clinical nurse (r=-0.33; p<.001) - CNS/nurse educator/NP (r=0.34; p<.001) - EBP implementation (r=0.47; p<.001) |
|  | B. Skela-Savic, K. Pesjak, B. Lobe  (2016) [110]  B. Skela-Savic, S. Hvalic-Touzery, K. Pesjak  (2017) [109] | Slovenia  RNs  “Other” |  |  |  |  |  |  | Not reported | Not reported | +  Skela-Savic et al. (2016)  Yielded three factors explaining 57.88% of total variance:   1. Factor #1: Positive beliefs on EBP (44.36%) 2. Factor #2: Aversion to EBP (8.57%) 3. Factor #3 (4.93%)   Skela-Savic et al. (2017)  Yielded three factors explaining 58.29% of total variance:   1. Factor #1: Knowledge and Skills on EBP (45.21%) 2. Factor #2: Effects of EBP on clinical practice (8.67%) 3. Factor #3 (4.41%) | +*  Skela-Savic et al. (2016)  Correlations  Factor #1: Positive beliefs on EBP   - EBP implementation (r=0.486, p=0.000) - Factor #2 Aversion to EBP (r=-0.361, p=0.000) - Length of employment (r=0.120, p=0<.05) - Knowledge of research (r=0.452, p<0.01) - Knowledge of EBP (r=0.448, p<0.01) - Job satisfaction (r=0.314, p<0.01)   Factor #2 Aversion to EBP   - EBP implementation (r=-0.361, p=0.000) - Age (r=--0.176, p<0.01) - Length of employment (r=-0.166, p=0<0.01) - Knowledge of research (r=-0.286, p<0.01) - Knowledge of EBP (r=-0.417, p<0.01) - Job satisfaction (r=-0.234, p<0.01)   Regression analyses  Dependent variable Factor #1: Positive beliefs on EBP, R^2^=0.311  Significant coefficients   - Perceived knowledge of research (β=0.240; p=0.007) - Perceived job satisfaction (β=0.236; p=0.000) - Length of nursing employment (β=0.159; p=0.008)   Dependent variable Factor #2: Aversion to EBP, R^2^=0.222  Significant coefficients   - Perceived knowledge of research (β=-0.333; p=0.000) - Training and education in EBP in nursing (β=0.191; p=0.022)   Skela-Savic et al. (2017)  Correlations  Factor #1 Knowledge and Skills on EBP:  Significant at p<.05   - Educational achievement (r=0.103)   Significant at p<.01   - Age (r=0.124) - Length of employment (r=0.122) - Knowledge of research (r=0.464) - Knowledge of EBP (r=0.477) - Job satisfaction (r=0.256)   Factor #2 Effects of EBP on clinical practice  Significant at p<.01   - Training and education in research (r=0.213) - Training and education in EBP (r=0.291)   Regression analyses  Dependent variable: Factor #2 Effects of EBP on clinical practice; R^2^=0.289, p<.05  *Demographic and respondent characteristics*  Significant coefficients:   - Training and education in EBP (β=0.165, p=.004) |
|  | J. I. Warren, M. McLaughlin, J. Bardsley, J. Eich, C. A. Esche, L. Kropkowski, S. Risch  (2016) [118] | United States  RNs |  |  |  |  |  |  | Not reported | Not reported | Not reported | ♦  ANOVA   - Age F(3, 750) = 4.37, p = .005   - 22-29 years (M=59.22; SD=8.33) p<.05   - 30-44 years (M=58.58; SD=8.55)   - 45-54 years (M=57.18; SD=8.71)   - 55 and older (M=57.28; SD=9.4) - Years employed as RN F(4, 513) = 5.20, p < .001 - Education *no F-statistic provided but states statistically significant difference   - Diploma (M=55.23; SD=9.43)   - Associate degree (M=56.21; SD=8.68)   - Baccalaureate (M=58.02; SD=8.75)   - Master’s (M=61.64; SD=7.56) p<.001   - Doctorate (M=65.89; SD=8.62) p<.001 - Certification F(1, 1137) = 18.78, p < .001   - Certified (M=59.21; SD=8.73) p < .001   - Not certified (M=57.2; SD=8.7) - Nursing role F(2, 446) = 21.42, p < .001   - Leadership (M=60.71; SD=8.71) p < .001   - Support service RN (M=59.53; SD=8.18) p < .001   - Clinical RN (M=57.04; SD=8.8) |
|  | J. I. Warren, K. L. Montgomery, E. Friedmann  (2016) [119] | United States  RNs |  |  |  |  |  |  | Not reported | Not reported | Not reported | ♣  Linear mixed model   - EBP beliefs depended on both year and nurses’ roles (F[1,576] = 4.435, p = .036, interaction effect) - Nurse leader scores were significantly higher than clinical RN scores for both years, but the difference was greater in 2008 (nurse leader: M = 61.15, SEM = 1.23; clinical RN: M = 53.85, SEM = 0.65) than in 2012 (nurse leader: M = 60.60., SEM = 0.96; clinical RN: M = 57.07, SEM = 0.58) |
|  | Kang Younhee, Yang In-Suk  (2016) [97] | Korea  Licensure group not specified |  |  |  |  |  |  | Not reported | Not reported | Not reported | +   - EBP beliefs and EBP implementation were significantly positively correlated (r = .287, p<.001) |
|  | Leonie Rose Bovino, Anne Aquila, Richard Feinn  (2016) [89]  L. Rose Bovino, A. M. Aquila, S. Bartos, T. McCurry, C. E. Cunningham, T. Lane, N. Rogucki, J. DosSantos, D. Moody, K. Mealia-Ospina, J. Pust-Marcone, J. Quiles  (2017) [108] | United States  RNs |  |  |  |  |  |  | Not reported | Not reported | Not reported | +#  t-test   - Direct care nurses had significantly lower beliefs scores than non-direct care nurses (60.1 vs. 65.7; p<.001) - Nurses with a baccalaureate or postgraduate degree had higher beliefs scores than did those with an associate degree or a diploma (63.6 vs. 59.2; p<.001   Correlation   - Beliefs correlated positively with implementation (r = .35, p < .001) |
|  | Michelle Baxley  (2016) [88] | United States  RNs, LPNs/RPNs |  |  |  |  |  |  | Not reported | Not reported | Original findings reported | +  Correlation   - EBP implementation (n = 59, r = .57, p = .0005) |
|  | Sherri L. Smith-Keys  (2016) [129] | United States  RNs |  |  |  |  |  |  | Not reported | Not reported | Not reported | No supporting validity evidence |
|  | R. Lovelace, M. Noonen, J. F. Bena, A. S. Tang, M. Angie, R. Cwynar, R. Field, J. Rosenberger, D. Ross, D. Walker, N. M. Albert  (2017) [50] | United States  RNs, LPNs/RPNs |  |  |  |  |  |  | Original findings reported | Not reported | Not reported | +♦  Correlation   - Number of educational models viewed: (rho=0.12, p<.001) - Nurses with less time since receiving their highest college degree were more likely to have positive beliefs about the value of EBP (rho = 0.20; p < .001)   ANOVA *No F-statistic, means or SD reported  Significant at p<.001   - Higher education - Certification - Project leader - Past exposure EBP - RN nurse-led quality project - Principal investigator - Literature review completed   Significant at p=.042   - Higher clinical ladder level |
|  | H. Verloo, M. Desmedt, D. Morin  (2017) [116] | Switzerland  RNs |  |  |  |  |  |  | Not reported | Not reported | Not reported | *   - 13% of the variance in the EBP‐I score was explained by the EBP‐B score (R^2^ = 0.130; P < 0.001) |
|  | M. G. Harper, L. Gallagher-Ford, J. I. Warren, M. Troseth, L. T. Sinnott, B. K. Thomas  (2017) [95] | United States  RNs |  |  |  |  |  |  | Original findings reported | Not reported | Not reported | +   - Organizations with EBP councils had statistically significant higher levels of EBP beliefs (p = .03) |
|  | M. A. Friesen, J. M. Brady, R. Milligan, P. Christensen  (2017) [94] | United States  RNs |  |  |  |  |  |  | Not reported | Not reported | Not reported | No supporting validity evidence |
|  | Kim Son Chae, Jaynelle F. Stichler, Laurie Ecoff, Ana-Mari Gallo, Judy E. Davidson (2017) [111] | United States  RNs |  |  |  |  |  |  | Not reported | Not reported | Not reported | +#*  t-test  Fellowship program intervention   - Statistically significant improvements in EBP beliefs 6-months post-intervention (mean difference=6.6; p<.001   Correlation   - EBP adoption at 6 months post-intervention   (r = 0.35, p = .001)  Regression   - EBP beliefs emerged as a positive predictor of EBP adoption (OR=1.12; 95% confidence interval, 1.02-1.22; p = .017) |
|  | Lai Ping Atalanta Wan  (2017) [63] | United States  RNs |  |  |  |  |  |  | Not reported | Not reported | Original findings reported | #  EBP educational intervention   - Significant increase between pre-test and post-test scores in experimental group (p=.046) |
|  | Irene Macyk  (2017) [103] | United States  RNs |  |  |  |  |  |  | Not reported | Not reported | Not reported | +*  Correlation   - Actual decisional involvement (r=0.260; p<0.01) - EBP implementation (r=0.487; p<0.01) - Staff nurse engagement (r=0.235; p<0.05)   Regression analyses  Dependent variable: EBP Implementation  Independent variables: EBP beliefs, nurse engagement, shared governance council participation (F(3,128) = 21.9, p < 0.001), R^2^=0.345   - EBP beliefs predicative factor (β=0.398; p<0.001) |
|  | Temple et al. (2014) [114] | Canada  Licensure group not specified |  |  |  |  |  |  | Not reported | Not reported | Not reported | - Prior experience with research and having taken a short course on EBP were found to have the strongest relationship in a more positive belief about use of evidence. *no data provided in conference abstract |
| **Evidence-Based Practice Questionnaire (EBPQ)** | D. Upton, P. Upton (2006) [30] | Wales  Does not specify licensure group |  |  |  |  |  |  | Initial pool of items generated from literature review and discussions with healthcare professionals | Piloted 3 times: initial pool with 33 senior healthcare professionals; after revisions draft piloted in two rounds with initial group of healthcare professionals in addition to a steering group of experts in the field of health and social care policy | Principal component factor analysis yielded three factors, each with an eigenvalue >1 (61.77% explained in total):   1. Practice of EBP (33.08%) 2. Attitude towards EBP (17.07%) 3. Knowledge/skills associated with EBP (11.63%)   Each of the items of the subscales loaded onto separate factors. Items were considered as contributing to a subscale if they had a factor loading of at least 0.4 on that factor. | +#  Correlation coefficients between subscale scores and awareness of EBP ranged from 0.3-0.4 (p<0.001) indicating positive but moderate relationship.  Those with knowledge of local EBP initiative compared to those without knowledge had better attitude (t=2.5; d.f.=332; p<0.01), more frequent EBP practice (t=3.2; d.f.=360; p<0.02) and better EBP knowledge (t=5.2; d.f.=360; p<0.001) |
|  | M. L. Koehn, K. Lehman  (2008) [49] | United States  RNs |  |  |  |  |  |  | Not reported | Not reported | Not reported | ♦  Statistically significant differences found among four educational levels (diploma, ADN, BSN, MSN) on EBP practice, attitude, knowledge/skills subscale scores Wilk’s Λ=0.93, F=3.16 (p=0.001)  Use of EBP:   - Diploma (M=4.98; SD=1.23) - ADN (M=5.01; SD=1.41) - Baccalaureate (M=4.92; SD=1.35) - Master’s (M=5.65; SD=0.76)   Knowledge/Skills of EBP:   - Diploma (M=4.56; SD=0.95) - ADN (M=4.60; SD=1.00) - Baccalaureate (M=4.66; SD=1.01) - Master’s (M=5.15; SD=0.68)   Attitudes towards EBP:   - Diploma (M=5.03; SD=1.06) - ADN (M=4.90; SD=1.12) - Baccalaureate (M=5.34; SD=1.08) - Master’s (M=5.59; SD=0.86)   ANOVA conducted as f/u to MANOVA. ANOVA on attitude scores was statistically significant, F(3, 403)=6.01, p=0.001. Post-hoc analyses determined BSN group had statistically significant higher attitude scores compared to ADN group (p<0.003). |
|  | C. E. Brown, M. A. Wickline, L. Ecoff, D. Glaser  (2009) [41] | United States  Does not specify licensure group |  |  |  |  |  |  | Not reported | Not reported | Not reported | +  Correlation between “Characteristics of the Communication” (i.e., perceive research difficult to find and understand) subscale of BARRIERS scale and “Knowledge/Skill of EBP” subscale (r=-0.216, p<0.05).  Correlation between “Characteristics of the Organization” subscale of BARRIERS scale and “Knowledge/Skills of EBP” subscale (r=-0.179, p=0.004). |
|  | C. E. Brown, L. Ecoff, S. C. Kim, M. A. Wickline, B. Rose, K. Klimpel, D. Glaser  (2010) [40] | United States  RNs |  |  |  |  |  |  | Not reported | Not reported | Not reported | +*  Bivariate correlations with EBPQ subscales:  Significant (p≤0.05)  **Practice of EBP:**   - Nurse manager role (0.13)   **Attitudes towards EBP:**   - CNS Nurse Educator (0.10) - Organization BARRIERS subscale (-0.09) - Innovation BARRIERS subscale (-0.08)   **Knowledge/skills of EBP:**   - years of RN experience (0.08) - Nurse manager (0.13)   Significant (p≤0.01)  **Practice of EBP:**   - Age (0.10) - Years of RN experience (0.10) - Master’s degree (0.11) - Staff nurse (-0.20) - Communication BARRIERS subscale   (-0.13)  **Attitudes towards EBP:**   - Master’s degree (0.11) - Staff nurse (-0.18) - Adopter BARRIERS subscale (-0.12) - Communication BARRIERS subscale   (-0.15)  **Knowledge/skills of EBP:**   - Baccalaureate degree   (-0.15)   - Master’s (0.20) - Doctoral (0.18) - Staff nurse (-0.19) - Adopter BARRIERS subscale (-0.16) - Organization BARRIERS subscale (-0.16) - Communication BARRIERS subscale   (-0.20)  Regression analyses  Significant coefficients  **Practice of EBP**  Regression model; *R^2^*=2.7%, p≤0.001)  *Demographic variables*   - Years of RN experience (β =0.07, p≤0.05) - Staff nurse (β=-0.09; p≤0.01) - Master’s degree   (β =0.07, p≤0.05)  *BARRIERS subscales*   - Adopter (β=-0.10; p≤0.05) - Innovation (β=0.14; p≤0.001) - Communication   (β=-0.16; p≤0.001)  **Attitude towards EBP**  Regression model; *R^2^*=2.4%, p≤0.001)  *Demographic variables*   - Staff nurse (β=-0.09; p≤0.05) - Master’s degree (β =0.08, p≤0.05)   *BARRIERS subscales*   - Adopter (β=-0.12; p≤0.01) - Communication (β=-0.11; p≤0.001)   **Knowledge/Skills associated with EBP**  Regression model; *R^2^*=4.5%, p≤0.001)  *Demographic variables*   - Doctoral degree (β=-0.17; p≤0.001) - Master’s degree (β =0.18, p≤0.001)   *BARRIERS subscales*   - Adopter (β=-0.12; p≤0.01) - Innovation (β=0.11; p≤0.01) - Communication (β=-0.15; p≤0.001)   **Note for BARRIERS subscales:*  **Adopter:** nurses’ values and awareness of research  **Organization:** nurses’ perception about limitations/barriers at work  **Innovation:** nurses’ perception about quality of research  **Communication:** nurses’ perceptions about presentation and accessibility of research |
|  | P. Prior, J. Wilkinson, S. Neville  (2010) [55] | New Zealand  RNs |  |  |  |  |  |  | Not reported | Not reported | Not reported | +  **Practice of EBP:**   - Level of registration preparation (r_s_=0.306; p=0.026) - Frequency of reading journals (r_s_=0.303; p=0.028) - Length of time practicing as a registered nurse   (r_s_=-0.288; p=0.038)   - Length of time practicing in primary health care (r_s_=-0.312; p=0.024)   **EBP Knowledge/Skills:**   - Practice of EBP (r_s_=0.744; p=0.00) - Attitudes towards EBP (r_s_=0.532; p=0.000) - Length of time practicing in primary health care (r_s_=-0.412; p=0.004) - Level of registration preparation (education) (r_s_=0.528; p=0.000) - Tertiary qualification (post-registration) (r_s_=0.351; p=0.016)   **Attitudes Towards EBP:**   - Practice of EBP (r_s_=0.516; p=0.00) |
|  | S. Gonzalez-Torrente, J. Pericas-Beltran, M. Bennasar-Veny, R. Adrover-Barcelo, J. M. Morales-Asencio, J. De Pedro-Gomez  (2012) [44] | Spain  Does not specify licensure group |  |  |  |  |  |  | Not reported | Not reported | Not reported | ♦*  ANOVA; no test statistics reported  **EBPQ Total:** Group differences based on years of professional experience (p=0.018)   - 0 to 2 years (M=127.3) - 2 to 10 years (M=115.6) - 10 to 20 years (M=112.5) - >20 years (M=110.3)   **Knowledge/Skills of EBP:** Group differences based on years of professional experience (p=0.023)   - 0 to 2 years (M=72.3) - 2 to 10 years (M=66.1) - 10 to 20 years (M=63.9) - >20 years (M=62.4)   **Attitudes toward EBP:**  Nurses with management functions > clinical nurses (p=0.008)  Regression analyses  Dependent variable: EBPQ  Independent variables: Nurse work index, gender, professional category (e.g., management-supervision/coordination), years of practice  Significant coefficients:  Years of practice: (B=-0.733; p=0.004) |
|  | M. J. Linton, M. A. Prasun  (2013) [33] | United States  Does not specify licensure group |  |  |  |  |  |  | Not reported | Not reported | Not reported | +  Correlations (p<0.05)  Age and:   - Ability to identify gaps in your professional practice (r=0.285) - Knowledge of how to retrieve evidence (r=0.203) - Ability to critically analyze evidence (r=0.236) - Ability to apply information to individual cases (r=0.307) - Evaluate outcomes of your practice (r=0.179)   Education and:   - Ability to identify gaps in your professional practice (r=0.377) - Knowledge of how to retrieve evidence (r=0.368) - Ability to critically analyze evidence (r=0.379) - Ability to apply information to individual cases (r=0.323) - Evaluate outcomes of your practice (r=0.257) |
|  | White-Williams, P. Patrician, P. Fazeli, M. A. Degges, S. Graham, M. Andison, A. Shedlarski, L. Harris,McCaleb (2013) [64] | United States  RNs |  |  |  |  |  |  | Not reported | Not reported | Not reported | +♦  Correlations:  **EBPQ total:**   - Education (r=0.23; p<0.01) - Certification (r=0.12; p<0.01) - Job title (r=0.18; p<0.01) - Attended any EBP research or PD workshops offered by Centre for Nursing Excellence (r=-0.16; p<0.01) *1=yes, 2=no   **Practice of EBP:**   - Education (r=0.12; p<0.01) - Job title (r=0.17; p<0.01) - Attended any EBP research or PD workshops offered by Centre for Nursing Excellence (r=-0.19; p<0.01) *1=yes, 2=no - EBPQ total (r=0.75; p<0.01)   **Attitudes towards EBP:**   - Education (r=0.26; p<0.01) - Employment status (r=-0.11; p<0.05) - Job title (r=0.18; p<0.01) - Attended EBP and Research Council (r=-0.15; p<0.01) - Attended any EBP research or PD workshops offered by Centre for Nursing Excellence (r=-0.20; p<0.01) - EBPQ total (r=0.61; p<0.01) - EBP Practice (r=0.40; p<0.01)   **Knowledge/Skills towards EBP:**   - Education (r=0.21; p<0.01) - Certification (r=0.12; p<0.01) - Job title (r=0.16; p<0.01) - Attended any EBP research or PD workshops offered by Centre for Nursing Excellence (r=-0.12; p<0.01) - EBPQ total (r=0.95; p<0.01) - Practice of EBP (r=0.55; p<0.01) - Attitudes towards EBP (r=0.44; p<0.01)   MANCOVA: Dependent variables: EBPQ total and three subscales   1. Independent variable: EBP and Research Council attendance  - Significant multivariate main effect Wilk’s λ = 0.982,   F(3, 471) = 2.88, p = .035   - Univariate tests: Attitudes subscale significantly higher for those with EBP attendance (M=5.75) vs. those with no EBP attendance (M=5.23) p<0.05   2. Independent variable: attendance at any EBP education class/workshop   - Significant multivariate main effect Wilk’s λ = 0.965,   F(3, 471) = 5.725, p = 0.001   - Univariate tests showed significant higher scores for those who attended vs. no attendance (p<0.01) for: - EBPQ total: M=4.74 vs. M=4.51 - Practice: M=3.65 vs. M=3.29 - Attitudes: M=5.78 vs. M=5.49 |
|  | B. M. Toole, J. F. Stichler, L. Ecoff, L. Kath  (2013) [62] | United States  RNs |  |  |  |  |  |  | Not reported | Not reported | Not reported | +#  Significant differences between pretest and post-test EBPQ practice scores for:   - Computer-based learning EBP education: (M [pre-test]=3.74; SD=1.41; M [post-test]=4.41; SD=1.13; p=0.002) - In-class EBP education:   (M [pre-test]=3.63; SD=1.36; M [post-test]=4.10; SD=1.24; p=0.006)  Correlations:  **EBPQ practice:**  Significant at p<0.01   - # formal EBP classes/in-service   (r=0.205)   - # of own readings in EBP (r=0.308) - # EBP computer-based education (r=0.220) - # EBP conferences (r=0.273) - EBP knowledge/skills (r=0.613) - EBP attitudes (r=0.281)   Significant at p<0.05   - Age (r=0.108)   **Attitudes toward EBP:**  Significant at p<0.01   - # of own readings in EBP (r=0.213) - EBP knowledge/skills (r=0.410)   **Knowledge/Skills of EBP:**  Significant at p<0.01   - # formal EBP classes/in-service   (r=0.215)   - # of own readings in EBP (r=0.352) - # EBP computer-based education (r=0.237)   Significant at p<0.05   - # EBP conferences (r=0.147) |
|  | Son Chae Kim, Caroline E. Brown, Laurie Ecoff, Judy E. Davidson, Ana-Maria Gallo, Kathy Klimpel, Mary A. Wickline  (2013) [48] | United States  RNs |  |  |  |  |  |  | Not reported | Not reported | Not reported | +#*  Paired t-tests  Following EBP Fellowship intervention:  **Practice of EBP**   - Overall: Significant difference in pre-test (M=4.52) and post-test (M=5.33) scores; t=5.91, p<0.001 - Fellows: Significant difference in pre-test (M=4.14) and post-test (M=5.09) scores; t=4.46, p<0.001 - Mentors: Significant difference in pre-test (M=4.95) and post-test (M=5.60) scores; t=3.95, p<0.001   **Knowledge/skills of EBP**   - Overall: Significant difference in pre-test (M=4.64) and post-test (M=5.24) scores; t=9.06, p<0.001 - Fellows: Significant difference in pre-test (M=4.13) and post-test (M=5.11) scores; t=7.36, p<0.001 - Mentors: Significant difference in pre-test (M=4.82) and post-test (M=5.38) scores; t=5.68, p<0.001   Bivariate correlations at post-test:  **Practice of EBP:**  Significant at p<0.05   - Mentor (r=0.24) - Baccalaureate (r=-0.22) - Staff nurse position (r=-0.28) - CNS/nurse educator/NP (r=0.25) - Communication BARRIERS subscale (r=-0.22)   Significant at p<0.01   - Organization BARRIERS subscale (r=-0.25)   **Attitude towards EBP:**  Significant at p<0.05   - Baccalaureate (r=-0.19) - Staff nurse position (r=-0.21) - CNS/nurse educator/NP (r=0.25) - Innovation BARRIERS subscale (r=-0.19)   Significant at p<0.01   - Organization BARRIERS subscale (r=-0.27)   **Knowledge/skills of EBP:**  Significant at p<0.05   - Mentor (r=0.22) - CNS/nurse educator/NP (r=0.20)   Significant at p<0.01   - Baccalaureate (r=-0.31) - Master’s/doctoral (r=0.32) - Staff nurse (r=-0.26) - Organization BARRIERS subscale (r=-0.26) - Innovation BARRIERS subscale (r=-0.28) - Communication BARRIERS subscale (r=-0.32)   Regression analyses  **Practice of EBP**  *Demographic variables*  Regression model; *R^2^*=9.7%, p<0.05  *Barriers variables*  Regression model; *R^2^*=6.8%, p<0.05   - No predictor variables reached statistical significance   **Attitudes toward EBP**  *Demographic variables*  Regression model; *R^2^*=7.6%, p<0.01  *Barriers variables*  Regression model; *R^2^*=8.9%, p<0.01   - Organization BARRIERS subscale (β =-0.35, p≤0.01)   **Knowledge/Skills of EBP**  *Demographic variables*  Regression model; *R^2^*=10%, p<0.001  *Barriers variables*  Regression model; *R^2^*=13.9%, p<0.001   - Innovation BARRIERS subscale (β =-0.21, p≤0.05) |
|  | Sese-Abad, J. De Pedro-Gomez, M. Bennasar-Veny, P. Sastre, J. C. Fernandez-Dominguez, J. M. Morales-Asencio (2014) [57] | Spain  RNs |  |  |  |  |  |  | Not reported | Not reported | Confirmatory Factor Analysis:  Inadequate fit for 24-item model vs. 19-item model in four subsamples  Goodness of fit indexes:  **Subsample 1** (staff in large hospitals n=415):  *24-item model*  x^2^=1720.32, df= 249, p < .0001  x^2^/df=6.91 RMSEA=.12 90% CI=[.11, .12]  p (RMSEA) > .05 <.0001 SRMR=.08  *19-item model*  x^2^=301.31, df= 149, p < .0001  x^2^/df=2.02 RMSEA=.05 90% CI=[.05, .06]  p (RMSEA) > .05 =.16 SRMR=.04  *Model comparison*  x^2^=1419.01, df=100, p<.0001  **Subsample 2** (staff in medium-sized hospitals n=611):  *24-item model*  x^2^=2566.73, df= 249, p < .0001  x^2^/df=10.31 RMSEA=.12 90% CI=[.12, .13]  p (RMSEA) > .05 <.0001 SRMR=.08  *19-item model*  x^2^=366.98, df= 149, p < .0001  x^2^/df=2.46 RMSEA=.05 90% CI=[.05, .06]  p (RMSEA) > .05 =.19 SRMR=.04  *Model comparison*  x^2^=2199.75, df=100, p<.0001  **Subsample 3:** (staff in small hospital centres n=270)  *24-item model*  x^2^=1367.95, df= 249, p < .0001  x^2^/df=5.49 RMSEA=.13 90% CI=[.12, .14]  p (RMSEA) > .05 <.0001 SRMR=.09  *19-item model*  x^2^=263.96, df= 149, p < .0001  x^2^/df=1.77 RMSEA=.06 90% CI=[.05, .07]  p (RMSEA) > .05 =.06 SRMR=.05  *Model comparison*  x^2^=1103.99, df=100, p<.0001  **Subsample 4:** (primary care n=377)  *24-item model*  x^2^=1818.74, df= 249, p < .0001  x^2^/df=7.30 RMSEA=.13 90% CI=[.12, .14]  p (RMSEA) > .05 <.0001 SRMR=.09  *19-item model*  x^2^=329.27, df= 149, p < .0001  x^2^/df=2.21 RMSEA=.06 90% CI=[.05, .07]  p (RMSEA) > .05 =.01 SRMR=.05  *Model comparison*  x^2^=1489.47, df=100, p<.0001 | +  Correlations:  **EBP Practice:**   - Attitudes toward EBP (r=0.57; p<0.0001) - Knowledge/skills EBP (r=0.62, p<0.0001)   **Attitudes Toward EBP:**   - Knowledge (r=0.36, p<0.0001) |
|  | M. A. Perez-Campos, I. Sanchez-Garcia, P. L. Pancorbo-Hidalgo (2014) [53] | Spain, Latin America  RNs |  |  |  |  |  |  | Not reported | Not reported | Not reported | +♦  Correlations  EBPQ total:   - Academic level (rho=0.303, p≤0.01) - Professional category (rho=0.221, p≤0.01) - More favourable evaluation of environment (rho=0.147, p≤0.05)   ANOVA  **EBP Practice:**   - Professional category: F=5.22, p=0.002   - Registered Nurse (M=4.59; SD=1.53)   - Clinical nurse specialist (M=5.35; SD=1.26)   - Ward manager (M=5.04; SD=1.55)   - Direction (M=4.69; SD=1.79) - Academic level: F=11.06, p<0.001   - Diploma (M=4.49; SD=1.48)   - Bachelor degree (M=5.26; SD=1.45)   - Master degree/PhD (M=5.27; SD=1.39)   **EBP Attitudes:**   - Practice environment: F=5.99, p=0.003   - Unfavourable (M=5.08; SD=1.26)   - Mixed (M=5.58; SD=1.04)   - Favourable (M=5.53; SD=1.09) - Academic level: F=7.66, p=0.001   - Diploma (M=5.51; SD=1.05)   - Bachelor degree (M=5.73; SD=1.16)   - Master degree/PhD (M=5.56; SD=1.15)   **EBP Knowledge:**   - Professional category: F=8.41, p<0.001   - Registered Nurse (M=4.56; SD=1.15)   - Clinical nurse specialist (M=5.26; SD=1.14)   - Ward manager (M=5.04; SD=1.11)   - Direction (M=5.20; SD=1.18) - Practice environment: F=4.89, p=0.008   - Unfavourable (M=4.63; SD=1.24)   - Mixed (M=4.97; SD=1.03)   - Favourable (M=5.12; SD=1.12) - Academic level: F=16.20, p<0.001   - Diploma (M=4.54; SD=1.04)   - Bachelor degree (M=5.23; SD=1.14)   - Master degree/PhD (M=5.25; SD=1.11)   **EBPQ total**   - Professional category: F=5.46, p=0.001   - Registered Nurse (M=4.81; SD=1.09)   - Clinical nurse specialist (M=5.39; SD=1.00)   - Ward manager (M=5.17; SD=1.20)   - Direction (M=4.96; SD=1.36) - Academic level: F=15.46, p<0.001   - Diploma (M=4.72; SD=0.98)   - Bachelor degree (M=5.41; SD=1.11)   - Master degree/PhD (M=5.36; SD=1.06) |
|  | E. Shafiei, A. Baratimarnani, S. Goharinezhad, R. Kalhor, M. Azmal (2014) [58] | Iran  Does not specify licensure groups |  |  |  |  |  |  | Content validity was established using a peer review method | Not reported | Not reported | +  Correlations:  **EBP Practice:**   - Attitude toward EBP (r=0.222; p=0.004) - Knowledge/skills of EBP (r=0.734; p<0.001)   **Attitude toward EBP:**  Knowledge/skills of EBP (r=0.443; p<0.001) |
|  | A. Ammouri, A. A. Raddaha, P. Dsouza, R. Geethakrishnan, J. A. Noronha, A. A. Obeidat, L. Shakman (2014) [39] | Oman  RNs |  |  |  |  |  |  | Not reported | Not reported | Not reported | *+  Correlations:  **EBP Practice**   - Years of experience (r=0.160, p<0.001)   **Attitudes toward EBP**   - Years of experience (r=0.202, p<0.001) - Years of last academic qualification (r=-0.091, p<0.05)   **Knowledge/skills of EBP**   - Academic qualification (r=0.076, p<0.05) - Years of last academic qualification (r=-0.101, p<0.05)   Regression analyses:  Significant coefficients:  **EBP Practice:** F (4, 409) = 3.79, p<0.01, R^2^=0.036   - Years of experience (β =0.170, p<0.01) - Barriers to finding/reviewing research (β=−0.242; p <0.001)   **Attitudes toward EBP:** F (4, 409) = 3.45, p<0.01, R^2^=0.042   - Years of experience (β =0.197, p<0.001) - Barriers to finding/reviewing research (β=−0.280; p <0.001)   **Knowledge/skills of EBP** F (4, 409) = 2.82, p<0.05, R^2^=0.017   - Years of last academic qualification (β =-0.109, p<0.05) - Barriers to finding/reviewing research (β=−0.306; p <0.001)   *years of last academic qualification (nurses needed to report if last qualification was after 2005) |
|  | Y. J. Son, Y. Song, S. Y. Park, J. I. Kim  (2014) [60] | Korea  RNs |  |  |  |  |  |  | Not reported | Piloted with the nurses to ensure that its cultural and linguistic adaptations were appropriate. No reported problems in understanding the scale, nor was cultural rewording necessary | +*  Exploratory factor analysis  EFA yielded three factors (64.4% variance explained in total):   1. Practice of EBP (7.597%) 2. Attitude towards EBP (6.277%) 3. Knowledge/skills associated with EBP (50.495%)   *one item moved from attitude to practice  Confirmatory factor analysis  Model 1 (original factor structure): x^2^=5.79 (p < 0.001), SRMR=0.08, NFI=0.81, CFI=0.84  Model 2 (revised factor structure): x^2^=5.59 (p < −0.001), SRMR=0.06, NFI=0.85, CFI=0.85  Model 2 identified better fit. | +  Correlations (p<0.001)  Critical thinking disposition:   - EBP Knowledge/skills (r=0.398) - EBP Practice (r=0.318) - EBP Attitude (r=0.212) |
|  | J. B. Carlone, O. Igbirieh  (2014) [42] | Qatar  RNs |  |  |  |  |  |  | Not reported | Not reported | Not reported | +#♦  Spearman’s rank correlations (p<0.001)  **EBP Practice**   - Attitude (r_s_=0.321) - Knowledge/skills (r_s_=0.471) - Level of education (identified as significant but no data provided)   **EBP Attitudes**   - Knowledge/skills (r_s_=0.299) - Age (identified as significant but no data provided)   Kruskal-Wallis H Test   - Significant difference between total score and clinical speciality (H= 26.588, p=0.014) - Significant difference between EBP Practice score and clinical speciality (H= 30.214, p=0.004)   Mann-Whitney U Test   - Urology (M=161) differed significantly in its total score when compared to the rest of the sample (M=124) (U=41.5, p=0.041)   Urology (M=42, U=24, p<0.001) and Emergency/Trauma (M=32.5) U=629, p=0.041 differed significantly when compared to rest of sample |
|  | Jed Duff, Margaret Butler, Menna Davies, Robyn Williams, Jannelle Carlile  (2014) [43] | Australia  RNs |  |  |  |  |  |  | Reported findings for original measure. | Not reported | Not reported | No support validity evidence |
|  | Susanne Tacaraya Fehr  (2014) [34] | United States  RNs |  |  |  |  |  |  | Not reported | Researcher conducted paper-pencil pilot with sample of 5 medical-surgical nurses. | Not reported | +♦*  Correlations:  **EBPQ total score:**   - Nursing informatics competency (r=0.548, p<0.01) - Self-efficacy (r=0.248, p<0.01)   ANOVA:  **EBPQ total score:**   - Nursing degree (F=3.10, p=0.17)   - Diploma (M= 4.16; SD=.827)   - Associates (M=5.13; SD=.829)   - Bachelors, traditional (M=5.06; SD=.867)   - Bachelors, 2^nd^ degree (M=5.27; SD=.822)   - MSN (M=5.55; SD=.666)   Regression analyses:  Significant coefficients  **EBPQ total score:** F=7.34, p<0.001, R^2^=0.43   - Nursing informatics competency (B =0.63, p=0.000) - Self-efficacy (B =0.22, p=0.006) - Age (under 35 years vs >50 years)   (B =0.56, p=0.002) |
|  | H. T. Xie, Z. Y. Zhou, C. Q. Xu, S. Ong, A. Govindasamy  (2015) [66] | Singapore  Does not specify licensure group |  |  |  |  |  |  | Not reported | Not reported | Not reported | No supporting validity evidence |
|  | Nicole Allen, Barbara G. Lubejko, Julie Thompson, Barbara S. Turner  (2015) [38] | United States  Does not specify licensure group |  |  |  |  |  | (does not specify ‘other’) | Not reported | Not reported | Not reported | No supporting validity evidence |
|  | Aliyu Adamu, Joanne Rachel Naidoo  (2015) [35] | Nigeria  RNs |  |  |  |  |  |  | An expert in nursing research, to evaluate the content of the questionnaire. | Not reported | Not reported | +#  Correlations:  **EBP Attitude:**   - Age (r_s_=0.137, p<0.05) - Knowledge (r_s_=0.137, p<0.01)   Independent t-test  Significant difference in knowledge between junior and senior nurses; t(14)=2.526, p<0.05 (no mean scores provided) |
|  | R. P. Pereira, A. C. Guerra, M. J. Cardoso, A. T. dos Santos, C. de Figueiredo Mdo, A. C. Carneiro  (2015) [52] | Portugal  Does not specify licensure group |  |  |  |  |  |  | Not reported | Not reported | *  Revised 20 item structure underwent confirmatory factor analysis:  New model was tested and goodness of fit was obtained: χ2 (167) = 520.009;  p = 0.0001; χ2df = 3.114; CFI = 0.908;  GFI = 0.865; PCFI = 0.798; PGFI = 0.678; RMSEA = 0.077 (CI 90%=0.07-0.08). | No supporting validity evidence |
|  | J. I. Hwang, H. A. Park  (2015) [47] | Korea  Does not specify licensure group |  |  |  |  |  |  | Not reported | Not reported | Indicates that a principal components factor analysis using the varimax rotation method was performed to test the validity of the EBPQ. However, no findings regarding this are presented. | +*  **EBPQ total scores:**  Significant differences between groups related to:   - Age (F=7.92; p<0.001)   - 20-30 (M=4.0; SD=0.7)   - 31-40 (M=4.2; SD=0.8)   - 41-60 (M=4.4; SD=0.9) - Education (F=13.84; p<0.001)   - 3-year diploma (M=3.9; SD=0.7)   - 4-year baccalaureate (M=4.1; SD=0.7)   - Master or higher (M=4.5; SD=0.9) - Years in Nursing (F=4.84; p=0.003)   - <3 (M=3.9; SD=0.7)   - 3-<5 (M=4.0; SD=0.7)   - 5-<10 (M=4.1; SD=0.7)   - 10 or more (M=4.3; SD=0.9) - Job position (t=4.16; p<0.001)   - Manager (M=4.5; SD=0.9)   - Staff (M=4.1; SD=0.7)   Regression analyses:   - Higher age and education significantly associated with EBPQ total scores (F=10.98; p<0.001)   - Age (β =0.01, p=0.029)   - Master degree or higher (β =0.43, p<0.001)   Correlations:  Identifies positive moderate correlation between EBPQ and quality improvement scores, and between individual EBPQ subscales and total score, however does not identify which are statistically significant. |
|  | C. Phillips  (2015) [54] | United States  Does not specify licensure group |  |  |  |  |  |  | Not reported | Not reported | Not reported | +  Correlations:  **Practice of EBP**   - Attitudes (r=0.813, p=0.000) - Knowledge (r=0.844, p=0.000) - Total EBPQ (r=0.936, p=0.000)   **Attitudes towards EBP**   - Knowledge (r=0.848, p=0.000) - Total EBPQ (r=0.907, p=0.000)   **Knowledge of EBP**  Total EBPQ (r=0.973, p=0.000) |
|  | A. J. Ramos-Morcillo, S. Fernandez-Salazar, M. Ruzafa-Martinez, R. Del-Pino-Casado  (2015) [56] | Spain  RNs |  |  |  |  |  |  | Not reported | Not reported | +  Exploratory factor analysis with principal axis factors and Varimax rotation showed the previous three dimensions, which accounted for 72.5% of the variance. | ♦  Between-subjects effects (EBP intervention)  EBP Knowledge and skills F(1)=6.6, p=0.01 |
|  | K. M. Williamson, M. Almaskari, Z. Lester, D. Maguire  (2015) [65] | United States  RNs |  |  |  |  |  |  | Not reported | Not reported | Not reported | #  Mann-Whitney U test  Significant differences between staff nurses and management for:   - EBP knowledge/skills (U = 1153, p = .001) - EBP attitude (U = 1072, p = .000)   EBP practice (U = 1123, p = .001) |
|  | J. R. Duffy, S. Culp, C. Yarberry, L. Stroupe, K. Sand-Jecklin, A. Sparks Coburn  (2015) [31]  J. R. Duffy, S. Culp, K. Sand-Jecklin, L. Stroupe, N. Lucke-Wold (2016) [32] | United States  RNs |  |  |  |  |  |  | Not reported | Not reported | Not reported | +#  Correlations:  EBP attitudes with individual EBP Practice items of:   - Participating in implementing research knowledge in practice (r_s_=0.299, p=0.011)   EBP confidence with individual EBP Practice items of:   - Formulate questions (r_s_=0.424, p<0.001) - Seek out relevant knowledge using databases (r_s_=0.544, p<0.001) - Seek out relevant knowledge using other information sources (r_s_=0.558, p<0.001) - Critically appraise and compile best knowledge (r_s_=0.538, p<0.001) - Participate in implementing research knowledge (r_s_=0.265, p=0.022)   EBP knowledge with individual EBP Practice items of:   - Formulate questions (r_s_=0.460, p<0.001) - Seek out relevant knowledge using databases (r_s_=0.298, p=0.011) - Seek out relevant knowledge using other information sources (r_s_=0.314, p=0.007) - Critically appraise and compile best knowledge (r_s_=0.346, p=0.003) |
|  | Donna Agnew  (2016) [37] | United States  Does not specify licensure group |  |  |  |  |  |  | Not reported | Not reported | Not reported | #♦  **EBPQ total score**   - Certified nurses had higher scores (t=1.996, p=0.047) - Managers/directors and advanced practice nurses had higher scores (F=8.905, p<0.001) than staff nurses   **EBP Knowledge/Skills**   - Managers/directors and advanced practice nurses had higher scores (F=9.6, p<0.001) than staff nurses   **EBP Attitudes**   - Managers/directors had higher scores (F=4.498, p=0.012) compared to staff nurses   **EBP Practice**  Managers/directors had higher scores (F=6.567, p=0.002) compared to staff nurses |
|  | J. Y. Sim, K. S. Jang, N. Y. Kim  (2016) [59] | South Korea  RNs |  |  |  |  |  |  | Not reported | Not reported | Not reported | #  T-tests for post-test scores  Following EBP educational intervention:   - **Knowledge/skill of EBP:** Significant difference between experimental (M=5.04) and control (M=4.31) groups, t=3.08, p=0.003 - **Attitude toward EBP:** Significant difference between experimental (M=5.47) and control (M=4.55) groups, t=4.10, p<0.001 |
|  | R. Lovelace, M. Noonen, J. F. Bena, A. S. Tang, M. Angie, R. Cwynar, R. Field, J. Rosenberger, D. Ross, D. Walker, N. M. Albert  (2017) [50] | United States  RNs  LPNs/RPNs |  |  |  |  |  |  | Reported findings for original measure. | Not reported | Reported findings for original measure. | +♦  **EBP attitude**   - Number of EBP educational modules viewed positively associated with higher EBP attitudes (*rho*=0.082; p=0.009) - Less time since receiving their highest college degree have higher EBP attitudes (*rho*=0.17; p<0.001)   Factors associated with higher EBP attitudes *only p-values reported for following associations:  Significant at p<0.001   - Higher education - Certification (yes) - More than one certification - Project leader (yes) - Past exposure to EBP - Registered nurse-led quality project (yes) - Principal investigator (yes) - Literature review completed ≤6 months (yes)   Significant at p=0.039  Work full-time (vs. part-time) |
|  | A. Hagedorn Wonder, A. M. McNelis, D. Spurlock, P. M. Ironside, S. Lancaster, C. R. Davis, M. Gainey, N. Verwers  (2017) [45] | United States  RNs |  |  |  |  |  |  | Not reported | Not reported | Not reported | +  Correlations:  **EBP Practice**   - EBP attitudes (r=0.350, p<0.01) - EBP knowledge/skills (r=0.595, p<0.01) - Age (r=0.202, p<0.05) - Years of RN experience (r=0.168, p<0.05) - “I am sure I can deliver evidence-based care” (r=0.294, p<0.01)   **EBP Attitudes**   - EBP knowledge/skills (r=0.398, p<0.01) - “I am sure I can deliver evidence-based care” (r=0.228, p<0.01)   **EBP Knowledge/Skills**   - “I am sure I can deliver evidence-based care” (r=0.413, p<0.01) |
|  | D. C. Stavor, J. Zedreck-Gonzalez, R. L. Hoffmann  (2017) [61] | United States  RNs |  |  |  |  |  |  | Not reported | Not reported | Not reported | No supporting validity evidence |
|  | M. O. A. Hasheesh, M. E. AbuRuz  (2017) [46] | Saudi Arabia  RNs |  |  |  |  |  |  | Not reported | Not reported | Not reported | +*  Correlations:  **Practice of EBP:**   - Age (r=-0.18, p<0.01) - Qualification (r=0.23, p<0.01) - Years of experience (r=-0.12, p<0.05) - EBP training (r=0.11, p<0.05) - Research involvement (r=0.20, p<0.01)   **EBP Attitudes:**   - Qualification (r=0.2, p<0.01)   **EBP Knowledge/skills:**   - Qualification (r=0.12, p<0.05)   Regression analyses:  Significant coefficients identified for demographic variables *p-values not stated  **Practice of EBP** R^2^ = 0.12; F(6,296) = 7.07, P<0.001   - Education (β =0.25)   **EBP Attitudes** R^2^ = 0.049; F(6,296) = 3.53, P<0.05   - Education (β =0.20)   **EBP Knowledge/Skills** R^2^ = 0.038; F(6,296) = 2.69, P<0.05   - Education (β =0.16) |
|  | Lora Moore  (2017) [51] | United States  RNs |  |  |  |  |  |  | Not reported | Not reported | Reported findings for original measure. | No supporting validity evidence |
|  | Mohannad Eid AbuRuz, Haneen Abu Hayeah, Ghadeer Al-Dweik, Hekmat Yousef Al-Akash  (2017) [36] | Jordan  RNs |  |  |  |  |  |  | Not reported | Not reported | Not reported | +*  Correlations:  **EBP Practice:**   - Educational level (0.066, p<0.1)   **EBP Attitudes:**   - Educational level (0.101, p<0.05) - Participation in research (0.142, p<0.01) - Data base access (0.146, p<0.01)   **EBP Knowledge/skills:**   - Educational level (0.116, p<0.01) - Participation in research (0.201, p<0.01) - Data base access (0.235, p<0.01)   Regression analyses:  Significant coefficients with demographic variables  **EBP Practice** R^2^=0.061; F(4,495)=9.11, p<0.001   - Participation in research (β =-0.11, p<0.05) - Data base access (β =-0.171, p<0.001)   **EBP Attitudes** R^2^=0.054; F(4,495)=7.12, p<0.001   - Educational level (β =0.093, p<0.05) - Data base access (β =0.121, p<0.01)   **EBP Knowledge/skills** R^2^=0.074; F(4,495)=10.89, p<0.001   - Educational level (β =0.1, p<0.05) - Participation in research (β =0.1, p<0.05) - Data base access (β =0.206, p<0.001) |
|  | Lai Ping Atalanta Wan  (2017) [63] | United States  RNs |  |  |  |  |  |  | Not reported | Not reported | Not reported | #  Wilcoxon signed-rank text  **EBP Knowledge/Skills**  Statistically significant difference between pre-test (M=9.39) and post-test (M=10.83) scores after EBP educational intervention (p=0.34) in experimental group |
| **Developing Evidence-based practice questionnaire (Gerrish et al., 2007; 6 studies)** | K. Gerrish, P. Ashworth, A. Lacey, J. Bailey, J. Cooke, S. Kendall, E. McNeilly (2007) [122] | England  *Does not specify licensure group |  |  |  |  |  |  | Content validity of the questionnaire was considered by a panel of four experts in community health nursing and minor modifications made to the questionnaire | Not reported | +  Exploratory factor analysis identified ten factors (variance %):   - Factor 1. Skill in finding, reviewing and using different sources of evidence (10.622%) - Factor 2. Barriers to, or facilitators of, personal efficacy in the context of the organization, including team culture and personal authority (10.622%) - Factor 3. Published information as a source of knowledge used in practice. (10.622%) - Factor 4. Focal concern or interest in the effective use of research. (10.622%) - Factor 5. The availability of formal information (research and organizational information), and disposable time to implement the recommendations. (10.622%) - Factor 6. Knowledge gleaned from training, conferences, and local and national reports and audits. (10.622%) - Factor 7. Personal experience. (10.622%) - Factor 8. Informal information gleaned in the course of daily work, including interprofessional conversations. (10.622%) - Factor 9. The facilitating or hindering effect of colleagues in changing practice. (10.622%)   Factor 10. Client /patient contact and the nurse’s personal knowledge and experience. (10.622%) | +   - Significant positive associations between skills in finding and reviewing evidence and facilitation and support in changing practice (r=0.197; p<0.01) |
|  | K. Gerrish, P. Ashworth, A. Lacey, J. Bailey  (2008) [123] | England  RNs |  |  |  |  |  |  | Not reported | Not reported | Original findings reported | No supporting validity evidence |
|  | J. Mills, J. Field, R. Cant  (2009) [124] | Australia  RNs, LPNs/RPNs |  |  |  |  |  |  | Not reported | Not reported | Not reported | +   - Significant association between younger age of nurses and perceptions of skills in use of the library to locate information (r=−.140; p<.01) and skills in using the Internet (r=−.243; p<.01) - Significant association (p<.01) between level of nursing qualification and:   - Using internet to search for information (r=.209)   - Using library to locate information (r=.215)   - Using organizational information to change practice (r=.177)   - Reviewing organizational information (r=.203)   - Finding organizational information (r=.180)   - Using research evidence to change practice (r=.143)   - Finding research evidence (r=.201)   Reviewing research evidence (r=.242) |
|  | Kate Gerrish, Jo Cooke (2013) [8] | England  *Does not specify licensure group |  |  |  |  |  |  | Not reported | Not reported | Not reported | No supporting validity evidence |
|  | L. M. Baird, T. Miller (2015) [125] | Canada  RNs, APNs |  |  |  |  |  |  | Not reported | Not reported | Not reported | ♦  Significant differences:   - Education and skills: F(2 ,60)=7.349, p=0.001 (eta squared 0.20)   - Diploma (M=2.3; SD=0.48)   - Bachelor (M=2.0; SD=0.56)   - Master (M=1.2; SD=0.38)   Post-hoc analyses: Significant difference between those with highest education (Master’s) and other two groups. |
|  | J. I. Shin, E. Lee  (2017) [126] | South Korea  RNs |  |  |  |  |  |  | Not reported | Not reported | Not reported | +♦*  Correlation  Significant associations (p<.05) between skills in finding and reviewing evidence and:   - Barriers to finding and reviewing evidence   (r=-.46)   - Barriers to changing practice (r=-.23) - Facilitators and support to changing practice (r=.27)   ANOVA   - Nurses with higher social capital (social dimensions of the nursing environment) scores showed higher scores (F= 7.513, p = .001)   Regression analyses   - Social capital explained 11% of the variance in Skills Appraisal in Finding and Reviewing Evidence (F = 2.37, p < .001) |
| **Modified Evidence-Based Nursing**  **Education Questionnaire (EBEQ)**  **(1 study)** | S. Hellier, T. Cline  (2016) [74] | United States  APNs |  |  |  |  |  |  | Not reported | Not reported | Not reported | +♦#  Correlation  Age with:   - Overall EBEQ scores (r=-.113, p=.029) - Knowledge of EBP (r=-.168, p<.001) - Changing practice based on evidence (r=-.112, p=.017)   Number of years practicing as NP:   - negatively correlated with knowledge of EBP scores (r = −.195, p < .001)   ANOVA  Education with:   - Overall EBEQ score F=7.369, p=.000 - Knowledge score F=5.510, p=.000 - Finding evidence score F=5.960, p=.000 - Changing practice based on evidence score F=3.338, p=.010 - Judging evidence score F=12.979, p=.000   Post-hoc tests:   - DNPs scored significantly higher than Master’s prepared NPs, on:   - overall EBEQ score (mean difference=9.28, p<.001)   - finding evidence factor (mean difference=1.96, p=.002)   - judging evidence factor (mean difference=3.50, p<.001) - DNPs scored significantly higher than bachelor degree holders on knowledge of EBP (mean difference = 6.69, p = .004)   t-test   - Participants who are employed at Magnet-designated facilities reported marginally higher scores (M = 21.42, SD = 2.99) than did non-Magnet participants (M = 20.27, SD = 3.89); t(431)=2.61; p=.01 |
| **Self-developed measure by Barako et al. (2012; 1 study)** | Talaso D. Barako, Margaret Chege, Sabina Wakasiaka, Lilian Omondi  (2012) [77] | Kenya  Licensure group not specified |  |  |  |  |  |  | Not reported | Not reported | Not reported | #*  t-test   - Nurses who fully practised EBP had more positive attitude (M= 4.41) compared to those who do not fully practice EBP (M= 3.76), t =3.34, p =0.001.   Regression analyses  Dependent variable: EBP application  Independent variables: demographic characteristics  Significant factors associated with EBP application:   - Nursing education (p=0.029)   - Certificate (OR=1.00)   - Diploma (OR=0.31)   - Bachelor’s (OR=6.00) - Ability to critically review EBP literature (p=0.021)   - Confident (OR=1.00)   - Fairly confident (OR=0.75)   - Poorly confident (OR=0.06)   Attitudes towards EBP (OR=0.49; p=0.007): Nurses with negative EBP attitudes less likely to practice EBP |
| **Self-developed measure by Gerrish et al. (2011)**  **(1 study)** | K. Gerrish, L. Guillaume, M. Kirshbaum, A. McDonnell, A. Tod, M. Nolan  (2011) [81] | England  APNs |  |  |  |  |  |  | Not reported | Not reported | Not reported | ♠  Chi-squared tests indicated statistically significant differences (P < 0.001) between nurses with Master’s qualifications and above and those with a bachelor degree or below. APNs with Master’s qualifications were more likely to view themselves as competent/expert in all the identified skills associated with evidence-based practice:   - Obtaining sources of evidence via WWW x^2^= 29.817 - Undertaking literature searches x^2^= 40.425 - Evaluating research reports x^2^= 38.266 - Adapting national guidelines for local implementation x^2^= 28.673 - Setting evidence-based standards x^2^= 30.977 - Undertaking clinical audit x^2^= 24.488 - Undertaking benchmarking x^2^= 39.615 - Identifying need for change based on evidence x^2^= 28.254 - Implementing changes in practice x^2^= 30.285 - Evaluating effects of changes made in practice x^2^= 21.489 - Undertaking research x^2^= 38.881 |
| **Adapted Fresno Test**  **(1 study)** | Natasha Laibhen-Parkes  (2014) [83] | United States  RNs |  |  |  |  |  |  | Not reported | Not reported | Not reported | #  t-test   - Intervention group (EBP education) had statistically higher test scores (M=94.1; SD=32.4) compared to control group (M=80.3; SD=24.3); t=1.31, p=0.20 - Hedge’s g=0.47 |
| **Self-developed measure by Kim et al. (2013)**  **(1 study)** | Son Chae Kim, Caroline E. Brown, Laurie Ecoff, Judy E. Davidson, Ana-Maria Gallo, Kathy Klimpel, Mary A. Wickline  (2013) [48] | United States  RNs |  |  |  |  |  |  | Not reported | Not reported | Not reported | #  Significant differences (p<.001) between pre- and post-test scores of perceived abilities to implement EBP steps following EBP fellowship intervention:   - Formulate a key clinical question: M_diff_=0.86; t=10.04 - Search databases: M_diff_=0.61; t=7.54 - Find best clinical evidence: M_diff_=0.82; t=9.53 - Understand research articles: M_diff_=0.61; t=7.61 - Appraise articles critically: M_diff_=0.73; t=8.78 - Synthesize research articles: M_diff_=0.73; t=9.07 - Apply evidence to patient care: M_diff_=0.73; t=8.22 |
| **Evidence-Based Practice Confidence Scale (EPIC)**  **(1 study)** | J. R. Duffy, S. Culp, C. Yarberry, L. Stroupe, K. Sand-Jecklin, A. Sparks Coburn  (2015) [31]  J. R. Duffy, S. Culp, K. Sand-Jecklin, L. Stroupe, N. Lucke-Wold  (2016) [32] | United States  RNs |  |  |  |  |  |  | Not reported | Not reported | Not reported | +#♦  Correlation  EBP confidence and:   - EBP use (r = 0.538, p<.001) - Formulate questions to search for research- based knowledge (ρ=0.424; p<.001) - Seek out relevant knowledge using databases (ρ=0.544, p<.001) - Seek out relevant knowledge using other information sources (ρ=0.558, p<.001) - Critically appraise and compile best knowledge (ρ=0.538, <.001) - Participate in implementing research knowledge in practice (ρ=0.265, p=.022)   t-test   - Statistically significant difference in EBP confidence between those who had professional certification (M=64.2%; SD=14.7%) and those who did not (M=55.2%; SD=18.8), p= .025)   Kruskal-Wall’s  Statistically significant difference in EBP confidence by education level (p=.013):   - Diploma/associate degree M=49.8%; SD=14.8% - Bachelor’s degree M=58.5%; SD=18.0%   Graduate coursework/degree M=67.8%; SD=13.8% |
| **Information literacy tool**  **(1 study)** | J. Y. Sim, K. S. Jang, N. Y. Kim  (2016) [59] | South Korea  RNs |  |  |  |  |  |  | Not reported | Not reported | Not reported | #  Information search ability post-test scores were higher for intervention group (M=3.88; SD=0.67) versus control (M=2.82; SD=0.69) following EBP education program t=6.22; p<.001 |
| **Modified Korean EBM questionnaire**  **(1 study)** | J. W. Park, J. A. Ahn, M. M. Park  (2015) [134] | Korea  RNs |  |  |  |  |  |  | Not reported | Not reported | Not reported | #♦  t-test and ANOVA  **Perception of EBP**  Significant differences found based on:   - Work experience (t = −2.494, P = 0.013)   - ≤10 years (M=2.79; SD=0.26)   - >10 years (M=2.86; SD=0.26) - Education level (F=9.769, p<0.001)   - 3-year college (M=2.76; SD=0.28)   - Bachelor (M=2.82; SD=0.22)   - Master and PhD (M=2.94; SD=0.31) - Previous EBP education (t=2.454, p=0.015)   - Yes (M=2.86; SD=0.25)   - No (M=2.80; SD=0.26)   **Attitudes towards EBP**  Significant differences found based on:   - Work experience (t = −2.360, P = 0.019)   - ≤10 years (M=2.72; SD=0.31)   - >10 years (M=2.80; SD=0.33) - Education level (F=15.236, p<0.001)   - 3-year college (M=2.65; SD=0.27)   - Bachelor (M=2.76; SD=0.32)   - Master and PhD (M=2.92; SD=0.36) - Previous EBP education (t=1.972, p=0.049)   - Yes (M=2.80; SD=0.31)   - No (M=2.73; SD=0.33) |
| **Evidence-Based Practice Attitudes Scale (EBPAS)**  **(1 study)** | J. R. Duffy, S. Culp, K. Sand-Jecklin, L. Stroupe, N. Lucke-Wold  (2016) [32]  J. R. Duffy, S. Culp, C. Yarberry, L. Stroupe, K. Sand-Jecklin, A. Sparks Coburn  (2015) [31] | United States  RNs |  |  |  |  |  |  | Not reported | Not reported | Not reported | +#  Correlation   - Positive association between EBP use item (Participate in implementing research knowledge in practice) and EBP attitudes (ρ=0.299, p=.011)   Mann-Whitney U test   - Statistically significant difference in EBP attitudes by position type (p=.016):   - Nurse leaders (M=50.4, SD=5.5)   - Staff (M=46.4, SD=5.9) |
| **Attitudes to Evidence-Based Practice Questionnaire**  **(1 study)** | M. J. Linton, M. A. Prasun  (2013) [33] | United States  Licensure group not specified |  |  |  |  |  |  | Original findings reported | Not reported | Not reported | +  Significant positive correlations between individual ‘attitudes’ items and age and/or education:   - Confidence in my ability to evaluate quality of research   - Education (r=0.208, p=0.000) - Available research is not relevant   - Age (r=0.155; p=0.004) - Hard to influence changes in clinical practice   - Age (0.153; p=0.005) - Research findings are often not easily transferable   - Age (r=0.182; p=0.001) |
| **Nurses’ Attitudes Toward EBP Scale (NATES)**  **(1 study)** | Linda Thiel,  Yashowanto Ghosh (2008) [137] | United States  RNs |  |  |  |  |  |  | Not reported | Not reported | Original findings reported | +  Significant positive associations between NATES scores and:   - Education (rho = 0.248, p < 0.01) - Years in nursing (rho = 0.236, p < 0.01) - Age (rho = 0.210, p < 0.05) - Unit culture (rho = 0.626, p < 0.01) - EBP knowledge (rho = 0.379, p < 0.01) |
| **Single item measure for EBP knowledge**  **(1 study)** | B. Skela-Savic, S. Hvalic-Touzery, K. Pesjak  (2017) [109] | Slovenia  RNs |  |  |  |  |  |  | Not reported | Not reported | Not reported | +  Significant positive associations between EBP knowledge and:   - EBP beliefs (knowledge and skills on EBP) r=0.477; p<0.01) - EBP implementation (advanced forms of EBP implementation) r=0.331, p<0.01 - EBP implementation (initial forms of EBP implementation) r=0.292, p<0.01 |
| **Evidence-Based Practice Knowledge**  **Assessment in Nursing (EKAN)**  **(1 study)** | A. Hagedorn Wonder, A. M. McNelis, D. Spurlock, P. M. Ironside, S. Lancaster, C. R. Davis, M. Gainey, N. Verwers  (2017) [45] | United States  RNs |  |  |  |  |  |  | Not reported | Not reported | Not reported | *   - Educational level was a statistically significant predictor of EKAN scores F(1,149) = 30.43,   p<.001, R^2^ = .170) |
| **Knowledge Assessment Test (KAT)**  **(1 study)** | H. T. Xie, Z. Y. Zhou, C. Q. Xu, S. Ong, A. Govindasamy  (2015) [66] | Singapore  Licensure group not specified |  |  |  |  |  |  | Not reported | Not reported | Not reported | #♦  t-test  Significant difference in KAT scores based on prior EBP involvement t(58) = 2.41, p = 0.02  ANOVA  Nurses with degrees (M=15.15, SD=3.88) had better knowledge than nurses with diplomas (M=12.00; SD=2.81) F(3,58) = 3.28, p = 0.03 |
| **Core Knowledge Questionnaire**  **(1 study)** | B. M. Toole, J. F. Stichler, L. Ecoff, L. Kath (2013) [62] | United States  RNs |  |  |  |  |  |  | Not reported | Not reported | Not reported | +  Significant correlations between core knowledge and:   - EBPQ skills (r=.158, p<.01) - EBPQ attitude (r=.148, p<.01) - EBPQ practice (.090, p<.05) |
